# Tracing Arab-Islamic Inheritance in Madagascar: Study of the Y-chromosome and Mitochondrial DNA in the Antemoro

**DOI:** 10.1371/journal.pone.0080932

**Published:** 2013-11-22

**Authors:** Mélanie Capredon, Nicolas Brucato, Laure Tonasso, Valérie Choesmel-Cadamuro, François-Xavier Ricaut, Harilanto Razafindrazaka, Andriamihaja Bakomalala Rakotondrabe, Mamisoa Adelta Ratolojanahary, Louis-Paul Randriamarolaza, Bernard Champion, Jean-Michel Dugoujon

**Affiliations:** 1 Laboratoire d’Anthropologie Moléculaire et Imagerie de Synthèse, CNRS and Université Paul Sabatier Toulouse III, UMR5288, Toulouse, France; 2 Centre de recherche littéraire et historique de l’Océan Indien (CRLHOI), Département d’ethnologie, Université de La Réunion, Saint-Denis, France; 3 Department of Pediatrics, CHU Sainte Justine, Faculty of Medecine, University of Montreal, Quebec, Canada; 4 Language and Genetics Department, Max Planck Institute for Psycholinguistics, Nijmegen, The Netherlands; 5 Laboratoire d'Anthropologie Patrimoine -Transformations sociales- Transculturalité (LAP2T), Université Antananarivo, Antananarivo, Madagascar; University of Cambridge, United Kingdom

## Abstract

Madagascar is located at the crossroads of the Asian and African worlds and is therefore of particular interest for studies on human population migration. Within the large human diversity of the Great Island, we focused our study on a particular ethnic group, the Antemoro. Their culture presents an important Arab-Islamic influence, but the question of an Arab biological inheritance remains unresolved. We analyzed paternal (n=129) and maternal (n=135) lineages of this ethnic group. Although the majority of Antemoro genetic ancestry comes from sub-Saharan African and Southeast Asian gene pools, we observed in their paternal lineages two specific haplogroups (J1 and T1) linked to Middle Eastern origins. This inheritance was restricted to some Antemoro sub-groups. Statistical analyses tended to confirm significant Middle Eastern genetic contribution. This study gives a new perspective to the large human genetic diversity in Madagascar.

## Introduction

Many population movements took place across the western Indian Ocean, notably for colonial and commercial purposes, and these have been highlighted through linguistic, crop, cattle, archaeozoological and commensal archaeological data [1–4]. Madagascar possesses a large human genetic diversity inherited principally from Asia and Africa [5–8]. 

We were interested in a group inhabiting the southeast coast of the Great Island, which have known Arab-Islamic influence. Three major migrations have occurred in this part of the island that could be linked to this Arab-Islamic influence. It is probable that the Onjatsy settled first, followed by the ZafiRaminia around the 13^th^ century, and finally the Antemoro. This last migration may have taken place around the last quarter of the 15^th^ century. The Antemoro would have been able to root their beliefs and traditions in the region as they shared cultural and social similarities with the first migrants in the area [9–11]. 

A part of the Antemoro claims an Arab origin in Mecca. However, the reference to Mecca might not have referred to a geographic location in the Arabian Peninsula, but to a Muslim identity. Their traditions and history are written in the Sorabe Manuscripts, manuscripts written in Malagasy with Arabic characters. The Antemoro society has a hierarchical system in which political powers and magical and religious affairs are separated. Three main sub-groups can be distinguished according to this structure: the Anteony, the Antalaotra and the Ampanabaka. The Anteony are the descendants of aristocrats, from whom the Antemoro king is chosen. The Antalaotra are in charge of the magical and religious domains; they have the ability to read and write Sorabe [1,9,10,12]. These first two groups can be grouped into the Silamo, because they have the right to undertake the ritual slaughter of animals (Sombily). The third group, to which the Ampanabaka belongs, is the Kafiry. The Ampanabaka revolt against the government in the late 19^th^ century marked the end of the Antemoro kingdom [12–14].

Although the Arab-Islamic cultural influence is evident, the origins of the Antemoro are still disputed. Some authors state they came from eastern Africa, while others state they were Islamized Malays [1,9,14]. A previous work on an Antemoro population based on the immunological Gm system highlighted an African and a Southeast Asian origin but no Arabic traces [15]. To provide answers to this debate, the biological origins of the Antemoro populations were studied using the non-recombining region of the Y-chromosome (NRY) and mitochondrial DNA (mtDNA) polymorphisms. They were analyzed to determine if they differed from other Malagasy populations and to understand the limits of Arab-Islamic migrations. 

## Materials and Methods

### Ethics Statement

This work was approved by the ethics committee of the Ministry of Health in Antananarivo, Madagascar. The ethics committee authorization letter was presented and signed by each district officer where the sampling was conducted. The study was explained to each potential participant, in Malagasy language. Then volunteers gave their informed consent by signing a written informed consent form.

### Population sampling

**Figure 1:**
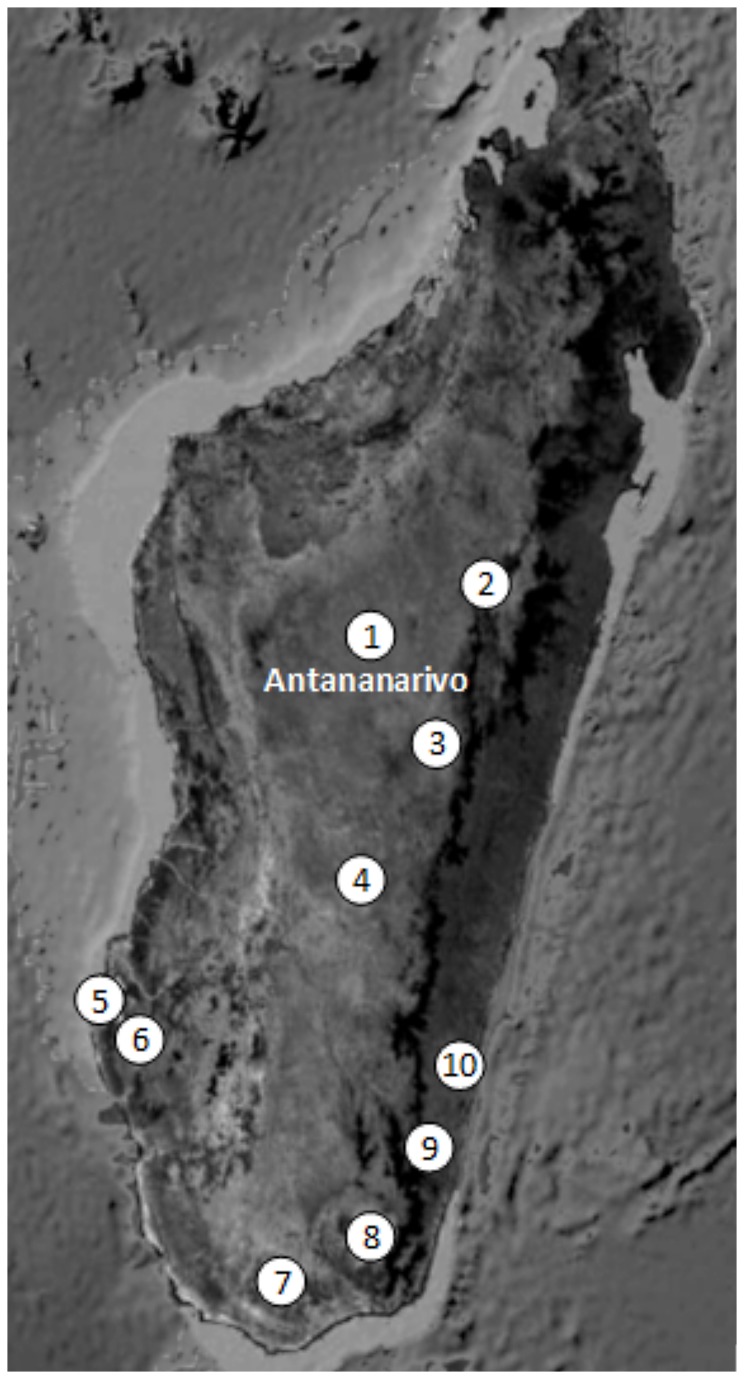
Repartition of the ethnic groups for which genetic data are available and that have been used in this study. 1 : Merina [5,6]; 2. Sihanaka; 3. Bezanozano; 4. Betsileo [5]; 5. Vezo; 6. Mikea [7]; 7. Antandroy; 8. Antanosy; 9. Antaisaka [6]; 10. Antemoro (this study). Populations from 1 to 4 were grouped in Highlands population.

Fieldwork took place in villages between Manakara and Vohipeno on the southeast coast of Madagascar (Figure 1). Genealogical investigations were performed in order to limit related participants. Saliva samples were collected from the three Antemoro groups: Ampanabaka, Antalaotra and Anteony.

### Laboratory methods

**Figure 2:**
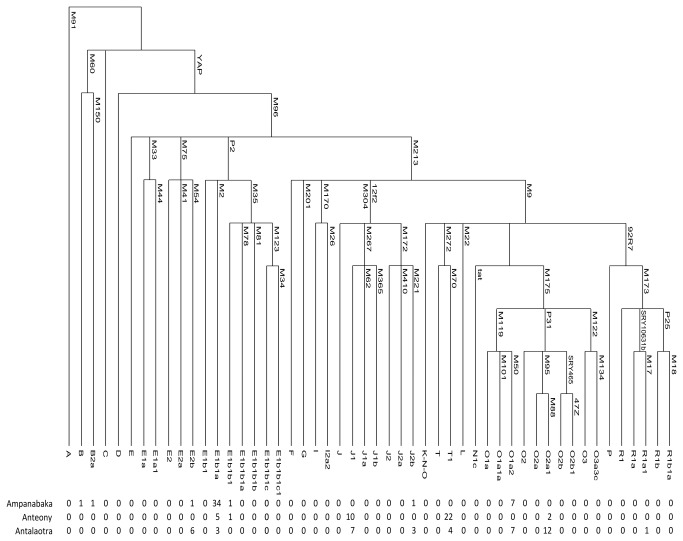
Absolute frequency of NRY haplogroups in the Antemoro. The maximum parsimony phylogeny relating the NRY haplogroups is label with markers that define them.

The extraction of DNA from saliva samples was performed following the protocol provided with the Oragene DNA kits (Oragene Genotek http://www.dnagenotek.com). DNA was typed for 17 Y-chromosome short tandem repeats (Y-STR) loci (DYS19, DYS389I, DYS389II, DYS390, DYS391, DYS392, DYS393, DYS385a, DYS385b, DYS437, DYS438, DYS439, DYS448, DYS456, DYS458, DYS635, GATAH4) using the AmpFlSTR ® Y-filer kit (Applied Biosystems http://www.invitrogen.com). A prediction of haplogroup assignment to each Y-STR profile was achieved using Haplogroup predictor (http://www.hprg.com/hapest5/). Y-chromosome Alu polymorphism (YAP) was also tested. Y- chromosome binary markers were analyzed to confirm the previous assignment. Initially all individuals were studied for the marker YAP [16]. YAP negative individuals (YAP-) were tested for binary markers: 92R7, M70, M22, Tat, P25, SRY1532, M173, M213, M9 [17]. Depending on the results, they were subsequently tested for haplogroup O ([5], Razafindrazaka unpublished data), haplogroups G,I,J [17], haplogroup J (this study), haplogroups A, B, B2a ([5], this study), haplogroup R1 [17] or haplogroup T ([18], this study). YAP positive individuals (YAP+) were tested for haplogroup E [5,17,19] (Figure 2). These polymorphisms were investigated by multiplex PCR to obtain simultaneous amplifications of multiple SNPs. They were typed by mini-sequencing using the MultiplexTM SNaPshot kit (PE Applied Biosystems http://www.invitrogen.com). All data were obtained on an ABI PRISM 3730 sequencer (PE Applied Biosystems) and analyzed with GeneMapper v.4.0 (PE Applied Biosystems http://www.invitrogen.com). The final haplogroup assignment followed the most recently updated NRY phylogeny (January 2011; http://www.isogg.org).

**Figure 3:**
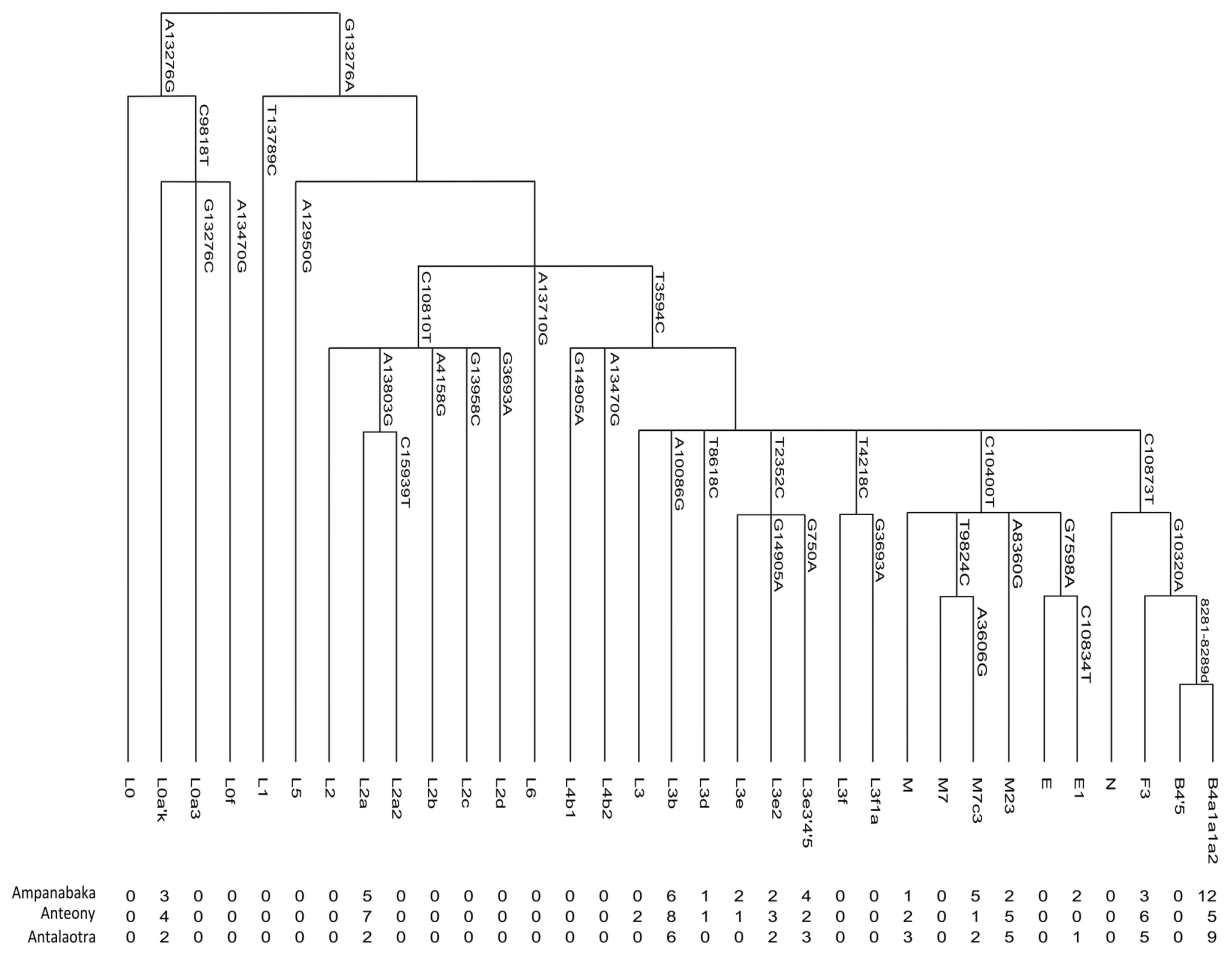
Absolute frequency of mitochonfrial haplogroups in the Antemoro. The maximum parsimony phylogeny relating the mitochondrial haplogroups is label with markers that define them.

The mtDNA was analyzed for hypervariable regions 1 and 2 (HVI and II) in the D-Loop. These regions were amplified with the primers L15973 (5'-AACTCCACCATTAGCACCCA-3 ') and H296 (5'-TC TGTAGTATTGTTTTTAAAGG-3') [20]. Data were obtained on the automated sequencer ABI PRISM 3730 (PE Applied Biosystems). The sequences were corrected using the Sequencing Analysis software v5.3 and aligned to the revised Cambridge reference sequence (rCRS) [21,22] using the CustalW algorithm in BioEdit. The consensus sequence of each sample was analyzed individually and polymorphisms were characterized. Haplogroups were assigned by the online software Haplogrep (http://haplogrep.uibk.ac.at/) and Mtmanager (http://mtmanager.yonsei.ac.kr/). Specific mutations of mtDNA were tested in the coding region to confirm the haplogroup assignment for each sequence. This step was carried out by the RFLP (Restriction Fragment Length Polymorphism) technique. All individuals were tested for SNPs associated with haplogroup L3, M and N. According to the previous estimations from the hypervariable regions, SNPs were tested for haplogroups M7, M7c3, E, E1, F3 and B4a1a. Mutation at nucleotide (nt) A8360G associated with haplogroup M23 was tested by sequencing this region. In addition, for individuals estimated to belong to haplogroup B, confirmation was achieved by reading a 9 bp deletion in region V. Two individuals did not have the mutation 16247 on the HVI region, which defines haplogroup B4a1a1a (Polynesian motif: nt A14022G , nt T16217C, nt A16247G, and nt C16261T), so they were tested for the mutation at nt A14022G by SNaPshot to confirm that they belonged to haplogroup B4a1a1. Finally, they were all tested by RFLP for a SNP (nt C1473T) that determines the B4a1a1a2 Malagasy motif [7]. For African haplogroups, confirmation of the estimated haplogroup was made by the SNaPshot® Multiplex Kit protocol: L0, L0a3, L1, L4, L0f, L2'6, L5, L3'4, L6, L0a'k, L2a, L2a2, L2b, L2d, L2c, L3b, L3d, L3e2, L3f, L3e, L3e3'4'5 [19] (Figure 3).

### Data Analysis

#### NRY data analysis

129 NRY were analyzed (Figure 1; Table S1 in File S1). All statistics on NRY haplogroups and haplotypes, intra-population genetic diversity [23], F_ST_ [24] and AMOVA were performed using ARLEQUIN 3.5.1.2 [25].

Four databases were created for the study of paternal lineages (Tables S2-S5 in File S1). One database compiled Y-haplogroup data available in the literature; it held 131 populations (16892 individuals) and included populations with a large number of individuals (over 50) and some populations from regions of interest. The second database included Y-STR haplotypes based on seven markers (minimal haplotype: DYS19, DYS389I, DYS389II, DYS390, DYS391, DYS392, DYS393), and contained 90 populations (13582 individuals). These populations are distributed across various regions: southern, central and western Africa (including Mozambique), eastern Africa, northern Africa (including Egypt), the Middle East (including Iran, Turkey and Cyprus), Europe, South Asia, Southeastern Asia, Oceania (Polynesia and New Guinea), the Comoros and Madagascar. For the last two regions, analyses were also made on the 17 Y-STR markers. The third and the fourth databases were created using data specific to two haplogroups found in our populations J1 and T, in order to achieve median-joining networks. With the aim of expanding the database on haplogroups J1 and T, haplogroups were estimated based on minimal haplotypes with Haplogroup Predictor (www.hprg.com/hapest5/). We also used information from the YHRD database (http://www.yhrd.org/). We considered haplotypes when their probability of belonging to haplogroup J1 or T was above 90%. Few data are available for the T haplogroup, as its definition is relatively recent [18]. Haplogroup T, previously K2, was in the branch of haplogroup K*. Therefore it has not been systematically tested.

Population pairwise F_ST_ values were estimated to determine the level of genetic differentiation [26] between Madagascar and the Comoros populations, based on 17 Y-STR markers. Results were visualized on a multidimensional scaling (MDS) plot, performed using XLstat.V7.5 to estimate the degree of differentiation between populations. A second MDS and a Principal Component Analysis (PCA) were performed using the haplogroup frequencies of populations in various geographic regions. The latter analysis was performed with XLstat.V10.6. Some haplogroups were grouped phylogenetically if they had a small contribution to the first two axes of the PCA. Shared haplotypes were analyzed between Antemoro groups and other Malagasy population based on 17 Y-STR and among populations from various geographic regions based on 7 Y-STR. 

Rate of admixture analysis was performed with AdmixV2.0 (mY; [27,28]) on the haplogroup frequencies distributed in the eight hypothetical parental populations (southern, central and western Africa, eastern Africa, northern Africa, Middle East, Southern Asia, Southeastern Asia, Oceania, Europe). The validity of this structure was tested firstly by AMOVA. A second estimation was achieved by Maximum Likelihood (MLE) using Leadmix [29]. In this case the parental populations were selected from the results of the previous analysis and by using the Y-STR minimal haplotypes. Finally, we looked for shared haplotypes between the Antemoro and populations from our database with ARLEQUIN 3,5.1.2 .Two median-joining networks were performed with Networks V4.6 for haplotypes belonging to J1 and T1 using the minimal haplotype (DYS19, DYS389i, DYS389ii, DYS390, DYS391, DYS392, DYS393) to link Antemoro lineages to other individuals from various geographic areas. DYS389ii was obtained after subtracting DYS389I from DYS389II.

#### mtDNA data analysis

135 mtDNA were analyzed (Table S6 in File S1) (Genbank accession numbers: KF716178-KF716312). Analyses were performed using ARLEQUIN 3.5.1.2 [25]. Genetic diversity was calculated based on HVI and HVII (16024-236). The populations’ pairwise F_ST_ values between Antemoro and other Malagasy people were computed on HVI (nt16065-16363) and represented by a MDS plot. Haplogroup frequencies were visualized on a PCA plot. Finally, data obtained from the HVI were compared with data available in the literature for Malagasy populations, southern, central and western Africa, eastern Africa, northern Africa, Middle East, south Asia, parts of southeast Asia, Oceania and Europe (Table S7 in File S1). The database included 107 populations (13544 individuals). Shared haplotypes and F_ST_ values were computed based on HVI haplotypes and F_ST_ visualized by MDS.

## Results

### Paternal lineages

#### Antemoro genetic diversity

The Ampanabaka (h = 0.98 ± 0.014; 36 haplotypes based on 17 Y-STR) and the Antalaotra (h = 0.98 ± 0.009; 35 haplotypes based on 17 Y-STR) have population genetic diversities within the same order of magnitude as those found generally in populations from southern Madagascar (0.95 ≤ h ≤ 0, 99) [30]. In contrast, the Anteony (h = 0.91 ± 0.028; 21 haplotypes based on 17 Y-STR) were less diverse (Figure 2). Pairwise F_ST_ values between Antemoro populations using 17 Y-STR markers revealed that the Ampanabaka group was strongly differentiated from the Antalaotra (F_ST_ = 0.158, p-value <0.01, Table 1) and the Anteony (F_ST_ = 0.205, p-value <0.01, Table 1). The comparison of the Antalaotra and Anteony also showed significant genetic differentiation (F_ST_ = 0.124, p-value <0.01, Table 1). Furthermore, the Ampanabaka did not share any haplotypes with the other two groups. However, the Anteony and Antalaotra shared three haplotypes, corresponding to the haplogroups J1, T1 and E1b1a1.

**Table 1:** Population pairwise F_ST_ calculated from 17 Y-STR between Malagasy and Comoros populations.

	**Ampanabaka**	**Anteony**	**Antalaotra**	**Antandroy**	**Antanosy**	**Comoros**	**Mikea**	**Northern Vezo**	**Southern Vezo**
**Ampanabaka**	0.00000	+	+	+	+	+	+	+	+
**Anteony**	0.20530	0.00000	+	+	+	+	+	+	+
**Antalaotra**	0.15800	0.12494	0.00000	+	+	+	+	+	+
**Antandroy**	0.07332	0.20911	0.12777	0.00000	+	+	+	+	-
**Antanosy**	0.04980	0.13650	0.07099	0.02220	0.00000	+	+	+	-
**Comoros**	0.08777	0.13985	0.06589	0.08002	0.03017	0.00000	+	+	+
**Mikea**	0.06677	0.19053	0.09262	0.06218	0.01633	0.03961	0.00000	+	+
**Northern Vezo**	0.05228	0.26205	0.17919	0.03440	0.03511	0.08297	0.04192	0.00000	-
**Southern Vezo**	0.02510	0.20609	0.11817	0.01494	0.01370	0.06190	0.02717	0.01954	0.00000

#### Comparison with Malagasy and Comorian populations

**Figure 4:**
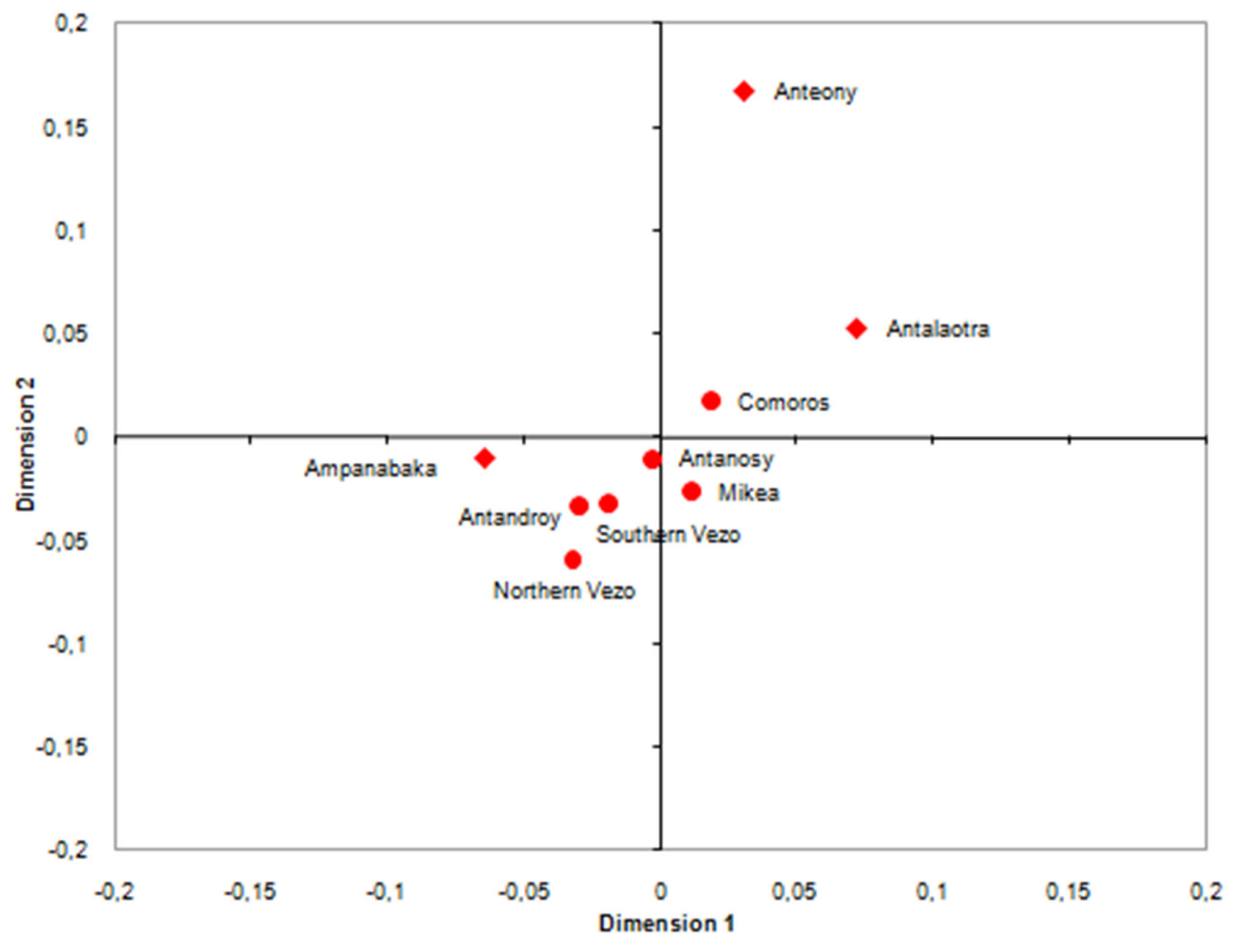
MDS plot computed from F_ST_ values between Malagasy populations and the Comoros based on 17 Y-STR data.

The Ampanabaka is the only group which showed low F_ST_ values ​​(F_ST_ <0.05, p-value <0.01, Table 1) compared with the other non-Antemoro Malagasy groups (the Antanosy and Vezo from southern Tulear; Figure 4 and Table S8 in File S1). The Ampanabaka are grouped with other populations from southern Madagascar, while the Antalaotra and Anteony are excluded.

Haplotype sharing analysis with 17 markers showed that the Anteony and Antalaotra did not share any Y-STR profiles with the Malagasy and Comorian populations. The Ampanabaka shared two profiles (E1b1a and E2b) with one Antandroy and one Antanosy individual, respectively from the southern coast of the Great Island. Three Malagasy populations were not considered for statistical calculations due to the low number of individuals in the sample (N <15; the Merina, Tsimahafotsy and Antaisaka), and the Andriana was excluded as it is an inbred population [6,30]. Nevertheless, the search for shared haplotypes using the 17 Y-STR markers revealed that no lineages were shared between these groups and our Antemoro groups (Tables S9-S11 in File S1).

A MDS plot based on the haplogroup frequencies confirmed these clusters (Figure S1 in File S1). The Antalaotra appeared poorly differentiated from Malagasy Highlands population (F_ST_ = 0.041, p-value <0.05). Some Antemoro groups differ from other populations from southern Madagascar [6], the southwest coast [30] and the Highlands [5] probably due to the presence of the haplogroups J1 and T1. However, J1 has not been tested in Highlands population. The Antalaotra and the Anteony have a more divergent paternal genetic diversity compared with the other groups analyzed (F_ST_ > 0.05, p-value <0.01, Table 1).

#### Geographic comparison

Haplogroup O1a2 was found at 16% and 19% in the Ampanabaka and Antalaotra, respectively but it was absent in the Anteony group. Haplogroup O2a was found at around 26% and 5% in the Antalaotra and Anteony respectively, and was absent in the Ampanabaka. These haplogroups are markers of Austronesian migration. O1a2 is found in Indonesian populations in the Southeast Asian islands and at lowest frequency in Oceania [31–33]. Haplogroups O2 and O2a1 are most common in parts of Southeast Asia [31,33], but are also found in India [34]. Haplogroup E1b1a1 was the most represented haplogroup in the Malagasy population [6,30]. It constituted 76% of the Ampanabaka genetic diversity, but only 7% in the Antalaotra and 12% in the Anteony. This haplogroup is very common in African populations. The highest frequencies are found in western Africa [35–37]. Haplogroup E1b1b1 was found in two individuals (one Anteony and one Ampanabaka). This haplogroup is generally attributed to eastern Africa [35] and it is also found at moderate frequencies in North Africa and the Middle East. Haplogroup E2b was present in an Ampanabaka individual and was present at 7% in the Antalaotra. This haplogroup is also attributed to Africa [38], but has been observed at low frequencies among the Arabs of Oman and Qatar [36,39]. Finally haplogroups B and B2a, found in two Ampanabaka individuals, have an origin in sub-Saharan Africa [18,36]. Haplogroup R1a1was found in one Antalaotra individual. This haplogroup is widely distributed in Europe, with high frequencies in eastern Europe and India, and with a recent introduction into Saudi Arabia linked to historic trade routes [40,41]. Haplogroup J2 was present at a frequency of 2% in the Ampanabaka and 7% in the Antalaotra. The J1 clade (xJ1a, xJ1b) was absent in the Ampanabaka group, but present in the Antalaotra (16%) and the Anteony (25%). Haplogroup J is the most common haplogroup in the Middle East [42]. J2 haplogroups would have diffused northward in Europe and Asia [36,43]. J1(xJ1a, xJ1b) is a marker of recent Arab expansion in the Arabian Peninsula, and the northern and northeastern parts of Africa [43]. It has been observed at very low frequencies in southern and Southeast Asia [33,34]. Haplogroup T1 was not found in the Ampanabaka group, only in the Antalaotra (9%) and the Anteony (55%) groups. Clade T is rare but it has a very broad distribution. T1 is found mainly in the Middle East (Palestine, Lebanon, Oman, Turkey, southern Iran), North Africa (Egypt, Morocco), sub-Saharan Africa (especially in eastern Africa: Ethiopia, Sudan, Tanzania, Uganda), and Europe [18,36,44–49]. It has also been described in India and China [34,40,50].

The search for shared haplotypes (Tables S12-14 in File S1) was based on the Y-STR core haplotype database. Although it contains an obvious bias due to the relatively low number of markers raising the probability of homoplasy, it allows a wide spectrum of possible shared haplotypes to be determined. It showed that the haplotypes corresponding to haplogroup E1b1a were found mainly in people from southern, central and western Africa. The Ampanabaka haplotype belonging to E1b1a was found in one population out of eastern Africa, two populations from northern Africa and five populations from the Middle East. It was also found in two populations from Southeast Asia, but these haplotypes did not belong to haplogroup E due to the previously explained bias. The Anteony haplotype belonging to E1b1b1 haplogroup was found in populations from Africa, the Middle East and Europe. Haplotypes belonging to E2b haplogroup are shared with people from Africa and from some Middle Eastern populations. B haplotypes were found in African samples. Haplotypes under haplogroup O1a2 were found mainly in Southeast Asia. Antemoro haplotypes in the background of haplogroup O2a1 were shared with individuals from Southeast Asia and India. We should note that a haplotype belonging to O2a1 haplogroup was also found in Uganda and another in Europe. Haplotypes belonging to R1a1 were found in a population from northern Africa, a population from Southeast Asia, four populations from the Middle East and in six Indian populations. It is probable that its arrival in Madagascar is linked with trade movements between Arabia and India [41]. Haplotypes associated with J2 were found in eastern Africa, the Middle East and India. The ones associated with J1 were shared with various populations from northern Africa (2), the Middle East (6), India (3) and Europe (1). Finally, haplotypes corresponding to T1 haplogroup were shared with a population from Africa and various populations from the Middle East (6), Southeast Asia (6) and Europe (1). We note that no T haplogroup was described in our database of haplogroup frequencies in Southeast Asia. Thus, these haplotypes may correspond to lineages from closed haplogroups (S1; O2b1a, M1a and M). This is also the case for the R1b haplogroup found in Europe. Thus, four lineages (T1, J1, J2 and E1b1b1) were found mainly in the Middle East regions.

F_ST_ values based on Y-chromosome haplogroup frequencies (Table S8 in File S1) were computed and visualized on a MDS plot (Figure S2 in File S1). It revealed that the Ampanabaka present a low to moderate differentiation from western, central and southern African populations (0.036 < F_ST_ <0.152). The Antalaotra are genetically very heterogeneous. They were close to many populations from various geographic areas, although no value was below 0.05. We noted that the lowest values were found when the Antalaotra were compared with populations from Southeast Asia, such as Java and Sulawesi (0.067 < F_ST_ <0.072). The Anteony were highly differentiated from all populations in the database. The lowest values were observed with populations from Oman and the UAE (0.142 < F_ST_ < 0.155), although it should be noted for comparison that individuals of the Highlands and the Comoros had F_ST_ values relatively similar to those of these two populations.

**Figure 5:**
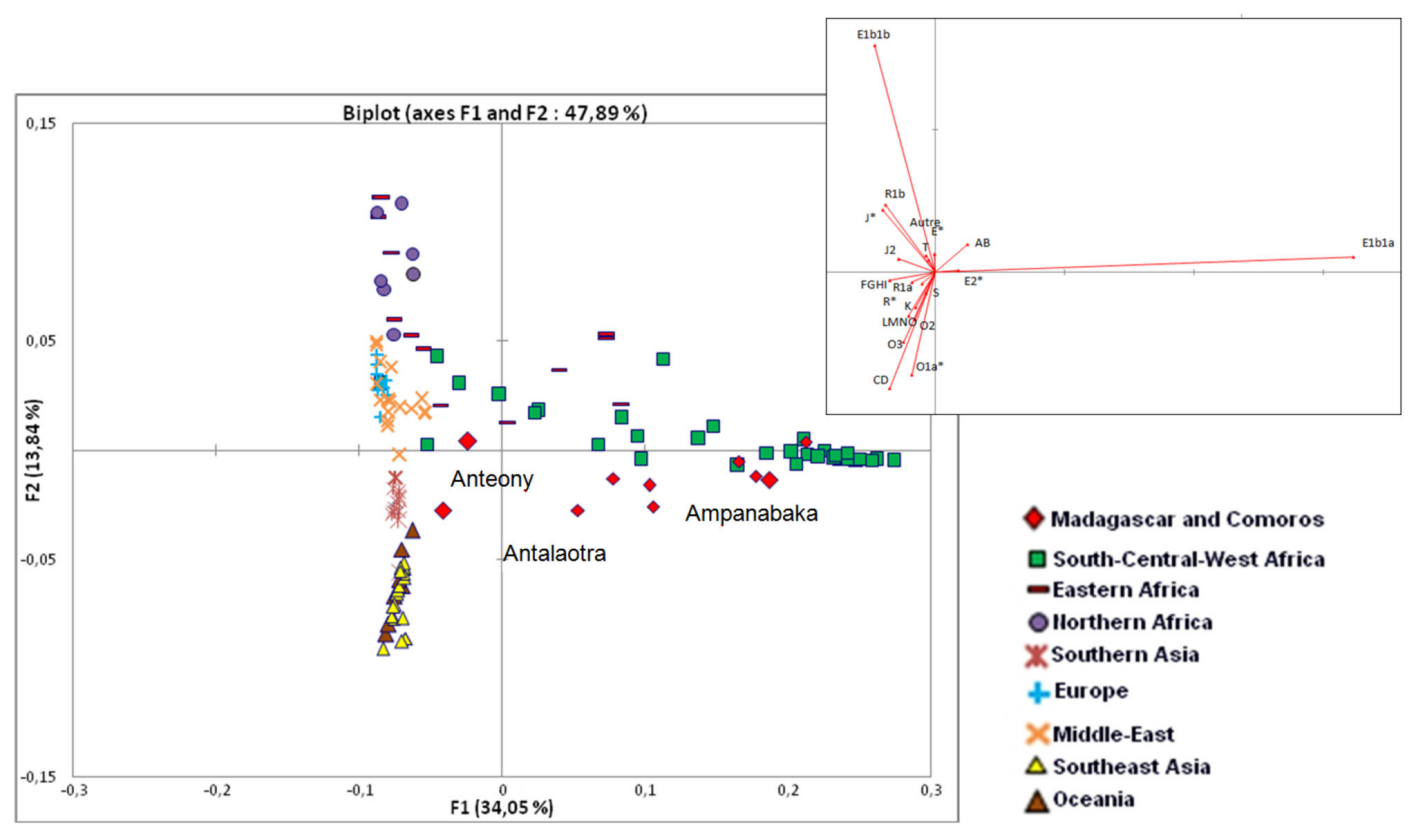
Principal Component Analysis plot computed from NRY haplogroup frequencies of the Antemoro and populations from the database. The insert showed the contribution of haplogroups to the two components.

In addition to F_ST,_ a PCA was computed from haplogroup frequencies (Figure 5). The first and second axis represents 47.91% of the variability. The haplogroup E1b1a contributes to 89% of the variability of the first axis, while the rest contributes to less than 1%. The haplogroups E1b1b and CD contribute 53% and 10% respectively to the variability represented by the second axis. The PCA shows the clustering of the Ampanabaka with southern, central, and western African populations and proximity to other Malagasy populations, due to the high frequency of the haplogroup E1b1a. The Antalaotra have a genetic diversity similar to that of Southeast Asia and Oceania (especially populations speaking Austronesian languages), due to the presence of the haplogroups O1a and O2. The Anteony appear at the crossroads of all these genetic diversities and are isolated due to the high frequencies of the haplogroups J1 and T1 (J * and T on the PCA).

#### Admixture

The presence of haplogroups with Middle Eastern genetic origins in the Anteony and Antalaotra, which were not found in other the Malagasy populations used in our comparisons, suggests a ‘Malagasy background’ with Arabian traces in the Antemoro. Two admixture estimates were carried out using haplogroup frequencies (mY) or Y-STR core haplotypes (MLE) of the three Antemoro groups and eight parental populations, geographic groups that could have directly or indirectly affected the genetic diversity of the Antemoro. The structure of these groups was validated by an AMOVA test (F_CT_ = 0.238; F_SC_ = 0.122, p-value < 0.001).

The MLE and mY estimations, based on two different databases, converge toward the same interpretation. Although the percentages are different, considering the intervals of confidence, the tendencies of contributions are similar (Table 1). They show that two geographic groups are present in the Ampanabaka gene pool: the western/central/southern Africa group and Southeast Asian. It appears that three regions contribute to the gene pool of the Antalaotra and Anteony. In the Antalaotra group, we found African contributions, especially from eastern Africa, Southeast Asia and the Middle East. In the Anteony group there were the same contributions but in different proportions (Table 2).

**Table 2:** Admixture rates in the three Antemoro groups calculated from eight hypothetical parental populations.

		**Southern, central, western Africa**	**Eastern Africa**	**Northern Africa**	**Middle-east**	**Southern Asia**	**Southeastern Asia**	**Europe**	**Oceania**
**Ampanabaka**	mY*	0.948± 0.075	0.000	0.000	0.000	0.000	0.052±0.075	0.000	0.000
	MLE*	0.745	-	-	0.000	-	0.255	-	-
	**+95%CI**	0.747					-		
	**-95%CI**	0.676					-		
**Antalaotra**	**mY**	0.000	0.027±0.051	0.000	0.429±0.103	0.000	0.544±0.079	0.000	0.000
	**MLE**	-	0.254	-	0.250	-	0.496	-	-
	**+95%CI**		0.404		0.395		-		
	**- 95%CI**		0.251		0.250		-		
**Anteony**	**mY**	0.000	0.106±0.072	0.000	0.697± 0.097	0.000	0.197± 0.052	0.000	0.000
	**MLE**	-	0.249	-	0.273	-	0.478	-	-
	**+95%CI**		0.431		0.501		-		
	**-95%CI**		0.001		0.261		-		

* mY is the estimated admixture rate from Y-haplogroup frequencies using AdmixV2.0. MLE is the admixture rate from haplotypes frequencies computed with Leadmix; CI values, are MLE confidence intervals.

#### Median-Joining Networks

**Figure 6:**
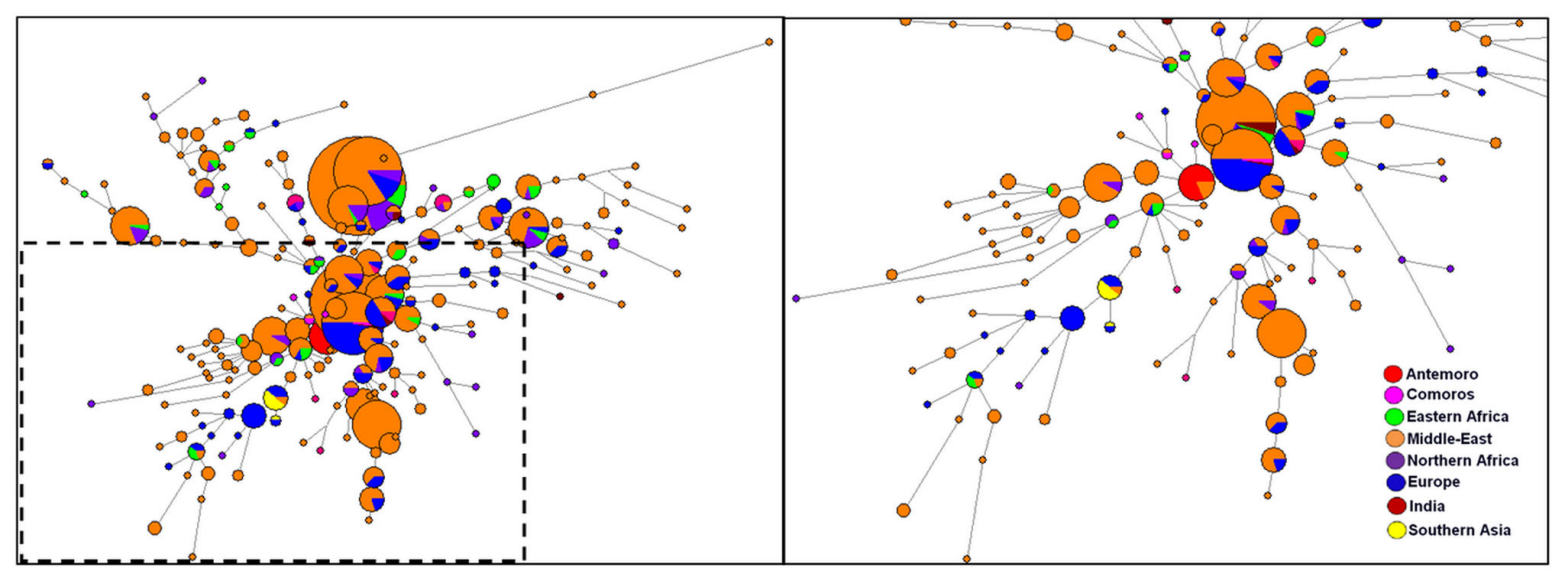
Median-Joining network computed from Y-STR minimal haplotypes (DYS19, DYS389i, DYS389ii, DYS390, DYS391, DYS392, DYS393) in the Antemoro and populations from various geographic regions belonging to haplogroup J1. Circles are haplotypes. Sizes of these circles are proportional to haplotype frequency and branch lengths are proportional to number of differences between haplotypes.

**Figure 7:**
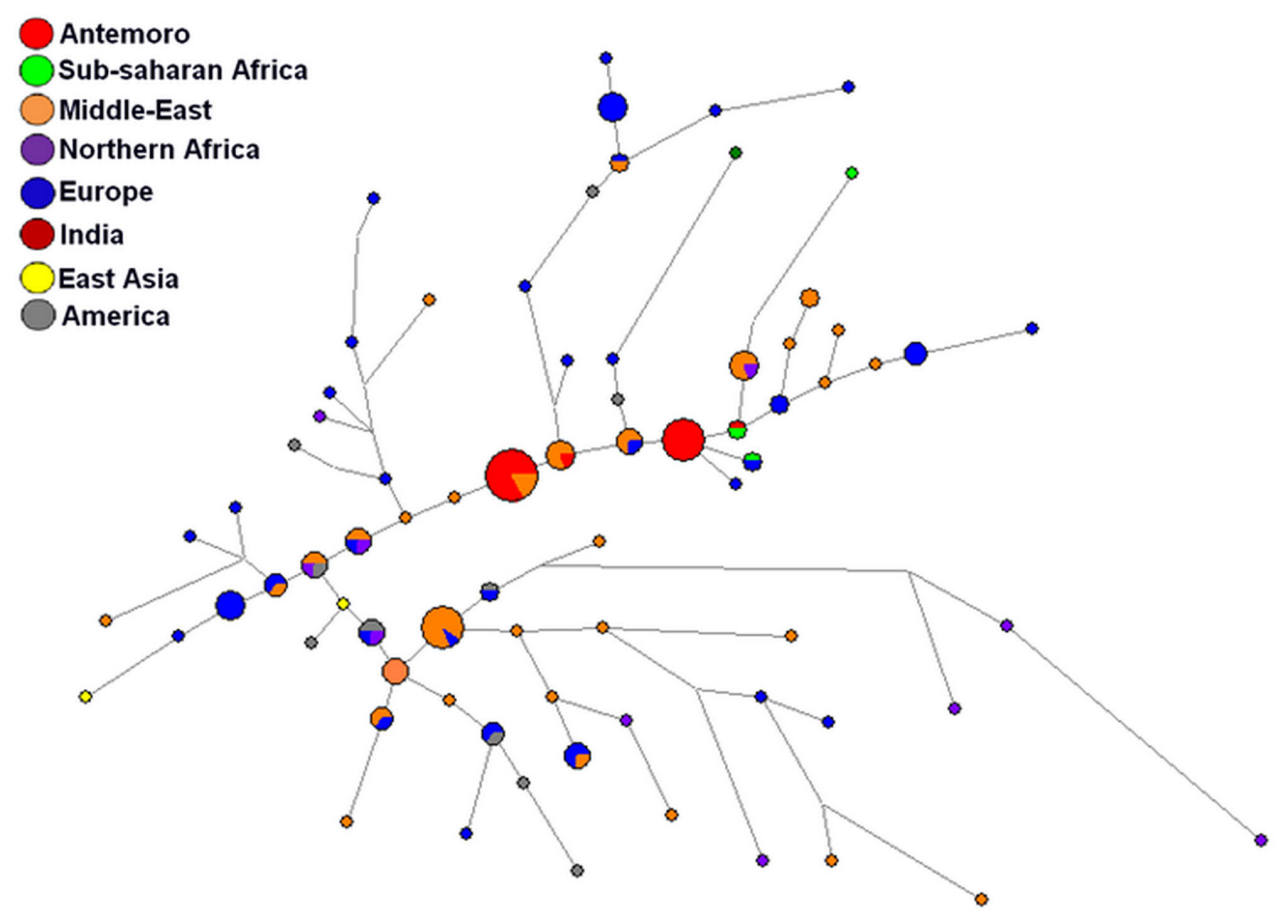
Median-Joining network computed from Y-STR minimal haplotypes (DYS19, DYS389i, DYS389ii, DYS390, DYS391, DYS392, DYS393) in the Antemoro and populations from various geographic regions belonging to haplogroup T. Circles are haplotypes. Size of these circle are proportional to haplotype frequency and branch lengths are proportional to number of differences between haplotypes.

Two Median-Joining Networks were computed using data on the minimal haplotypes (DYS19, DYS389i, DYS389ii, DYS390, DYS391, DYS392, DYS393) in order to localize the Antemoro haplotypes belonging to clades J1 and T, relative to the haplotypes of these same clades in various geographic areas (Figures 6 and 7). By using the minimal haplotype, the Antemoro diversity of the haplogroup J1 is reduced to one haplotype. It was observed that this lineage was not positioned at the end of a branch (Figure 6). This haplotype is found in populations from the Middle East (Cyprus, Turkey and Palestine). On the network, the Antemoro were connected to individuals from Turkey, Cyprus, Palestine, Comoros, Dagestan, Iraq, Italy, Portugal, Qatar, Kuwait, southern Pakistan, Syria, Israel, Lebanon, Arabic, Comoros, Saudi Arabia, Ethiopia and Portuguese Jews. For haplotypes belonging to clade T (Figure 7), it also appeared that the Antemoro were not positioned at the ends of branches. Two Antemoro haplotypes were found in the Middle East (Israel, Lebanon and Palestine). Another haplotype was similar to an individual from Angola. These lineages were linked to individuals from Israel, Spain and Lebanon and on the outgoing branches Antemoro lineages were connected to individuals from Europe, Brazil, Zambia, northern Africa and Lebanon. 

### Maternal lineages

#### Antemoro genetic diversity

The Ampanabaka revealed the lowest genetic diversity (h= 0.9379 (+/-0.0213); 22 haplotypes), while the Anteony have the highest (h= 0.9741 (+/- 0.0095), 29 haplotypes) and the Antalaotra have a genetic diversity indice in between (h=0.9513 (+/- 0.0189), 22 haplotypes). The distribution of mitochondrial haplogroups revealed the presence of haplogroups from Southeast Asia usually found in Madagascar. We found the haplogroup B4a1a1a2 that is associated with the Malagasy motif (nt C1473T) [7]. It was present at 25% in the Ampanabaka, 23% in the Antalaotra and 11% in the Anteony (Figure 3). 

There was no significant difference in the maternal genetic diversity among the three Antemoro groups (-0.00918 < F_ST_ <0.00659, p-value not significant). They shared six mitochondrial haplotypes (D-Loop: 16024-236, combined HVI and HVII). The Ampanabaka shared six haplotypes with the Anteony and two with the Antalaotra; these last two groups shared only two haplotypes.

#### Comparison with Malagasy populations

The HVI genetic diversity comparison between the Antemoro and other Malagasy groups for which data were available were represented by a MDS plot (Table S15 in File S1 and Figure S3 in File S1). It showed that our three groups were not very differentiated. The p-values of the F_ST_ values were not significant at the 0.01 threshold for any of the Malagasy groups, except between the Anteony and Highlands groups, but these were still not highly differentiated (F_ST_ = 0.003, p-value <0.01. It should be noted that in this case the Highlands group included data from Hurles [5] (n= 37), the Merina (n = 9) from Tofanelli [6] and the Tsimahafotsy (n = 6) from Razafindrazaka [30] in order to obtain a sufficient number of individuals (Figure 1). This low genetic differentiation was confirmed by a PCA analysis between Malagasy and Comoros populations (Figure S4 in File S1).

Haplotyping sharing analysis showed more than half of the haplotypes based on HVI in the Antemoro were found in other Malagasy groups. They shared ten haplotypes with the Highlands and Andriana groups, 18 with southern coastal groups and 18 with southwestern coastal groups (Tables S16-S18 in File S1).

#### Geographic comparison

Haplogroup E1a1a constituted 4% of the haplogroup diversity in the Ampanabaka, 3% in the Antalaotra and was absent in the Anteony. Its origin has been attributed to the Islands in Southeast Asia (ISEA) [51]. Haplogroup F3b was present at 6% in the Ampanabaka and 13% in the two other groups. This haplogroup was found in Southeast Asia, and has been described mainly in the Philippines and Borneo [52]. Two haplogroups belonging to clade M were highlighted. The haplogroup M7c3c is an Asian haplogroup. It was found at 10%, 5% and 2% in the Ampanabaka, Antalaotra and Anteony, respectively. These haplogroups, along with O1a for Y lineages, are markers for the ‘Out of Taiwan’ expansion to Borneo during the mid-Holocene [52]. The haplogroup M32c, formerly known as M46 [52], was found at 2% in the Ampanabaka, 8% in the Antalaotra and 2% in the Anteony. Another clade belonging to the haplogroup M was deduced from the HVI and HVII regions as the branch Q1. It was found in one Anteony individual. This is a very common haplogroup in New Guinea and Melanesia [53]. Finally, haplogroup M23, belonging to the M branch, was found at a frequency of 4% in the Ampanabaka, 13% in the Antalaotra and 11% in the Anteony. The origin of this haplogroup is not clearly defined, although a west Eurasian contribution had been postulated. The haplogroup M23 is relatively ancient and is found throughout Madagascar [8]. This Asian component was associated with a significant African component. We highlighted lineages belonging to the clades L0, L2 and L3 (xMN). L0a1'4 was present at 6% in the Ampanabaka, 5% in the Antalaotra and 8% in the Anteony. It was found at various frequencies throughout the African continent. The subdivision L0a is found mainly in the south, with the highest frequencies in Ethiopia [54,55]. It has been described in coastal populations of southeastern Madagascar [6]. The haplogroup L2a1 represented 10%, 5% and 15% of the diversity in the Ampanabaka, Antalaotra and Anteony, respectively. The haplogroup L2a is the most common haplogroup in Africa [56,57]. Finally, the clade L3 was the most represented clade: 31% in the Ampanabaka, 28% in the Antalaotra and 36% in the Anteony. The highest frequencies were found in northern and eastern Africa, but it was ubiquitous on the continent. We distinguished the branch L3a, found in an Anteony individual, defined by the positions 152 and 16316 on the D-Loop. It was found throughout the African continent with the highest frequencies in East Africa [57]. The L3b (Ampanabaka 13%, Antalaotra 15% and Anteony 17%) and L3d (Ampanabaka 2%, Anteony 2%) were most common in western and northern Africa [55,57]. The haplogroup L3e (Ampanabaka 16%, Antalaotra 13% and Anteony 12%) is the oldest L3 clade [57] and has an origin in central Africa and/or Sudan [58]. Finally, an Anteony individual was defined as L3k, by the mutation in 235 of HVII. It was found in individuals from northern Africa [55].

Regarding shared haplotypes using the HVI data (Tables S19-S21 in File S1); we found that haplotypes belonging to haplogroup B4a1a1 were shared with people from Southeast Asia and Oceania. M7c3c haplotypes were also found in Southeast Asian and Oceanian populations. One of our M32c haplotypes was found in two Southeast Asian populations. Haplotypes corresponding to the branch E1a were not shared by any of the individuals of our database. The estimated haplotype Q1 did not share any haplotype with populations from Asia or Oceania in our database, regions where this haplotype is normally found. However, it corresponds to a HVI/HVII sequence found in Saudi Arabia potentially belonging to haplogroup Q1. In fact the presence of this haplogroup in Arabia was probably due to recent arrivals in the first half of the 20^th^ century when many Indonesian women were hired for labour [59]. Concerning M23 haplotypes, one of the sequences corresponded to an individual in Dubai set to M*.

For the L clade, Ampanabaka haplotype L0a was found in populations from southern Africa, eastern Africa and the Arabian Peninsula. L2a1 haplotypes were found throughout the African continent and some were present in the Middle East. Concerning the clade L3, one L3b haplotype was found in Guinea-Bissau and Saudi Arabia, and the other was found in Berbers from Morocco and the Hadza from Tanzania. The L3d haplotype was shared by individuals from seven populations from central and southwest Africa, two populations from east Africa and three from the Middle East. L3e1a and L3e1b were found mainly in central and southwest Africa but also in some populations from eastern and northern Africa. One L3e3 haplotype was shared with African and Middle Eastern populations. Finally, the L3k haplotype was not found in our database.

F_ST_ values were calculated between the Antemoro, Malagasy populations and populations from various geographic regions (central, western and southern Africa, eastern Africa, northern Africa, the Middle East, southern Asia, Southeast Asia, Oceania, Europe) (Table S15 in File S1). We observed low levels of differentiation between the Antemoro and populations from Southeast Asia such as in Banjarmasin (South Kalimantan), Malays from Singapore and Kuala Lumpur, Sumatra and the Philippines (0.025 < F_ST_ <0.05, p-value <0.05). The Ampanabaka and Antalaotra had low differentiation from African populations (Senegal, Kenya, Ethiopia) (0.04 < F_ST_ <0.057, p-values <0.05). The Anteony had a more important African component. They showed low differentiation (0.028 < F_ST_ <0.050, p-value <0.05) from populations from eastern Africa (Sudan, Ethiopia, Kenya), central and western Africa (Senegal, Guinea Bissau, Benin, Ivory Coast) and northern Africa (Morocco, Tunisia, Egypt). Finally, these three groups had low F_ST_ values when they were compared with individuals from Dubai, Yemen (0.035 < F_ST_ <0.055) and northeast India (Tripura) (0.056 < F_ST_ <0.064). These results were visualized on the three MDS plots calculated for each major geographic region (Figure S5 in File S1); in each, Malagasy populations were clustered together.

## Discussion

### Comparison of NRY results and historic data

The analysis revealed that paternal lineages in the three Antemoro groups were highly differentiated from each other (Table 1). The genetic diversity of the Anteony was low. A low genetic diversity within a population suggests that the population would have experienced few migrations, but important genetic drift or selection pressures. In this study, inbreeding associated with a patrilineal society may have contributed to a reduction of the diversity. Historical factors should have also played a role in the reduction of the genetic diversity. In addition to the arrival of a small number of migrants, some lineages probably disappeared or were replaced due to the many conflicts and revolts of these groups between the 17^th^ and the 20^th^ centuries [60]. The Anteony are genetically highly differentiated from all other Malagasy groups. Analysis of haplogroup diversity allowed us to understand these differences between the former Antemoro aristocrat caste and the other Malagasy groups. The Anteony differed because of the presence in very high frequency of both haplogroups J1 and T1 (note that the SNP which defined haplogroup J1 was not tested in the Highlands).

The Antalaotra showed the highest genetic diversity, the result of numerous migrations creating a larger population, thereby reducing the phenomena of genetic drift and selection [60]. The Antalaotra is a heterogeneous group as it includes many other subdivisions according to the literature and the participants. These subgroups seem to have different geographical origins. Moreover, the Antalaotra were extensive travelers, who were known to transmit knowledge and charms in Madagascar [61]. Compare to other Malagasy groups, the Antalaotra show little differentiation from the Malagasy Highlands population (Table S8 in File S1). However, for the Highlands population, data on Y-STR were not available and therefore analysis of shared haplotypes could not be made. We could only note that for the few available profiles from the Merina in this region, no haplotypes were shared based on the 17 markers. Contact between the Highlands and especially the Merina has been described, for example, in the beginning of the 19^th^ century [62]. The very high proportion of Asian lineages in the Antalaotra group may correspond to extensive admixture with the ‘Malagasy’ genetic diversity already in the area when the Antemoro migration occurred. A second hypothesis would be that their origin is Islamized Island Southeast Asia, a population carrying Middle East lineages and a main Southeast Asian genetic background. Nevertheless, even if sampling bias cannot be excluded, comparative analysis with our database preferentially linked J1 and T1 haplogroups to a direct Southwestern origin. We also noted that some haplotypes in the background of haplogroups E1b1a, T1 and J1 were shared between the Anteony and Antalaotra, highlighting similar origins or relatively recent interbreeding. If we consider ethnological studies, the Antemoro practiced strict endogamy until recently [12], so genetic diversity would be reduced by the effect of drift, which is not the case in the Antalaotra. The genetic diversity found in the Antalaotra should have been brought with the Antemoro migration.

The Ampanabaka did not share any haplotypes, based on 17 Y-STR markers, with the other two Antemoro groups. The admixture computation confirmed that the Ampanabaka had a genetic diversity close to that of the Bantu, and also contributions from southeastern Asia; this can be shown just by observing the haplogroup compositions. Compared with other populations from Madagascar available in the literature, the Ampanabaka showed a ‘Malagasy’ genetic diversity and presented little differentiation from the Antanosy and southern Vezo. We should note that the Antanosy, according to oral tradition, were descendants of Islamized individuals, called the ZafiRaminia, from the previous migration. Moreover, contact between groups from the Menabe, the Sakalava on the west coast of Madagascar and the Antemoro have been highlighted by some authors [63]. The Ampanabaka shared a haplotype (17 Y-STR) with an Antanosy (E2b) and an Antandroy (E1b1a).The haplogroup diversity of the Ampanabaka agrees with that generally found in Madagascar: a high proportion of E1b1a associated with an important presence of haplogroup O1a2. These individuals could have been ‘Malagasy groups’ migrating to place under the authority of the Antemoro kingdom. 

Haplogroup J1 is predominant in Middle East, and also very frequent in North and Northeast Africa, Europe, India and Pakistan [42][64][65]. The T1 haplogroup, found at very low frequency in the world, seems to have a Southwestern Asia origin and to be associated with many demographic processes such as the spread of agriculture, the Assyrian and Babylonian exiles and the Jewish Diaspora [49]. Its presence in eastern Asia could be due to commercial and cultural exchanges via the former Silk Road [50]. Currently, it is found mainly in the Middle East but also in eastern Africa, northern Africa and probably in other regions that have been in contact with these geographic areas. However, the J1 Median-Joining network on the minimal haplotype showed that the J1 haplotype in the Antemoro corresponded exactly to those found in individuals from Cyprus, Turkey and Palestine (Figure 6) and was close to some haplotypes from the Comoros. The study by Msaidie (2010) [43] on Comoros populations showed the presence of haplogroups J1 in this area and the author deduced from Comoros populations haplogroup frequencies analysis that the gene flow would be compatible with an origin from Iran. We can thus assume that these haplotypes appear to have a common genetic origin. The Median-Joining network for T1 haplotypes links these lineages to Israel, Lebanon and Palestine. These results are also consistent with the low F_ST_ values between Anteony and Antalaotra and populations from Middle East/Southwest Asia. The combination of these two lineages (J1 and T1) tends to converge to an origin in the Persian Gulf or Middle East. 

### Comparison of mtDNA and historic data

Analysis of maternal lineages revealed haplogroups usually found in Madagascar. The vast majority of HVI haplotypes were shared with at least one Malagasy population from the south and the Highlands. This result reflects the existence of many intra-population movements and extensive interbreeding in Madagascar. Genetic diversity was very homogeneous and suggests important recent arrivals; the ‘Polynesian motif’ (especially the ‘Malagasy’ motif) was found among others, reflecting the migration of Austronesian Malays most especially in the Antalaotra group. The genetic diversity of the Antemoro most especially for the Anteony, was also very close to African populations (Kenya and Senegal; Table S15 in File S1).

The analysis of the maternal gene pool revealed the absence of haplogroups typically found in the Middle East. This result agrees with the Antemoro tradition of migrant men finding a wife on the Great Island or that the wives were “transported” from Eastern African coast. Also during these centuries, men dominated trade and campaigns of religious conversions. The maternal heritage of the three Antemoro groups was not significantly differentiated from other Malagasy populations.

## Conclusions

If we assume that early migration across the Indian Ocean involved regions from ISEA, the African eastern coast and Southwestern Asia (Middle East) [1,4], the fact that only two genetic Y-haplogroups in the Antemoro population have Middle Eastern genetic origin can lead to three hypotheses: (1) A sampling bias: more samples from Antemoro groups enable the discovery of other representative haplogroups of this geographic region. (2) Those haplogroups were introduced in Madagascar before the Antemoro settlement. So the migration would come from East African or Southeast Asian regions in which these haplogroups were already diluted in an African or Southeast Asian genetic diversity. In that case a bias in the database we used to perform our comparisons is possible. Or (3) there was a founder effect in some Middle Eastern male lineages, and only some are currently still present. 

This study has highlighted a Middle Eastern biological trace in Madagascar consistent with the Middle Eastern cultural tradition of the population involved. The results of the Antemoro gene pool analysis suggest a Middle Eastern origin to some of the Y chromosome variation associated specifically with haplogroups J1 and T1, but this does not exclude an origin of this variation from unsampled/not studied African or Southeastern Asian populations. However, there is no Middle Eastern origin for the mtDNA gene pool. Nevertheless, this link is based on very few haplogroups, and the ethnogenesis and origin of all Antemoro groups, as well as the genetic impact of Middle Eastern populations around the Indian Ocean fringe during the last millennium remain open to discussion. Although, genetic analyses do not prove or refute a single theory concerning the geographical origin of the Antemoro, this study has suggested a Middle Eastern biological trace in the southeast coast of Madagascar. A study of the genetic contribution of Malagasy people from the two previous migrations to the coast (Onjatsy and ZafiRaminia) would be necessary. The groups in the north of the island where Arab trading posts have been described could also be of particular interest. The Antemoro migration would have arrived in the north in Vohemar before moving along the Malagasy coast to the south. Moreover, more data from archipelagos like the Kilwa islands would allow us to possibly relate the Antemoro migrations to the history of these areas. Gathering information on population movement from Middle East/Southwest Asia around the Indian Ocean would also be necessary. Futures objectives for studies on Malagasy populations will be to work at a genome wide scale in order to infer more accurate proportions and dates of admixture. 

## Supporting Information

File S1 **Supplementary tables and figures**.
**Figure S1, MDS plot of FST between Malagasy populations and the Comoros using NRY haplogroup frequencies (Kruskal stress: 0.172)**. **Figure S2, MDS plot of FST computed from Y haplogroup frequencies between the Antemoro and populations from various geographic regions (Kruskal stress =0.227)**. **Figure S3, MDS plot computed from FST values between Malagasy populations, based on HVI data**. **Figure S4, PCA computed from mitochondrial haplogroups frequency in Malagasy and Comoros populations**. **Figure S5, MDS plots of FST computed from HVI haplotypes between the Antemoro and populations from various geographic regions**. **Table S1, Y-STR profiles in the Antemoro**. **Table S2, Database used for the analysis of Y haplogroup frequencies**. **Table S3, Database used for the analysis of the seven Y-STR markers**. **Table S4, J1 haplotype references used for the median-joining network**. **Table S5, T1 haplotype references used for the median-joining network**. **Table S6, HVI and HVII profiles in the Antemoro**. **Table S7, Database used for HVI analysis**. **Table S8, Table of population pairwise FST values based on Y haplogroup frequencies**. **Table S9, Shared haplotypes between the Ampanabaka, other Malagasy populations and the Comoros, using 17 Y-STR markers**. **Table S10, Shared haplotypes between the Anteony, other Malagasy populations and the Comoros, using 17 Y-STR markers**. **Table S11, Shared haplotypes between the Antalaotra, other Malagasy populations and the Comoros, using 17 Y-STR markers**. **Table S12, Shared haplotypes between the Ampanabaka and the database using seven YSTR markers**. **Table S13, Shared haplotypes between the Antalaotra and the database, using seven Y-STR markers**. **Table S14, Shared haplotypes between the Anteony and the database using seven Y-STR markers**. **Table S15, Population pairwise FST values based on HVI in our three Antemoro groups**. **Table S16, Shared HVI haplotypes between the Ampanabaka and the other Malagasy populations**. **Table S17, Shared HVI haplotypes between the Antalaotra and other Malagasy populations**. **Table S18, Shared HVI haplotypes between the Anteony and other Malagasy populations**. **Table S19, Shared HVI unique haplotypes between the Ampanabaka and populations from the database**. **Table S20, Shared HVI unique haplotypes between the Antalaotra and populations from the database**. **Table S21, Shared HVI unique haplotypes between the Anteony and populations from the database**.
(PDF)
