# Supplementary material for: Tracing Arab-Islamic Inheritance in Madagascar: Study of the Y-chromosome and Mitochondrial DNA in the Antemoro
Source: PLoS One. 2013 Nov 22;8(11):e80932. doi: 10.1371/journal.pone.0080932 (PMC3838347; doi:10.1371/journal.pone.0080932)
Supplement: File S1 — Supplementary tables and figures. Figure S1, MDS plot of FST between Malagasy populations and the Comoros using NRY haplogroup frequencies (Kruskal stress: 0.172). Figure S2, MDS plot of FST computed from Y haplogroup frequencies between the Antemoro and populations from various geographic regions (Kruskal stress =0.227). Figure S3, MDS plot computed from FST values between Malagasy populations, based on HVI data. Figure S4, PCA computed from mitochondrial haplogroups frequency in Malagasy and Comoros populations. Figure S5, MDS plots of FST computed from HVI haplotypes between the Antemoro and populations from various geographic regions. Table S1, Y-STR profiles in the Antemoro. Table S2, Database used for the analysis of Y haplogroup frequencies. Table S3, Database used for the analysis of the seven Y-STR markers. Table S4, J1 haplotype references used for the median-joining network. Table S5, T1 haplotype references used for the median-joining network. Table S6, HVI and HVII profiles in the Antemoro. Table S7, Database used for HVI analysis. Table S8, Table of population pairwise FST values based on Y haplogroup frequencies. Table S9, Shared haplotypes between the Ampanabaka, other Malagasy populations and the Comoros, using 17 Y-STR markers. Table S10, Shared haplotypes between the Anteony, other Malagasy populations and the Comoros, using 17 Y-STR markers. Table S11, Shared haplotypes between the Antalaotra, other Malagasy populations and the Comoros, using 17 Y-STR markers. Table S12, Shared haplotypes between the Ampanabaka and the database using seven YSTR markers. Table S13, Shared haplotypes between the Antalaotra and the database, using seven Y-STR markers. Table S14, Shared haplotypes between the Anteony and the database using seven Y-STR markers. Table S15, Population pairwise FST values based on HVI in our three Antemoro groups. Table S16, Shared HVI haplotypes between the Ampanabaka and the other Malagasy populations. Table S17, Shared HVI haplotypes [file pone.0080932.s001.pdf]

Supporting information - Tracing Arab-Islamic inheritance in Madagascar: study of the Y-chromosome and mitochondrial DNA in the Antemoro.

Mélanie Capredon[1,2,3], Nicolas Brucato[4], Laure Tonasso[1], Valérie Choesmel-Cadamuro[1], François-Xavier Ricaut[1], Harilanto Razafindrazaka[1], Andriamihaja Bakomalala Rakotondrabe[5], Mamisoa Adelta Ratolojanahary [5], Louis-Paul Randriamarolaza[5], Bernard Champion [2], Jean-Michel Dugoujon[1]

[1]Laboratoire d'Anthropologie Moléculaire et Imagerie de Synthèse, CNRS and Université Paul Sabatier Toulouse III, UMR5288, Toulouse, France; [2] Centre de recherche littéraire et historique de l'Océan Indien (CRLHOI), Département d'ethnologie, Université de La Réunion, Saint-Denis, France ; [3] Department of Pediatrics, CHU Sainte Justine, Faculty of Medecine, University of Montreal, Quebec, Canada ; [4] Language and Genetics Department, Max Planck Institute for Psycholinguistics, Nijmegen, The Netherlands. ; [5] Laboratoire d'Anthropologie Patrimoine -Transformations sociales- Transculturalité (LAP2T), Université Antananarivo, Antananarivo, Madagascar.

Table S1. Y-STR profiles in the Antemoro **3**

Table S2. Database used for the analysis of Y haplogroup frequencies **8**

Table S3. Database used for the analysis of the seven Y-STR markers **11**

Table S4. J1 haplotype references used for the median-joining network **13**

Table S5. T1 haplotype references used for the median-joining network **14**

Table S6. HVI and HVII profiles in the Antemoro **16**

Table S7. Database used for HVI analysis **21**

Table S8. Table of population pairwise F_{ST} values based on Y haplogroup frequencies. ... **24**

Table S9. Shared haplotypes between the Ampanabaka, other Malagasy populations and the Comoros, using 17 Y-STR markers **28**

Table S10. Shared haplotypes between the Anteony, other Malagasy populations and the Comoros, using 17 Y-STR markers **29**

Table S11. Shared haplotypes between the Antalaotra, other Malagasy populations and the Comoros, using 17 Y-STR markers. **30**

Figure S1. MDS plot of F_{ST} between Malagasy populations and the Comoros using NRY haplogroup frequencies (Kruskal stress: 0.172). **32**

Table S12. Shared haplotypes between the Ampanabaka and the database using seven Y-STR markers **33**

Table S13. Shared haplotypes between the Antalaotra and the database, using seven Y-STR markers **36**

Table S14. Shared haplotypes between the Anteony and the database using seven Y-STR markers **39**

Figure S2. MDS plot of F_{ST} computed from Y haplogroup frequencies between the Antemoro and populations from various geographic regions (Kruskal stress =0.227). Anty: Anteony, Anta: Antalaotra, Ampa: Ampanabaka. 43

Table S15. Population pairwise F_{ST} values based on HVI in our three Antemoro groups . 44

Figure S3. MDS plot computed from F_{ST} values between Malagasy populations, based on HVI data. Lower diagonal: F_{ST}; upper diagonal: p-values. (+) p-value significant at 1% ; (-) p-values non- significant at 1%. Kruskal stress = 0.329. 47

Figure S4. PCA computed from mitochondrial haplogroups frequency in Malagasy and Comoros populations. The insert showed the contribution of haplogroups to the two components. 48

Table S16. Shared HVI haplotypes between the Ampanabaka and the other Malagasy populations. 49

Table S17. Shared HVI haplotypes between the Antalaotra and other Malagasy populations 49

Table S18. Shared HVI haplotypes between the Anteony and other Malagasy populations. 50

Table S19. Shared HVI unique haplotypes between the Ampanabaka and populations from the database 51

Table S20. Shared HVI unique haplotypes between the Antalaotra and populations from the database 55

Table S21. Shared HVI unique haplotypes between the Anteony and populations from the database 59

Figure S5. MDS plots of F_{ST} computed from HVI haplotypes between the Antemoro and populations from various geographic regions. (a) African populations, (b) Southeast Asian and Oceanian populations; (c) European and west Eurasian populations. 64

References 65

Table S1. Y-STR profiles in the Antemoro

Groupe	ID (Hg)	DYS393	DYS390	DYS 19	DYS 391	DYS385ab	DYS 439	DYS 389I	DYS 392	DYS 389II	DYS 458	DYS 437	DYS 448	YGATAH4	DYS 456	DYS 438	DYS 635
**Ampanabaka**	**MA301 (E1b1a)**	13	21	15	10	17,18	11	13	11	31	15	14	21	12	15	11	21
	**MA302 (E1b1a)**	13	21	15	10	16,18	11	13	11	31	15	14	21	12	15	11	21
	**MA303 (E1b1a)**	13	21	15	10	16,18	11	13	11	31	15	14	21	12	15	11	21
	**MA304 (E1b1a)**	14	21	17	10	17,19	12	13	11	30	17	13	21	11	15	11	21
	**MA306 (E1b1a)**	13	21	15	10	17,18	12	13	11	31	15	14	21	13	15	11	21
	**MA307 (O1a2)**	13	23	15	10	13,14	11	12	14	28	17	14	16	11	15	10	22
	**MA309 (E1b1a)**	13	21	15	10	17,18	11	13	11	31	15	14	21	12	15	11	21
	**MA310 (E1b1a)**	13	21	15	10	16,18	11	13	11	31	15	14	21	12	15	11	21
	**MA311 (E1b1a)**	13	21	15	11	16,18	11	13	11	30	16	14	21	12	16	11	21
	**MA312 (E1b1a)**	13	21	15	10	16,18	11	13	11	31	15	14	21	12	15	11	21
	**MA313 (E1b1a)**	13	21	15	10	17,17	13	13	11	31	16	14	21	13	15	11	22
	**MA314 (E1b1a)**	13	21	15	10	17,18	11	13	11	31	15	14	21	12	15	11	21
	**MA315 (E1b1a)**	13	21	15	10	17,18	11	13	11	31	15	14	21	12	15	11	21
	**MA316 (E1b1a)**	13	21	15	10	16,2	12	13	11	31	18	14	21	13	15	11	22
	**MA319 (E1b1a)**	13	22	15	10	16,17	12	14	12	33	17	14	21	10	15	11	21
	**MA321 (E1b1a)**	13	21	15	10	14,18	12	14	11	32	15	14	20	11	15	11	21
	**MA322 (E1b1a)**	13	21	15	11	16,17	11	13	11	31	16	14	21	12	15	11	21
	**MA323 (O1a2)**	14	23	15	10	13,14	11	12	14	28	18	14	16	12	16	10	21
	**MA325 (E1b1a)**	14	21	17	11	15,19	12	13	11	31	17	13	21	11	15	11	21
	**MA326 (E1b1a)**	14	21	15	11	15,2	13	13	11	30	16	14	21	11	15	11	23
	**MA327 (E1b1a)**	13	21	15	10	16,18	11	13	11	30	15	14	21	12	15	11	21
	**MA328 (O1a2)**	13	23	16	10	14,15	11	12	14	29	19	14	16	12	15	10	22
	**MA329 (E1b1a)**	13	21	15	10	16,18	11	13	11	31	15	14	21	12	15	11	21
	**MA330 (O1a2)**	13	23	16	10	13,14	11	12	14	28	18	14	16	12	15	10	21
	**MA332 (E1b1a)**	13	21	15	11	16,17	11	13	11	31	16	14	21	12	15	11	22

	**MA333 (E1b1a)**	13	21	15	10	17,18	11	13	11	31	15	14	22	12	15	11	21
	**MA334 (E1b1a)**	14	21	15	10	16,18	12	13	11	31	17	14	21	12	16	11	21
	**MA335 (E1b1a)**	13	21	15	10	17,18	11	13	11	31	15	14	21	12	15	11	21
	**MA337 (O1a2)**	13	23	16	10	14,15	11	13	13	29	21	14	16	11	15	10	22
	**MA338 (E1b1a)**	13	21	15	10	17,18	11	13	11	31	15	14	21	12	15	11	22
	**MA339 (O1a2)**	13	23	15	10	13,14	11	12	14	28	17	14	16	11	15	10	22
	**MA342 (B2a)**	13	24	15	10	11,11	11	14	11	33	17	14	22	12	13	10	17
	**MA343 (E1b1a)**	14	21	15	11	15,2	13	13	11	30	16	14	21	12	15	11	23
	**MA344 (E1b1a)**	14	21	16	10	17,19	12	13	11	30	16	13	19	11	15	11	21
	**MA345 (O1a2)**	13	22	15	10	13,15	12	12	14	28	18	14	16	11	15	10	22
	**MA346 (E1b1a)**	15	21	15	11	16,16	13	13	11	30	17	14	21	11	15	11	22
	**MA347 (E1b1a)**	13	21	15	10	17,17	11	13	**11**	31	15	14	21	12	15	11	21
	**MA348 (E1b1a)**	13	21	15	10	17,18	11	13	11	31	15	14	21	12	14	12	21
	**MA350 (E1b1a)**	13	21	15	10	18,18	11	13	11	30	15	14	21	12	15	11	21
	**MA351 (E2b)**	13	24	14	11	14,18	11	12	11	28	17	14	19	11	15	11	25
	**MA352 (J2b)**	13	24	15	11	13,17	12	12	11	29	16	15	18	11	13	9	21
	**MA354 (E1b1a)**	14	21	14	11	16,16	12	13	11	30	18	14	21	11	16	11	21
	**MA355 (E1b1a)**	13	21	15	10	17,18	11	13	11	31	15	14	21	12	15	11	21
	**MA356 (E1b1a)**	13	21	15	10	16,16	12	14	11	32	16	14	21	12	15	11	22
	**MA357 (B(xB2a))**	13	23	15	11	12,12	11	11	11	26	15	14	19	12	15	10	19
	**MA358 (E1b1b1)**	13	24	13	10	16,18	12	14	11	31	16	14	20	12	17	10	21
**Anteony**	**MA359 (J1(xJ1a,J1b))**	12	23	15	10	13,18	11	13	11	29	18*	14	20	11	15	10	20
	**MA360 (T1)**	13	23	15	10	14,16	11	13	13	30	16	14	19	12	15	9	22
	**MA361 (J1(xJ1a,J1b))**	12	23	15	10	13,18	13	13	11	29	18*	14	20	11	15	10	20
	**MA362 (J1(xJ1a,J1b))**	12	23	15	10	13,18	11	13	11	29	18*	14	20	11	15	10	20
	**MA363 (J1(xJ1a,J1b))**	12	23	15	10	13,18	11	13	11	29	18*	14	20	11	15	10	20
	**MA364 (J1(xJ1a,J1b))**	12	23	15	10	13,18	11	13	11	29	18*	14	20	11	15	10	20

**MA365 (T1)**	13	23	15	10	14,16	11	13	13	30	16	14	19	12	15	9	22
**MA366 (T1)**	13	23	15	10	14,16	11	13	13	30	16	14	19	12	15	9	22
**MA369 (J1(xJ1a,J1b))**	12	23	15	10	13,18	11	13	11	29	18*	14	20	11	15	10	20
**MA370 (J1(xJ1a,J1b))**	12	23	15	10	13,18	11	13	11	29	18*	14	20	11	15	10	20
**MA373 (T1)**	13	23	15	10	14,16	11	13	13	30	16	14	19	12	15	9	22
**MA376 (J1(xJ1a,J1b))**	12	23	15	10	13,18	11	13	11	29	18*	14	20	11	15	10	20
**MA377 (T1)**	13	23	15	10	14,16	11	13	13	30	16	14	19	12	15	9	22
**MA378 (T1)**	13	23	15	10	14,16	11	13	13	30	16	14	19	12	15	9	21
**MA379 (T1)**	13	23	15	10	14,16	11	14	13	31	16	14	19	12	15	9	22
**MA380 (T1)**	13	23	15	10	15,16	11	14	13	31	16	14	19	12	15	9	23
**MA381 (T1)**	13	23	15	10	14,16	11	13	13	30	16	14	19	12	14	9	21
**MA382 (E1b1a)**	14	21	17	10	15,16	12	13	11	30	16	14	21	11	17	11	23
**MA383 (T1)**	13	23	15	10	14,16	11	14	13	31	16	14	19	12	15	9	22
**MA384 (J1(xJ1a,J1b))**	12	23	15	10	13,18	11	13	11	29	18*	14	20	11	15	10	20
**MA385a (E1b1a)**	14	21	17	10	15,17	12	13	11	30	16	14	21	11	16	11	23
**MA387 (T1)**	13	23	15	10	14,16	11	14	13	31	16	14	19	12	15	9	22
**MA388 (T1)**	13	23	16	10	14,15	12	14	13	31	16	14	19	12	15	9	22
**MA390 (T1)**	13	23	15	10	14,16	11	13	13	30	16	14	19	12	15	9	22
**MA391 (E1b1b1)**	13	24	13	10	15,19	11	13	11	30	16	14	20	12	17	10	23
**MA392 (T1)**	13	23	15	10	14,16	11	13	13	29	16	14	19	12	15	9	22
**MA393 (T1)**	13	23	15	10	14,16	11	13	13	30	16	14	19	12	15	9	22
**MA395 (T1)**	13	23	15	10	14,16	12	13	13	30	16	14	19	12	15	9	22
**MA396 (E1b1a)**	13	22	15	10	16,18	12	14	12	33	17	14	21	10	15	11	21
**MA397 (O2a1)**	14	25	15	11	20,21	12	13	13	29	16	14	18	11	16	10	25
**MA399 (T1)**	13	23	15	10	14,16	11	13	13	30	16	14	19	12	15	9	22
**MA400 (T1)**	13	23	15	10	14,16	11	13	13	30	16	14	19	12	15	9	21
**MA404 (T1)**	13	23	15	10	14,16	11	14	13	31	16	14	19	12	15	9	22

	**MA405 (T1)**	13	24	15	10	14,16	11	14	13	31	16	14	19	12	15	9	22
	**MA407 (E1b1a)**	13	21	15	10	17,18	11	13	11	31	15	14	21	11	15	11	21
	**MA408 (O2a1)**	14	25	15	11	15,2	12	13	13	29	15	14	18	11	14	10	22
	**MA409 (T1)**	13	23	16	10	14,16	11	14	13	31	16	14	19	12	15	9	22
	**MA410 (T1)**	13	23	15	10	14,16	11	14	13	31	16	14	19	12	15	9	22
	**MA411 (J1(xJ1a,J1b))**	12	23	15	10	13,18	11	13	11	29	18*	14	20	11	15	10	21
	**MA412 (E1b1a)**	13	21	15	11	16,17	11	13	11	31	18	14	21	11	15	11	21
**Antalaotra**	**MA416 (O2a1)**	13	25	15	11	16,21	12	13	13	29	16	14	18	11	16	10	25
	**MA417 (O1a2)**	13	23	16	10	13,13	11	12	14	28	15	14	16	12	15	10	22
	**MA418 (E1b1a)**	14	21	17	10	18,18	13	13	11	30	17	14	21	11	15	11	21
	**MA419 (O2a1)**	13	25	14	12	16,21	12	13	13	29	16	14	18	11	16	10	25
	**MA420 (O2a1)**	14	25	15	11	16,23	12	13	13	29	16	14	18	11	16	10	25
	**MA421 (J2b)**	13	24	15	10	13,17	11	12	11	29	16	15	18	11	13	9	21
	**MA422 (E2b)**	13	25	14	11	13,19	11	12	11	28	16	14	19	11	15	11	25
	**MA423 (E2b)**	13	25	14	11	13,19	11	12	11	28	17	14	19	11	15	11	25
	**MA424 (J2b)**	13	24	15	11	13,18	12	12	11	29	16	15	18	11	13	9	21
	**MA425 (E1b1a)**	13	21	15	11	16,17	11	13	11	31	18	14	21	11	15	11	21
	**MA426 (E2b)**	13	25	14	11	13,19	11	12	11	28	17	14	19	11	15	11	25
	**MA429 (E2b)**	13	25	14	12	13,19	11	12	11	28	17	14	19	11	14	11	26
	**MA430 (E2b)**	13	25	14	11	13,19	11	12	11	28	17	14	19	11	15	11	24
	**MA431 (J1(xJ1a,J1b))**	12	23	15	10	13,18	11	13	11	29	18*	14	20	11	15	10	20
	**MA433 (J2b)**	13	24	15	11	13,18	12	12	11	30	16	15	18	11	13	9	21
	**MA435 (J1(xJ1a,J1b))**	12	23	15	10	13,19	11	13	11	29	18*	14	20	11	15	10	20
	**MA436 (O2a1)**	14	24	15	11	16,21	12	13	13	29	16	14	18	11	16	10	25
	**MA437 (O1a2)**	13	23	16	10	15,15	11	12	14	28	19	14	16	12	16	10	22
	**MA438 (J1(xJ1a,J1b))**	12	23	15	10	13,19	11	13	11	29	18*	14	20	11	15	10	20
	**MA439 (E2b)**	13	25	14	11	13,19	11	12	11	28	17	14	19	11	15	11	25

**MA440 (O2a1)**	13	25	15	11	16,21	12	13	13	29	17	14	18	11	16	10	25
**MA441 (O2a1)**	14	25	16	11	16,21	12	13	13	29	16	14	18	11	16	10	25
**MA442 (O2a1)**	14	24	15	11	16,21	12	13	13	29	16	14	18	11	16	10	25
**MA443 (O2a1)**	14	25	15	11	16,21	12	13	13	29	16	14	18	11	16	10	25
**MA444 (O1a2)**	13	23	15	10	13,14	11	12	14	28	19	14	16	12	16	10	21
**MA446 (T1)**	13	23	15	10	14,16	11	13	13	30	17	14	19	12	15	9	22
**MA447 (O1a2)**	13	23	16	10	13,13	11	12	14	28	15	14	16	12	16	10	22
**MA448 (O1a2)**	13	23	16	10	13,13	11	12	14	28	15	14	16	12	16	10	21
**MA450 (J1(xJ1a,J1b))**	12	23	15	10	13,18	11	13	11	29	18*	14	20	11	15	10	20
**MA451 (O1a2)**	13	23	17	10	13,14	11	12	14	30	19	14	16	12	16	10	22
**MA452 (O2a1)**	13	23	16	10	13,14	11	12	14	29	19	14	16	12	16	10	21
**MA453 (O2a1)**	13	25	15	11	16,21	12	13	13	29	16	14	18	11	16	10	24
**MA454 (R1a)**	13	25	16	11	12,13	10	13	11	30	16	14	19	13	15	11	23
**MA455 (O2a1)**	13	25	15	11	16,2	12	13	13	29	16	14	18	11	16	10	25
**MA456 (O1a2)**	13	23	15	10	13,13	11	12	14	28	15	14	16	12	16	10	21
**MA458 (T1)**	13	23	15	10	14,16	11	13	13	30	17	14	19	12	15	9	22
**MA459 (J1(xJ1a,J1b))**	12	23	15	10	11,19	11	13	11	29	18*	14	20	11	15	10	20
**MA460 (T1)**	13	23	15	10	14,16	11	14	13	31	16	14	19	12	15	9	21
**MA462 (J1(xJ1a,J1b))**	12	23	15	10	13,18	11	13	11	29	18*	14	20	11	15	10	20
**MA463 (O2a1)**	14	25	15	11	16,21	12	13	13	29	16	14	18	11	17	10	23
**MA465 (J1(xJ1a,J1b))**	12	23	15	10	13,18	11	13	11	29	18*	14	20	11	15	10	20
**MA468 (T1)**	13	23	15	10	14,16	11	14	13	31	16	14	19	12	15	9	22
**MA469 (E1b1a)**	14	21	15	10	15,19	14	13	11	30	16	14	21	12	15	11	21

*DYS438 :18/19 variants not tested

Table S2. Database used for the analysis of Y haplogroup frequencies

Geographic group	Population	n	References
**Africa CWS**	Angola Cabinda	74	[1]
**Africa CWS**	Angola Nyaneka-Nkumbi	75	[2]
**Africa CWS**	Angola Ovimbudu	96	[2]
**Africa CWS**	DRC (Pygmées Mbuti)	99	[3]
**Africa CWS**	South Africa!Kung	100	[3]
**Africa CWS**	South AfricaKhwe	101	[3]
**Africa CWS**	Rwanda Hutu	98	[4]
**Africa CWS**	Rwanda Tutsi	100	[4]
**Africa CWS**	Eastern Zambia	69	[5]
**Africa CWS**	Western Zambia Bantu west	197	[5]
**Africa CWS**	Western Zambia Bantu east	283	[5]
**Africa CWS**	Equatorial Guinea(estimation)	101	[6]
**Africa CWS**	Benin	78	[7]
**Africa CWS**	Burkina Faso Fulbe	100	[3]
**Africa CWS**	Burkina Faso Mossi	99	[3]
**Africa CWS**	Burkina Faso Rimaibe	99	[3]
**Africa CWS**	Cameroon Bakola	100	[3]
**Africa CWS**	Cameroon Bamileke	100	[3]
**Africa CWS**	Cameroon Daba	100	[3]
**Africa CWS**	Cameroon Ewondo	100	[3]
**Africa CWS**	Cameroon Fali	100	[3]
**Africa CWS**	Cameroon Fulbe	101	[3]
**Africa CWS**	Cameroon various Adamawa	102	[3]
**Africa CWS**	Cameroon various Chadic	102	[3]
**Africa CWS**	Cameroon Nilo-Sahara	99	[3]
**Africa CWS**	Cameroon Tali	101	[3]
**Africa CWS**	Cameroon Uldeme	100	[3]
**Africa CWS**	Central Africa Republic Lissongo	140	[3]
**Africa CWS**	Cabo Verde Northern	101	[8]
**Africa CWS**	Cabo Verde Southern	100	[8]
**Africa CWS**	Benin Fon	100	[4]
**Africa CWS**	Cameroon Bantu	99	[4]
**Africa CWS**	Senegal various	139	[9]
**Eastern Africa**	Tanzania Datoga	101	[10]
**Eastern Africa**	Tanzania Hadzabe	99	[10]
**Eastern Africa**	Kenya Bantu	81	[4]
**Eastern Africa**	Tanzania Wairak	100	[4]
**Eastern Africa**	Somalia various Danish	196	[11]
**Eastern Africa**	Kenya	79	[12]
**Eastern Africa**	Karamoja, Uganda	118	[13]
**Eastern Africa**	Kenya Maasai	79	[5]
**Eastern Africa**	Ethiopia Jew	101	[3]

**Eastern Africa**	Ethiopia Amhara	48	[9]
**Eastern Africa**	Ethiopia Oromo	78	[9]
**Eastern Africa**	Ethiopia Omo valley	98	[5]
**Eastern Africa**	Somalia	201	*[14]*
**Northern Africa**	Namibia	136	[12]
**Northern Africa**	Egypt	147	*[14]*
**Northern Africa**	Algeria various	100	[15]
**Northern Africa**	Lybia Arabs (estimation)	51	[16]
**Northern Africa**	Morocco Arabs	100	[3]
**Northern Africa**	Morocco Berbers	101	[3]
**Northern Africa**	Morocco	312	[17]
**Northern Africa**	Tunisia	148	[18]
**Southern Asia**	Pakistan	176	*[14]*
**Southern Asia**	Naikpod	68	*[19]*
**Southern Asia**	Andh	53	*[19]*
**Southern Asia**	Pardhan	128	*[19]*
**Southern Asia**	Mahadeokoli	50	[20]
**Southern Asia**	Thakar	48	[20]
**Southern Asia**	Muslim Shia	161	[21]
**Southern Asia**	Muslim Sunni	129	[21]
**Southern Asia**	Northern India	80	*[22]*
**Southern Asia**	NorthesternIndia	87	*[22]*
**Southern Asia**	Eastern India	128	*[22]*
**Southern Asia**	Southern India	303	*[22]*
**Southern Asia**	Central India	71	*[22]*
**Southern Asia**	Western India	59	*[22]*
**Western Europe**	Northern Portugal	60	[23]
**Western Europe**	Southern Portugal	78	[23]
**Western Europe**	Portugueses Jew	86	[24]
**Western Europe**	France	57	(Balaresque, unpublished)
**Western Europe**	Basque	116	[23]
**Western Europe**	East Andalusia	95	[23]
**Western Europe**	Galicia	88	[23]
**Western Europe**	Northwestern Castilla	100	[23]
**West Indian Ocean islands**	Antandroy	46	[25]
**West Indian Ocean islands**	Antanosy	47	[25]
**West Indian Ocean islands**	Comoros	381	[26]
**West Indian Ocean islands**	Mikea Mikea-vezo	59	[27]
**West Indian Ocean islands**	Vezo Northern Tulear	16	[27]
**West Indian Ocean islands**	Vezo Southern Tulear	32	[27]
**West Indian Ocean islands**	HighlandsMadagascar	35	[28]

**Middle East**	Israel Druzes Galilee	173	[29]
**Middle East**	Palestinian	290	[17]
**Middle East**	Koweït Bedouins	148	[30]
**Middle East**	Soqotra various	63	[31]
**Middle East**	Saudi Arabia	157	*[14]*
**Middle East**	Qatar	72	*[14]*
**Middle East**	UAE	164	*[32]*
**Middle East**	Oman	121	*[32]*
**Middle East**	Yemen	62	*[32]*
**Middle East**	Liban	916	[33]
**Middle East**	Jordan	146	[34]
**Middle East**	Irak	203	[35] [36]
**Middle East**	Iran	150	[37]
**Middle East**	Syrians	518	[17] [38]
**Middle East**	Iranians	324	[39]
**Middle East**	Cypriots	165	[38]
**Middle East**	Turks various	58	[35]
**Middle East**	Turks Anatolie	488	[40]
**Oceania**	Vanuatu-Maewo	44	[41]
**Oceania**	South western New Guinea Una	46	[42]
**Oceania**	Papouasie New Guinea Trobriand	53	[42]
**Oceania**	Papouasie New Guinea Kapuna	46	[42]
**Oceania**	Cook	66	[42]
**Oceania**	Futuna	50	[42]
**Oceania**	Fidji	94	[42]
**Oceania**	Tuvalu	100	[42]
**Oceania**	Western Samoa	61	[42]
**Southern Asia**	Nias	407	[43]
**Southern Asia**	Philippines	48	[41]
**Southern Asia**	Vietnam	70	[41]
**Southern Asia**	Bali	641	[41]
**Southern Asia**	Java	61	[41]
**Southern Asia**	Bornéo	86	[41]
**Southern Asia**	Nias	60	[41]
**Southern Asia**	Mentawai	74	[41]
**Southern Asia**	Flores	394	[41]
**Southern Asia**	Sulawesi	54	[41]
**Southern Asia**	Sumba	350	[41]
**Southern Asia**	Lembata	92	[41]
**Southern Asia**	Adonara (Austronesian language)	96	[44]
**Southern Asia**	Flores (Austronesian language)	71	[44]
**Southern Asia**	Solor (Austronesian language)	43	[44]
**Southern Asia**	Sumatra	56	[42]
**Southern Asia**	Java	53	[42]
**Southern Asia**	Negritos	180	[45]

**Southern Asia**	Non Negritos	210	[42]
**Southern Asia**	Malaysia	32	[41]

Table S3. Database used for the analysis of the seven Y-STR markers

Geographic group	Population	n	References
**West Indian Ocean islands**	Antandroy	46	[25]
**West Indian Ocean islands**	Antanosy	47	[25]
**West Indian Ocean islands**	Antaisaka	8	[25]
**West Indian Ocean islands**	Merina	9	[25]
**West Indian Ocean islands**	Comoros	291	[26]
**West Indian Ocean islands**	Mikea	59	[27]
**West Indian Ocean islands**	Vezo Northern Tulear	16	[27]
**West Indian Ocean islands**	Vezo Southern Tulear	32	[27]
**West Indian Ocean islands**	Tsimahafotsy	5	[27]
**West Indian Ocean islands**	Andriana	21	[27]
**Africa CWS**	Equatorial Guinea	101	[6]
**Africa CWS**	Gabon	828	[46]
**Africa CWS**	Benin	78	[7]
**Africa CWS**	Ivory coast	90	[7]
**Africa CWS**	African Central Republic	165	[47]
**Africa CWS**	Guinea-Bissau	161	[48]
**Africa CWS**	Burkina Faso	323	[5]
**Africa CWS**	Mozambique	154	[49]
**Africa CWS**	Angola Nyaneka	75	[2]
**Africa CWS**	Angola Ovimbudu	96	[2]
**Africa CWS**	Angola various	65	[2]
**Africa CWS**	Zambia Eastern	88	[5]
**Africa CWS**	Zambia Western Bantu east	263	[5]
**Africa CWS**	Zambia Western Bantu west	192	[5]
**Africa CWS**	Namibia	54	[50]
**Eastern Africa**	Ethiopia Omo valley	67	[5]
**Eastern Africa**	Kenya Massai	55	[5]
**Eastern Africa**	Tanzania Sandawa	66	[51]
**Eastern Africa**	Tanzania various	132	[51]
**Eastern Africa**	Uganda	118	[13]
**Northern Africa**	Lybia	63	[16]
**Northern Africa**	Morocco	515	[17]
**Northern Africa**	Morocco Arabs various	104	[52]
**Northern Africa**	Mozabites	68	[53]
**Northern Africa**	Tunisia Sfax various	105	[54]
**Middle East**	Iran Iranians	128	[55]

**Middle East**	Druze d'Israel	234	[29]
**Middle East**	Liban	650	[29,33]
**Middle East**	Palestine	364	[17]
**Middle East**	Koweït	148	[30]
**Middle East**	Oman	99	[56]
**Middle East**	Saudi Arabia	106	[56]
**Middle East**	Yemen	104	[56]
**Middle East**	Dubai	217	[56]
**Middle East**	Iran various	104	[56]
**Middle East**	Cyprus	163	[33]
**Middle East**	Syria Syrians	161	[33]
**Middle East**	Turks various	140	[57]
**Middle East**	Turks Anatolia	520	[40]
**Southern Asia**	Bangladesh	284	[58]
**Southern Asia**	Saraswat Brahmin	122	[59]
**Southern Asia**	Indian Tamils Nadu	152	[60]
**Southern Asia**	Jat Sikhs	80	[61]
**Southern Asia**	India Northern	78	[22]
**Southern Asia**	India Eastern	127	[22]
**Southern Asia**	India Southern	295	[22]
**Southern Asia**	India Central	69	[22]
**Southern Asia**	India Western	59	[22]
**Southern Asia**	Mahadeokoli	50	[20]
**Southern Asia**	Thakar	48	[20]
**Southern Asia**	Pakistan	177	[22]
**Southern Asia**	Sri-Lanka	207	[62]
**Western Europe**	France	57	(Balaresque, unpublished)
**Western Europe**	Northern Portugal	60	[23]
**Western Europe**	Southern Portugal	78	[23]
**Western Europe**	Eastern Andalousia	95	[23]
**Western Europe**	Galicia	89	[23]
**Western Europe**	Northern Castilla	130	[23]
**Western Europe**	Portuguses Jew	86	[24]
**Southeastern Asia**	Philippines	76	[63]
**Southeastern Asia**	Malays Singapour	180	[64]
**Southeastern Asia**	Malays Malaysia	334	[65]
**Southeastern Asia**	Iban	105	[66]
**Southeastern Asia**	Bidayuh	113	[66]
**Southeastern Asia**	Melanau	104	[66]
**Southeastern Asia**	Timor	113	[67]
**Southeastern Asia**	Flores	385	(Karafet unpublished)
**Southeastern Asia**	Lembata	89	(Karafet unpublished)
**Southeastern Asia**	Sumba	349	(Karafet unpublished)
**Southeastern Asia**	Vietnam	113	(Karafet unpublished)
**Southeastern Asia**	Bali	632	(Karafet unpublished)

**Southeastern Asia**	Borneo	85	(Karafet unpublished)
**Southeastern Asia**	Java	61	(Karafet unpublished)
**Southeastern Asia**	Mentawai	73	(Karafet unpublished)
**Southeastern Asia**	Nias	60	(Karafet unpublished)
**Southeastern Asia**	Thailand	41	[63]
**Oceania**	Oceania various	66	(Karafet unpublished)
**Oceania**	Papouasie New Guinea	47	(Karafet unpublished)
**Oceania**	Tahiti	24	(Karafet unpublished)
**Oceania**	Vanuatu	44	(Karafet unpublished)

Table S4. J1 haplotype references used for the median-joining network

Country	Group	n	References	
**Afghanistan**	Middle East	1	[25]	
**Algeria**	Northern Africa	23	[15]	
**Saudi Arabia**	Middle East	48	[14]	
**Assyrian**	Middle East	14	[140]	
**Bali**	Southern Asia	6	(Karafet unpublished)	
**Cyprus**	Middle East	18	[17]	
**Comoros**	Comoros	18	[26]	
**Daghestan**	Europe	87	[141]	
**Egypt**	Northern Africa	29	[140]	
**Egypt**	Northern Africa	2	[140]	
**Ethiopia**	Eastern Africa	21	[141]	
**India**	Southern Asia	2	[22]	
**Iran**	Middle East	4	[140]	
**Irak**	Middle East	11	[140]	
**Irak**	Middle East	15	[141]	
**Israel**	Middle East	32	[29]	
**Israel**	Middle East	19	[140]	
**Italia**	Europe	26	[141]	
**Jordan**	Middle East	35	[140]	
**Koweït**	Middle East	1	[30]	
**Kurds**	Middle East	4	[141]	
**Liban**	Middle East	7	[29]	
**Lybia**	Northern Africa	1	[16]	Estimation
**Morocco**	Northern Africa	9	[17]	
**Morocco**	Northern Africa	9	[141]	
**Montenegro**	Europe	2	[142]	Estimation
**Oman**	Middle East	37	[140]	
**Oman**	Middle East	19	[141]	
**Pakistan**	Southern Asia	4	[22]	
**Palestine**	Middle East	100	[17]	
**Palestine**	Middle East	16	[140]	
**Parsi**	Southern Asia	3	[141]	

**Persia**	Middle East	5	[141]	
**Portugal**	Europe	22	[141]	
**Qatar**	Middle East	41	[140]	
**Qatar**	Middle East	1	[140]	
**Qatar**	Middle East	20	[141]	
**Saudi Arabia**	Middle East	4	[140]	
**Serbia**	Europe	1	[142]	Estimation
**Sudan**	Eastern Africa	26	[141]	
**Syria**	Middle East	62	[140]	
**Syria**	Middle East	8	[140]	
**Syria**	Middle East	38	[17]	
**Syria**	Middle East	9	[29]	
**Tunisia**	Northern Africa	17	[141]	
**Turkey**	Middle East	18	[40]	
**UAE**	Middle East	57	[140]	
**Yemen**	Middle East	42	[140]	
**Yemen**	Middle East	3	[140]	

Table S5. T1 haplotype references used for the median-joining network

**Country**	**Group**	**n**	**References**	
**Angola**	Subsaharan Africa	1	[143]	Estimation
**Argentina-(Native-American)**	America	1	[144]	
**Belgium**	Europe	1	(Decorte *et al.* 2004 YHRD)	
**Brazil– Alagoas (Admixed-Brazilian)**	America	10	(Azevedo 2009 YHRD)	
**British non Jefferson**	Europe	2	[145]	
**China**	Eastern Asia	1	[146]	
**China**	Eastern Asia	1	[22]	
**Egypt**	Northern Africa	4	[18]	
**Equatorial Guinea**	Subsaharan Africa	1	[6]	Estimation
**Spain**	Europe	1	[23]	Estimation
**French**	Europe	3	[141]	
**Iberic peninsula**	Europe	13	[141]	
**Israel**	Middle East	11	[29]	
**Italia-Modena**	Europe	3	(Ferri *et al.* 2008 YHRD)	
**Koweït**	Middle East	1	[30]	
**Liban**	Middle East	23	[33]	
**Lybia**	Northern Africa	2	[16]	Estimation
**Macedonia**	Europe	4	(Spiroski *et al.* 2005 ; Jakovski *et al.* 2011 YHRD)	
**Mediterranean**	Europe	1	[23]	Estimation
**Netherdlands**	Europe	1	(Decorte *et al.* 2004 YHRD)	
**Palestine**	Middle East	6	[17]	
**Portugal**	Europe	2	[22]	
**Portugal**	Europe	10	[24]	
**Syria**	Middle East	3	[17]	

**Tunisia**	Northern Africa	4	[17]	
**Turkey**	Middle East	13	[40]	
**Uganda**	Subsaharan Africa	1	[13]	
**USA-Nevada-(Basque)**	America	1	(Valverde Potes YHRD)	
**Virginia Jefferson**	Europe	6	[141]	
**Zambia**	Subsaharan Africa	1	[5]	Estimation

Table S6. HVI and HVII profiles in the Antemoro

Groups	ID	HVI	HVII	HG
**Ampanabaka**	**MA302**	16124 16223 16319	73 150 152 263	L3d
	**MA303**	16182C 16183C 16189 16223 16278 16290 16294 16390	73 146 152 195	L2a1
	**MA304**	16223 16295 16362 16519	73 146 199 263	M7c3c
	**MA305**	16093 16223 16278 16362 16519	73 263	L3b
	**MA307**	16223 16278 16311 16362 16519	73 263	L3b
	**MA308**	16223 16265T 16519	73 150 195 263	L3e3
	**MA311**	16172 16183C 16189 16213 16223 16320 16519	73 150 152 195	L3e2b3
	**MA312**	16223 16295 16362 16519	73 146 199 263	M7c3c
	**MA313**	16086 16148 16223 16259 16278 16311 16319 16399 16526	73 150 200 263	M32c
	**MA 315**	16189 16192 16223 16256 16278 16294 16309 16344 16390 16519	73 146 152 195 263	L2a1
	**MA316**	16148 16172 16187 16188G 16189 16223 16230 16311 16320 16519	64 93 152 185 189 204 207 247 263	L0a2
	**MA317**	16182C 16183C 16189 16217 16247 16261 16519	73 146 195	B4a1a1a
	**MA318**	16223 16265T 16519	73 150 195 263	L3e3
	**MA320**	16129 16182C 16183C 16189 16217 16247 16261 16290 16519	71 73 146	B4a1a1a
	**MA322**	16182C 16183C 16189 16217 16247 16261 16519	73 146	B4a1a1a
	**MA323**	16182C 16183C 16189 16217 16247 16261 16519	73 146	B4a1a1a
	**MA324**	16148 16172 16187 16188G 16189 16223 16230 16311 16320 16519	64 93 152 189 204 207 236 247 263	L0a2
	**MA325**	16182C 16183C 16189 16217 16247 16261 16519	73 146	B4a1a1a
	**MA326**	16182C 16183C 16189 16217 16247 16261 16519	73 146	B4a1a1a
	**MA327**	16182C 16183C 16189 16217 16247 16261 16519	73 146	B4a1a1a
	**MA328**	16220C 16265 16298 16362	73 150 152 200 249d 263	F3b
	**MA329**	16182C 16183C 16189 16217 16247 16261 16519	73 146 252	B4a1a1a
	**MA330**	16093 16223 16278 16362 16519	73 263	L3b
	**MA331**	16093 16223 16278 16362 16519	73 263	L3b
	**MA332**	16223 16263 16311 16519	73 152 195 204	M23
	**MA333**	16220C 16265 16298 16362	73 150 152 200 249d 263	F3b

	**MA335**	16223 16263 16311 16519	73 152 195 204 263	M23
	**MA337**	16182C 16183C 16189 16223 16278 16290 16294 16309 16390	73 146 152 195	L2a1
	**MA338**	16182C 16183C 16189 16217 16247 16261 16519	73 146	B4a1a1a
	**MA339**	16093 16124 16217 16223 16278 16362 16519	73 263	L3b
	**MA340**	16189 16223 16278 16294 16309 16390 16519	73 146 152 195 198	L2a1
	**MA341**	16221 16223 16291 16362 16390 16519	73 263	E1a
	**MA342**	16223 16295 16362 16519	73 146 199 263	M7c3c
	**MA343**	16182C 16183C 16189 16217 16247 16261 16519	73 146	B4a1a1a
	**MA344**	16182C 16183C 16189 16217 16247 16261 16519	73 146	B4a1a1a
	**MA345**	16223 16295 16362 16519	73 146 199 263	M7c3c
	**MA346**	16185 16223 16327 16519	73 150 189 200 263	L3e1a
	**MA349**	16182C 16183C 16189 16223 16278 16290 16294 16309 16390	73 146 152 195	L2a1
	**MA350**	16223 16265T 16519	73 150 195 263	L3e3
	**MA351**	16148 16172 16187 16188G 16189 16223 16230 16311 16320 16519	64 93 152 189 204 207 236 247 263	L0a2
	**MA352**	16093 16223 16278 16362 16519	73 263	L3b
	**MA353**	16221 16223 16291 16362 16390 16519	73 263	E1a
	**MA354**	16220C 16265 16298 16362	73 150 152 249d 263	F3b
	**MA355**	16223 16265T 16519	73 150 195 263	L3e3
	**MA356**	16172 16183C 16189 16213 16223 16320 16519	73 150 152 195	L3e2b3
	**MA408**	16185 16223 16327 16519	73 150 189 200 263	L3e1a
	**MA452**	16172 16223 16295 16362 16359	73 146 199 263	M7c3c
	**MA358**	16182C 16183C 16189 16217 16247 16261 16519	73 146	B4a1a1a
**Anteony**	**MA359**	16093 16172 16223 16278 16311 16362 16519	73 263	L3b
	**MA360**	16129 16148 16168 16172 16187 16188G 16189 16223 16230 16278 16293 16311 16320	93 95C 185 189 236 247 263	L0a1’4
	**MA361**	16223 16263 16311 16519	73 152 195 204 263	M23
	**MA362**	16093 16223 16278 16362 16519	73 263	L3b
	**MA363**	16220C 16265 16298 16362	73 150 152 249d 263	F3b
	**MA364**	16169 16213 16223 16240C 16254 16316 16335	73 152 263	L3a
	**MA365**	16182C 16183C 16189 16217 16247 16261 16291 16519	73 146	B4a1a1a

**MA367**	16223 16278 16362 16519	73 263	L3b
**MA369**	16220C 16265 16298 16362	73 150 152 200 249d 263	F3b
**MA370**	16223 16263 16311 16519	73 152 195 204 263	M23
**MA371**	16182C 16183C 16189 16217 16247 16261 16519	73 146	B4a1a1a
**MA373**	16093 16223 16274 16278 16362 16519	73 263	L3b
**MA374**	16265T 16292 16519	73 150 195 263	L3e3
**MA375**	16172 16183C 16189 16223 16320 16519	73 150 195	L3e2b
**MA376**	16182C 16183C 16189 16223 16278 16290 16294 16309 16390	73 146 152 195	L2a1
**MA377**	16172 16223 16263 16311 16519	73 152 195 204 263	M23
**MA378**	16124 16223 16319	73 150 152 263	L3d
**MA380**	16223 16278 16362 16519	73 263	L3b
**MA381**	16223 16295 16362 16519	73 146 199 263	M7c3c
**MA382**	16220C 16265 16298 16362	73 150 152 249d 263	F3b
**MA383**	16182C 16183C 16189 16223 16278 16290 16294 16309 16390	73 146 152 195	L2a1
**MA384**	16182C 16183C 16189 16217 16261 16519	73 146	B4a1a1a
**MA385**	16129 16148 16168 16172 16187 16188G 16189 16223 16230 16278 16293 16311 16320	93 95C 185 189 194G 236 247 263	L0a1'4
**MA386**	16086 16148 16223 16259 16278 16319 16399 16526	73 150 195 200 263	M32c
**MA388**	16223 16266 16278 16294 16309 16390 16519	73 146 152 195 263	L2a1
**MA389**	16223 16278 16362 16519	73 263	L3b
**MA390**	16189 16265T 16292 16519	73 150 195	L3e3
**MA391**	16220C 16265 16298 16362	73 150 152 249d 263	F3b
**MA392**	16182C 16183C 16189 16223 16278 16290 16294 16309 16390	73 146 152 195	L2a1
**MA393**	16182C 16183C 16189 16217 16247 16261 16291 16519	73 146	B4a1a1a
**MA394**	16172 16183C 16189 16213 16223 16245 16320 16519	73 150 152 195	L3e2b3
**MA395**	16182C 16183C 16189 16223 16278 16290 16294 16390	73 146 195	L2a1
**MA396**	16093 16223 16278 16362 16519	73 263	L3b
**MA397**	16129 16148 16168 16172 16187 16188G 16189 16223 16230 16278 16293 16311 16320	93 95C 185 189 236 247 263	L0a1'4
**MA398**	16093 16223 16278 16362 16519	73 263	L3b
**MA399**	16182C 16183C 16189 16223 16278 16290 16294 16309 16390 16519	73 146 152 195	L2a1

	**MA400**	16172 16223 16263 16311 16519	73 152 195 204 263	M23
	**MA403**	16172 16183C 16189 16213 16223 16320 16519	73 150 152 195	L3e2b3
	**MA405**	16129 16144 16148 16192 16223 16241 16265C 16274 16311 16343 16362	73 89 146 238 263	Q1
	**MA406**	16093 16223 16355	73 150 152 235 263	L3k
	**MA409**	16148 16172 16187 16188G 16189 16223 16230 16311 16320 16519	64 93 152 189 204 207 236 247 263	L0a2
	**MA410**	16185 16223 16327 16519	73 150 189 200 263	L3e1a
	**MA411**	16220C 16265 16298 16362	73 150 152 200 249d 263	F3b
	**MA412**	16223 16263 16311 16519	73 152 195 204 263	M23
	**MA414**	16182C 16183C 16189 16217 16247 16261 16291 16519	73 146	B4a1a1a
	**MA415**	16189 16192 16223 16256 16278 16294 16309 16344 16390 16519	73 146 152 195	L2a1
	**MA436**	16220C 16265 16298 16362	73 150 152 249d 263	F3b
**Antalaotra**	**MA407**	16223 16263 16311 16519	73 152 195 204 263	M23
	**MA416**	16223 16265T 16519	73 150 152 195 263	L3e3
	**MA417**	16093 16223 16278 16362 16399 16519	73 263	L3b
	**MA418**	16182C 16183C 16189 16217 16247 16261 16519	73 146 263	B4a1a1a
	**MA420**	16182C 16183C 16189 16217 16247 16261 16519	73 146	B4a1a1a
	**MA421**	16223 16265T 16519	73 150 195 263	L3e3
	**MA422**	16093 16223 16278 16362 16519	73 263	L3b
	**MA423**	16182C 16183C 16189 16217 16247 16261 16519	73 146	B4a1a1a
	**MA424**	16172 16182C 16189 16213 16223 16245 16320 16519	73 150 152 195	L3e2b3
	**MA425**	16182C 16183C 16189 16223 16278 16290 16294 16309 16390	73 146 152 195	L2a1
	**MA426**	16093 16223 16295 16362 16519	73 146 199 263	M7c3c
	**MA427**	16182C 16183C 16189 16217 16247 16261 16519	73 146	B4a1a1a
	**MA428**	16086 16148 16189 16223 16259 16278 16319 16399 16526	73 150 200	M32c
	**MA429**	16182C 16183C 16189 16223 16278 16290 16294 16309 16390	73 146 152 195	L2a1
	**MA430**	16148 16172 16187 16188G 16189 16223 16230 16311 16320 16519	64 93 150 152 189 204 207 236 247 263	L0a2
	**MA432**	16093 16223 16278 16362	73 263	L3b
	**MA435**	16182C 16183C 16189 16217 16247 16261 16519	73 146	B4a1a1a
	**MA438**	16172 16183C 16189 16213 16223 16245 16320 16519	73 150 152 195	L3e2b3

**MA439**	16182C 16183C 16189 16217 16247 16261 16519	73 146 199	B4a1a1a
**MA440**	16182C 16183C 16189 16217 16261 16519	73 146	B4a1a1a
**MA441**	16223 16263 16311 16519	73 151 152 195 204 263	M23
**MA442**	16148 16172 16187 16188G 16189 16223 16230 16311 16320 16519	64 93 150 152 189 204 207 236 247 263	L0a2
**MA443**	16182C 16183C 16189 16217 16247 16261 16519	73 146	B4a1a1a
**MA445**	16213 16220C 16265 16298 16362	73 150 152 249d 263	F3b
**MA447**	16093 16223 16278 16362 16519	73 263	L3b
**MA448**	16086 16148 16223 16259 16278 16319 16399 16526	73 150 200 263	M32c
**MA449**	16223 16295 16362 16359	73 146 199 263	M7c3c
**MA450**	16223 16263 16311 16359	73 152 195 204 263	M23
**MA451**	16221 16223 16291 16362 16390 16359	73 263	E1a
**MA453**	16223 16263 16311 16519	73 152 195 204 263	M23
**MA455**	16220C 16265 16298 16362	73 150 152 249d 263	F3b
**MA456**	16086 16148 16223 16259 16278 16319 16399 16526	73 150 200 263	M32c
**MA457**	16213 16220C 16265 16298 16362	73 150 152 249d 263	F3b
**MA459**	16220C 16265 16298 16362	73 150 152 249d 263	F3b
**MA461**	16265T 16292 16519	73 150 195 263	L3e3
**MA462**	16223 16263 16311 16519	73 152 195 204 263	M23
**MA464**	16093 16223 16278 16291 16362 16519	73 263	L3b
**MA465**	16220C 16265 16298 16362	73 150 152 249d 263	F3b
**MA466**	16182C 16183C 16189 16217 16247 16261 16519	73 146	B4a1a1a
**MA469**	16093 16223 16278 16362 16519	73 263	L3b

Table S7. Database used for HVI analysis

Georaphic Group	Population	n	References
**West Indian Ocean islands**	Highlands	52	[28] [25,27]
**West Indian Ocean islands**	Antanosy	54	[25]
**West Indian Ocean islands**	Antandroy	59	[25]
**West Indian Ocean islands**	Mikea	127	[27]
**West Indian Ocean islands**	Vezo Northern Tuléar	52	[27]
**West Indian Ocean islands**	Vezo Southern Tuléar	49	[27]
**Africa CWS**	Angola Nyaneka	147	[2]
**Africa CWS**	Angola Ovimbudu	98	[2]
**Africa CWS**	Fon Benin	171	[7,68]
**Africa CWS**	Cabinda Angola	110	[1]
**Africa CWS**	Cabo Verde	292	[69]
**Africa CWS**	Cameroon Ngumba	88	[70]
**Africa CWS**	Cameroon Pygmies Baka	87	[70]
**Africa CWS**	Cameroon Pygmies Bakoka	88	[70]
**Africa CWS**	Gabon Fang	66	[70]
**Africa CWS**	Gabon Mitsogo	64	[70]
**Africa CWS**	Gabon Nzebi	63	[70]
**Africa CWS**	Guinea Bissau	372	[71]
**Africa CWS**	Ivory coast Ahizi	129	[7]
**Africa CWS**	Ivory coast Yacouba	61	[7]
**Africa CWS**	Mali Malinke	60	[72]
**Africa CWS**	Mozambique	416	[73] [74]
**Africa CWS**	Rwanda Hutu	106	[75] [68]
**Africa CWS**	Sao Tome	103	[76]
**Africa CWS**	Senegal Mandenka	119	[77]
**Africa CWS**	Senegal Wolof	91	[78]
**Africa CWS**	South Africa Kung	59	[79]
**Africa CWS**	Zimbabwe	58	[75]
**Northern Africa**	Guanche Canaries island	71	[80]
**Northern Africa**	Egypt Arabs	102	(Coudray unpublished)
**Northern Africa**	Egypt Coptes	100	(Coudray unpublished)
**Northern Africa**	Maures Mauritania	64	[81]
**Northern Africa**	Morocco Berbers	181	[78] [82]
**Northern Africa**	Tunisia	47	[83]
**Northern Africa**	Algeria	47	[84]
**Northern Africa**	Sahawari	56	[83]
**Northern Africa**	Samaritans	124	[85]
**Northern Africa**	Tunisia Various	102	[86] [83]
**Northern Africa**	Morocco Berbers Bourhiah	70	[87]
**Northern Africa**	Morocco Berbers Figuig	94	[87]

**Northern Africa**	Egypt Berbers Siwa	78	[87]
**Northern Africa**	Libya Tuaregs	129	[85]
**Eastern Africa**	Kenya Swahili	200	[88]
**Eastern Africa**	Tanzania Hadza	79	[51]
**Eastern Africa**	Tanzania Sandawe	82	[51]
**Eastern Africa**	Ethiopia Amharic Various	270	[89]
**Eastern Africa**	Sudan Nubia	161	[89] [90]
**Eastern Africa**	Ethiopia various	116	[91]
**Middle East**	Soqotra	65	[31]
**Middle East**	Iran	146	[92,93]
**Middle East**	Syria	49	[94]
**Middle East**	Yemen	115	[89]
**Middle East**	Israel Druzes	45	[95]
**Middle East**	Kurds	78	[92,96]
**Middle East**	Cyprus Greek	91	[97]
**Middle East**	Irak	52	[36]
**Middle East**	Iranian	731	[92,98] (Balloux unpublished)
**Middle East**	Dubai	249	[99]
**Middle East**	Israel Druzes	311	[29]
**Middle East**	Turkey various	234	[92,100,101]
**Middle East**	Jordan	99	[102]
**Middle East**	Iran Kurds	25	[103]
**Middle East**	Iran Persians	82	[103]
**Middle East**	Saudi Arabia	553	[104]
**Southern Asia**	Bangladesh	30	[105]
**Southern Asia**	Gujarat	91	[106]
**Southern Asia**	India Kamataka	201	[107] [106] [98]
**Southern Asia**	India Kerala	230	[108] [109] [106] [98] [107]
**Southern Asia**	Madyah Pradesh	82	[98] [108] [110]
**Southern Asia**	India Maharashtra	221	[98,107,108,111] [110]
**Southern Asia**	India Orissa	153	[112] [98] [108]
**Southern Asia**	Punjab	362	[108] [107] [113]
**Southern Asia**	Tamils Nadu	427	[108] [107] [109]
**Southern Asia**	India Tripura	134	[108] [107] [112]
**Southern Asia**	Uttard Pradesh	232	[108] [112] [98] [109,114]
**Southern Asia**	Western Bengali	285	[108] [98] [109]
**Southern Asia**	Sri Lanka	131	[115]
**Southern Asia**	Hindus India	72	[116]
**Western Europe**	Sicilia	226	[117] (Rickards *et al.* unpublished)
**Western Europe**	Galicia	92	[118]
**Western Europe**	Catalan	46	[83]
**Western Europe**	Andalusia	115	[119] [84]
**Western Europe**	Portuguese	54	[120])
**Western Europe**	Corsica	53	[121]
**Western Europe**	France Southern	110	[122]

**Western Europe**	England	100	[123]
**Western Europe**	Greece	114	[94,117] (Rickards *et al.* unpublished)
**Southeastern Asia**	Thailand various	71	[124]
**Southeastern Asia**	Vietnam	65	[125]
**Southeastern Asia**	Adonara	73	[44]
**Southeastern Asia**	Flores	73	[44]
**Southeastern Asia**	Banjarmasin	110	[28,126]
**Southeastern Asia**	Bali	64	[126]
**Southeastern Asia**	Sumatra Medan	42	[127]
**Southeastern Asia**	Sumatra Pekanbaru	54	[127]
**Southeastern Asia**	Nicobar various	46	[128]
**Southeastern Asia**	Onges Nicobar	63	[128]
**Southeastern Asia**	Philippines	144	[126,129,130]
**Southeastern Asia**	Iban	83	[131]
**Southeastern Asia**	Malays Kuala Lumpur	124	[132]
**Southeastern Asia**	Malays Singapore	205	[133]
**Oceania**	New Guinea highlands	71	[134]
**Oceania**	Solomon islands	64	[135]
**Oceania**	Papouasie New Guinea Province Manus	144	[136]
**Oceania**	New Guinea Gidra	59	[137]
**Oceania**	Archipelago Bismark	47	[138]
**Oceania**	Vanuatu Nguna	42	[139]

Table S8. Table of population pairwise F_{ST} values based on Y haplogroup frequencies.

	**Ampanabaka**		**Antalaotra**		**Anteony**	
	**F_{ST}**	**Significativity threashold of 5%**	**F_{ST}**	**Significativity threashold of 5%**	**F_{ST}**	**Significativity threashold of 5%**
**Ampanabaka**	*	*				
**Antalaotra**	0.31664	+	*	*		
**Anteony**	0.43138	+	0.16051	+	*	*
**Antandroy**	0.00750	-	0.26312	+	0.38024	+
**Antanosy**	0.05669	+	0.11941	+	0.26578	+
**Comoros**	0.08978	+	0.11477	+	0.22337	+
**Mikea Mikea-vezo**	0.06864	+	0.16897	+	0.31891	+
**Vezo Northern Tulear**	0.01617	-	0.30595	+	0.42315	+
**Vezo Southern Tuelear**	-0.00810	-	0.24402	+	0.36632	+
**HighlandsMadagascar**	0.13919	+	0.04064	+	0.21931	+
**Angola Cabinda**	0.02905	-	0.37818	+	0.46437	+
**Angola Nyaneka-Nkhumbi**	0.04965	+	0.47172	+	0.55806	+
**Angola Ovimbudu**	0.04502	+	0.47291	+	0.54891	+
**DRC Mbuti Pygmies**	0.19099	+	0.19990	+	0.31638	+
**South Africa!Kung**	0.21482	+	0.23373	+	0.32574	+
**South Africa !Khwe**	0.11796	+	0.24889	+	0.33064	+
**Rwanda Hutu**	0.04186	+	0.44910	+	0.53877	+
**Rwanda Tutsi**	0.03599	+	0.41178	+	0.49665	+
**Mozambique various**	0.51330	+	0.34020	+	0.43975	+
**Zambia Eastern**	0.07148	+	0.49647	+	0.59311	+
**Western Zambia – Western Bantus**	0.06430	+	0.54129	+	0.61501	+
**Western Zambia – Eastern Bantus**	0.05373	+	0.54481	+	0.60938	+
**Equatorial Guinea various**	0.05077	+	0.27790	+	0.35313	+
**Benin**	0.08655	+	0.52084	+	0.61642	+
**Burkina Faso Fulbe**	0.08617	+	0.52545	+	0.61028	+
**Burkina Faso Mossi**	0.06706	+	0.51434	+	0.60214	+
**Burkina Faso Rimaibe**	0.09962	+	0.28083	+	0.39300	+
**Cameroon Bakaka**	0.23876	+	0.66630	+	0.75939	+
**Cameroon Bamileke**	0.13920	+	0.60423	+	0.69322	+
**Cameroon Daba**	0.28776	+	0.19870	+	0.31379	+
**Cameroon Ewondo**	0.05113	+	0.48739	+	0.57060	+
**Cameroon Fali**	0.09440	+	0.27346	+	0.35516	+
**Cameroon Fulbe**	0.47428	+	0.28597	+	0.38403	+
**Cameroon variousAdamawa**	0.34336	+	0.27556	+	0.36619	+
**Cameroon variousChadic**	0.46034	+	0.32345	+	0.41704	+
**Cameroon Nilo-Saharan**	0.24091	+	0.21405	+	0.31570	+
**Cameroon Tali**	0.06456	+	0.36200	+	0.44236	+
**Cameroon Uldeme**	0.79597	+	0.62187	+	0.72117	+
**African central republic Lissongo**	0.05395	+	0.46659	+	0.54180	+
**Cabo Verde - Northern**	0.29012	+	0.12558	+	0.20651	+

**Cabo Verde - Southern**	0.23313	+	0.10514	+	0.17929	+
**Benin Fon**	0.13144	+	0.58629	+	0.67810	+
**Cameroon Bantus**	0.04331	+	0.40278	+	0.48384	+
**Senegal various**	0.03774	+	0.43688	+	0.51343	+
**Tanzania Datoga**	0.60676	+	0.42811	+	0.51671	+
**Tanzania Hadzabe**	0.45534	+	0.27686	+	0.37314	+
**Kenya Bantus**	0.15430	+	0.16585	+	0.27665	+
**Tanzania Wairak**	0.17815	+	0.21737	+	0.28183	+
**Kenya**	0.19471	+	0.22983	+	0.31374	+
**Karamoja. Uganda**	0.44809	+	0.30825	+	0.41177	+
**Maasai Kenya nilotic**	0.22230	+	0.16771	+	0.26855	+
**Ethiopia Juifs**	0.40942	+	0.23052	+	0.32333	+
**Ethiopia Amhara**	0.41147	+	0.14938	+	0.22106	+
**Ethiopia Oromo**	0.49116	+	0.29143	+	0.37010	+
**Ethiopia Omo valley**	0.38852	+	0.19662	+	0.28973	+
**Somalia**	0.63607	+	0.48336	+	0.53339	+
**Namibia**	0.18402	+	0.23885	+	0.32237	+
**Egypt**	0.34173	+	0.13121	+	0.18784	+
**Algeria various**	0.39096	+	0.20186	+	0.28183	+
**Lybia Arabs**	0.43031	+	0.19312	+	0.27595	+
**Morocco Arabs**	0.60594	+	0.42688	+	0.51556	+
**Morocco Berbers**	0.60035	+	0.43620	+	0.52345	+
**Morocco**	0.45937	+	0.32352	+	0.39495	+
**Tunisia**	0.43236	+	0.21776	+	0.28789	+
**Pakistan**	0.31252	+	0.11930	+	0.22293	+
**Naikpod**	0.40489	+	0.16888	+	0.29755	+
**Andh**	0.43974	+	0.20162	+	0.33258	+
**Pardhan**	0.35015	+	0.13551	+	0.25636	+
**Eastern India**	0.49195	+	0.15278	+	0.36686	+
**Mahadeokoli**	0.42581	+	0.18809	+	0.30773	+
**Thakar**	0.39601	+	0.15443	+	0.28487	+
**Muslim Shia**	0.34031	+	0.12980	+	0.23568	+
**Muslim Sunni**	0.38237	+	0.17733	+	0.29006	+
**India - Northern**	0.39619	+	0.17920	+	0.29491	+
**India – Northeastern**	0.53624	+	0.30591	+	0.43662	+
**India Eastern**	0.36464	+	0.10510	+	0.26464	+
**India - Southern**	0.36052	+	0.16020	+	0.27869	+
**India Central**	0.51501	+	0.25087	+	0.40558	+
**India Western**	0.38965	+	0.16273	+	0.28280	+
**Northern Portugal**	0.47259	+	0.25603	+	0.36366	+
**Southern Portugal**	0.40566	+	0.19940	+	0.30601	+
**Potuguais Jew**	0.37331	+	0.14482	+	0.20813	+
**France**	0.61501	+	0.40361	+	0.51194	+
**Basque**	0.70540	+	0.53092	+	0.62689	+
**Eastern Andalusia**	0.56320	+	0.36623	+	0.46870	+

**Galicia**	0.47320	+	0.27454	+	0.37517	+
**North-western Castille**	0.47906	+	0.28512	+	0.38460	+
**Iranian Arabs**	0.41161	+	0.17291	+	0.30011	+
**Israel Druzes Galilea**	0.31495	+	0.11175	+	0.20651	+
**Palestinian**	0.35795	+	0.13180	+	0.20609	+
**Kuwait Bedouins**	0.62755	+	0.36344	+	0.42317	+
**Socotra various**	0.67615	+	0.37920	+	0.45852	+
**Saudi Arabia**	0.32832	+	0.10334	+	0.16407	+
**Qatar**	0.44756	+	0.16156	+	0.24598	+
**UAE**	0.31207	+	0.09138	+	0.15462	+
**Oman**	0.32688	+	0.09330	+	0.14189	+
**Yemen**	0.55627	+	0.26304	+	0.34006	+
**Lebanon**	0.30399	+	0.10206	+	0.17749	+
**Jordan**	0.36087	+	0.13027	+	0.21427	+
**Irak**	0.34062	+	0.10419	+	0.17676	+
**Iran**	0.30900	+	0.09850	+	0.19561	+
**Syrians**	0.32949	+	0.10617	+	0.17475	+
**Iranians**	0.30729	+	0.11204	+	0.20406	+
**Cyprus**	0.35103	+	0.13438	+	0.22146	+
**Turkey various**	0.36506	+	0.13491	+	0.24827	+
**Turkey Anatolia**	0.29780	+	0.10435	+	0.19111	+
**Vanuatu-Maewo**	0.40438	+	0.17320	+	0.29166	+
**South-western New Guinea Una**	0.79807	+	0.58138	+	0.70009	+
**PNG Trobriand**	0.37876	+	0.14090	+	0.29352	+
**PNG Kapuna**	0.54444	+	0.32102	+	0.43240	+
**Cook**	0.62744	+	0.42289	+	0.52956	+
**Futuna**	0.43829	+	0.21717	+	0.32559	+
**Fidji**	0.39384	+	0.19448	+	0.30165	+
**Tuvalu**	0.43450	+	0.23512	+	0.33990	+
**Western Samoa**	0.49305	+	0.27656	+	0.38964	+
**Nias**	0.93541	+	0.82276	+	0.91999	+
**Philippines**	0.45563	+	0.21794	+	0.34959	+
**Vietnam**	0.41420	+	0.12518	+	0.30188	+
**Bali**	0.42772	+	0.10718	+	0.35497	+
**Java**	0.41636	+	0.06734	+	0.30572	+
**Borneo**	0.38009	+	0.12610	+	0.28195	+
**Nias various**	0.78733	+	0.52270	+	0.73189	+
**Mentawai**	0.64605	+	0.39032	+	0.60254	+
**Flores**	0.37058	+	0.19134	+	0.30136	+
**Sulawesi**	0.33712	+	0.07163	+	0.24091	+
**Sumba**	0.44165	+	0.27408	+	0.37796	+
**Lembata**	0.43049	+	0.23261	+	0.33676	+
**Adonara various (Austronesian)**	0.73845	+	0.55727	+	0.65824	+
**Flores various (Austronesian)**	0.50397	+	0.29944	+	0.40306	+
**Solor various (Austronesian)**	0.45657	+	0.22868	+	0.33974	+

**Sumatra**	0.35285	+	0.09115	+	0.25245	+
**Java various**	0.40449	+	0.06689	+	0.29906	+
**Negritos**	0.36292	+	0.16059	+	0.30252	+
**Non Negritos**	0.43808	+	0.21848	+	0.41114	+
**Malay**	0.42278	+	0.09586	+	0.28927	+

Table S9. Shared haplotypes between the Ampanabaka, other Malagasy populations and the Comoros, using 17 Y-STR markers

	Ampanabaka	Antaisaka	Antandroy	Antanosy	Comoros	Andriana	Merina	Tsimahafotsy	Mikea	Vezo - Northern	Vezo - Southern
**N ind.**	**46**	**8**	**46**	**47**	**293**	**21**	**9**	**5**	**59**	**16**	**31**
**N hap.**	**36**	**7**	**36**	**47**	**250**	**13**	**8**	**3**	**44**	**12**	**28**
**H1**	6	0	0	0	0	0	0	0	0	0	0
**H2**	5	0	0	0	0	0	0	0	0	0	0
**H3**	1	0	0	0	0	0	0	0	0	0	0
**H4**	1	0	0	0	0	0	0	0	0	0	0
**H5**	2	0	0	0	0	0	0	0	0	0	0
**H6**	1	0	0	0	0	0	0	0	0	0	0
**H7**	1	0	0	0	0	0	0	0	0	0	0
**H8**	1	0	0	0	0	0	0	0	0	0	0
**H9**	1	0	0	0	0	0	0	0	0	0	0
**H10**	1	0	0	0	0	0	0	0	0	0	0
**H11**	1	0	0	0	0	0	0	0	0	0	0
**H12**	1	0	0	0	0	0	0	0	0	0	0
**H13**	1	0	0	0	0	0	0	0	0	0	0
**H14**	1	0	0	0	0	0	0	0	0	0	0
**H15**	1	0	0	0	0	0	0	0	0	0	0
**H16**	1	0	0	0	0	0	0	0	0	0	0
**H17**	1	0	0	0	0	0	0	0	0	0	0
**H18**	1	0	0	0	0	0	0	0	0	0	0
**H19**	1	0	0	0	0	0	0	0	0	0	0
**H20**	1	0	0	0	0	0	0	0	0	0	0
**H21**	1	0	0	0	0	0	0	0	0	0	0
**H22**	1	0	0	0	0	0	0	0	0	0	0
**H23**	1	0	0	0	0	0	0	0	0	0	0
**H24**	1	0	0	0	0	0	0	0	0	0	0
**H25**	1	0	0	0	0	0	0	0	0	0	0
**H26**	1	0	0	0	0	0	0	0	0	0	0
**H27**	1	0	0	0	0	0	0	0	0	0	0
**H28**	1	0	0	0	0	0	0	0	0	0	0
**H29**	1	0	0	0	0	0	0	0	0	0	0
**H30**	1	0	1	0	0	0	0	0	0	0	0
**H31**	1	0	0	0	0	0	0	0	0	0	0
**H32**	1	0	0	1	0	0	0	0	0	0	0
**H33**	1	0	0	0	0	0	0	0	0	0	0
**H34**	1	0	0	0	0	0	0	0	0	0	0
**H35**	1	0	0	0	0	0	0	0	0	0	0
**H36**	1	0	0	0	0	0	0	0	0	0	0

Table S10. Shared haplotypes between the Anteony, other Malagasy populations and the Comoros, using 17 Y-STR markers

	**Anteony**	**Antaisaka**	**Antandroy**	**Antanosy**	**Comoros**	**Andriana**	**Merina**	**Tsimahafotsy**	**Mikea**	**Vezo - Northern**	**Vezo - Southern**
**N ind.**	**40**	**8**	**46**	**47**	**293**	**21**	**9**	**5**	**59**	**16**	**31**
**N hap.**	**21**	**7**	**36**	**47**	**250**	**13**	**8**	**3**	**44**	**12**	**28**
**H1**	8	0	0	0	0	0	0	0	0	0	0
**H2**	8	0	0	0	0	0	0	0	0	0	0
**H3**	5	0	0	0	0	0	0	0	0	0	0
**H4**	1	0	0	0	0	0	0	0	0	0	0
**H5**	1	0	0	0	0	0	0	0	0	0	0
**H6**	1	0	0	0	0	0	0	0	0	0	0
**H7**	2	0	0	0	0	0	0	0	0	0	0
**H8**	1	0	0	0	0	0	0	0	0	0	0
**H9**	1	0	0	0	0	0	0	0	0	0	0
**H10**	1	0	0	0	0	0	0	0	0	0	0
**H11**	1	0	0	0	0	0	0	0	0	0	0
**H12**	1	0	0	0	0	0	0	0	0	0	0
**H13**	1	0	0	0	0	0	0	0	0	0	0
**H14**	1	0	0	0	0	0	0	0	0	0	0
**H15**	1	0	0	0	0	0	0	0	0	0	0
**H16**	1	0	0	0	0	0	0	0	0	0	0
**H17**	1	0	0	0	0	0	0	0	0	0	0
**H18**	1	0	0	0	0	0	0	0	0	0	0
**H19**	1	0	0	0	0	0	0	0	0	0	0
**H20**	1	0	0	0	0	0	0	0	0	0	0
**H21**	1	0	0	0	0	0	0	0	0	0	0

Table S11. Shared haplotypes between the Antalaotra, other Malagasy populations and the Comoros, using 17 Y-STR markers.

	Antalaotra	Antaisaka	Antandroy	Antanosy	Comoros	Andriana	Merina	Tsimahafotsy	Mikea	Vezo - Northern	Vezo - Southern
**N ind.**	**43**	**8**	**46**	**47**	**293**	**21**	**9**	**5**	**59**	**16**	**31**
**N hap.**	**35**	**7**	**36**	**47**	**250**	**13**	**8**	**3**	**44**	**12**	**28**
**H1**	4	0	0	0	0	0	0	0	0	0	0
**H2**	1	0	0	0	0	0	0	0	0	0	0
**H3**	1	0	0	0	0	0	0	0	0	0	0
**H4**	1	0	0	0	0	0	0	0	0	0	0
**H5**	3	0	0	0	0	0	0	0	0	0	0
**H6**	1	0	0	0	0	0	0	0	0	0	0
**H7**	2	0	0	0	0	0	0	0	0	0	0
**H8**	2	0	0	0	0	0	0	0	0	0	0
**H9**	1	0	0	0	0	0	0	0	0	0	0
**H10**	2	0	0	0	0	0	0	0	0	0	0
**H11**	1	0	0	0	0	0	0	0	0	0	0
**H12**	1	0	0	0	0	0	0	0	0	0	0
**H13**	1	0	0	0	0	0	0	0	0	0	0
**H14**	1	0	0	0	0	0	0	0	0	0	0
**H15**	1	0	0	0	0	0	0	0	0	0	0
**H16**	1	0	0	0	0	0	0	0	0	0	0
**H17**	1	0	0	0	0	0	0	0	0	0	0
**H18**	1	0	0	0	0	0	0	0	0	0	0
**H19**	1	0	0	0	0	0	0	0	0	0	0
**H20**	1	0	0	0	0	0	0	0	0	0	0
**H21**	1	0	0	0	0	0	0	0	0	0	0
**H22**	1	0	0	0	0	0	0	0	0	0	0
**H23**	1	0	0	0	0	0	0	0	0	0	0
**H24**	1	0	0	0	0	0	0	0	0	0	0

**H25**	1	0	0	0	0	0	0	0	0	0	0
**H26**	1	0	0	0	0	0	0	0	0	0	0
**H27**	1	0	0	0	0	0	0	0	0	0	0
**H28**	1	0	0	0	0	0	0	0	0	0	0
**H29**	1	0	0	0	0	0	0	0	0	0	0
**H30**	1	0	0	0	0	0	0	0	0	0	0
**H31**	1	0	0	0	0	0	0	0	0	0	0
**H32**	1	0	0	0	0	0	0	0	0	0	0
**H33**	1	0	0	0	0	0	0	0	0	0	0
**H34**	1	0	0	0	0	0	0	0	0	0	0
**H35**	1	0	0	0	0	0	0	0	0	0	0

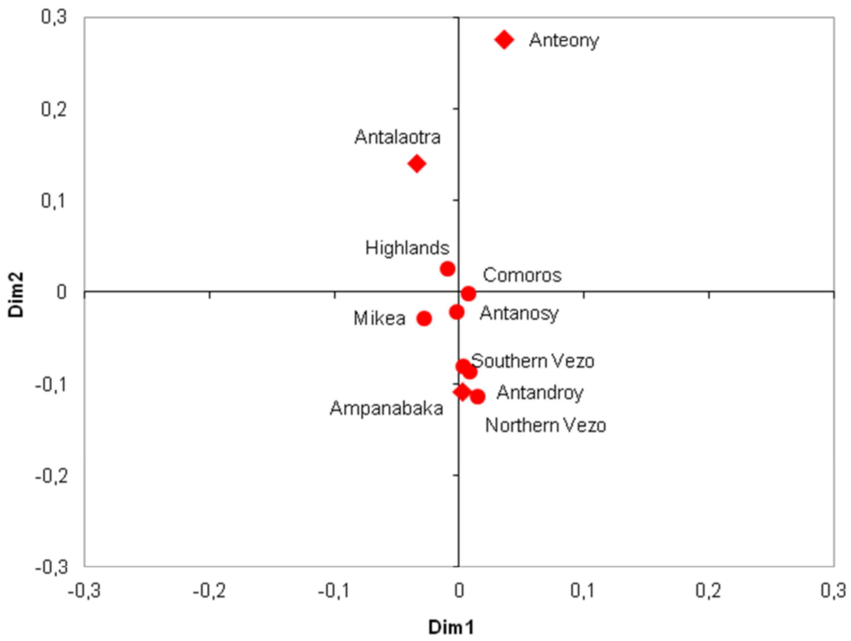

Figure S1. MDS plot of F_{ST} between Malagasy populations and the Comoros using NRY haplogroup frequencies (Kruskal stress: 0.172).

Table S12. Shared haplotypes between the Ampanabaka and the database using seven Y-STR markers

	Number of indiviuals	Number of haplotypes	Hap01	Hap02	Hap03	Hap04	Hap05	Hap06	Hap07	Hap08	Hap09	Hap10	Hap11	Hap12	Hap13	Hap14	Hap15	Hap16	Hap17	Hap18	Hap19	Hap20	Hap21	Hap22	Hap23	Hap24
			**E1b1a**	**E1b1a**	**O1a2**	**E1b1a**	**J2b**	**E1b1a**	**E1b1a**	**O1a2**	**E1b1a**	**E1b1a**	**E1b1a**	**E1b1a**	**O1a2**	**O1a2**	**B2**	**E1b1a**	**E1b1a**	**O1a2**	**O1a2**	**E1b1a**	**E2b**	**E1b1a**	**B2**	**E1b1b**
**Ethiopia Omo valley**	67	36	0	0	0	0	1	0	0	0	0	0	0	0	0	0	0	0	0	0	0	0	0	0	0	0
**Kenya Massai**	55	40	0	1	0	0	1	0	0	0	0	1	0	0	0	0	0	0	1	0	0	0	0	0	0	0
**Equatorial Guinea**	101	63	1	1	0	0	0	1	0	0	1	1	1	1	0	0	0	0	1	0	0	0	0	0	0	0
**Gabon**	828	239	1	1	0	0	0	1	0	0	1	1	1	1	0	0	1	1	1	0	0	1	1	1	0	0
**Benin**	78	51	1	1	0	0	0	1	0	0	1	1	0	1	0	0	0	0	1	0	0	0	0	0	0	0
**Ivory coast**	90	50	1	1	0	0	0	0	0	0	1	1	1	0	0	0	0	0	0	0	0	0	1	0	0	0
**African central Republic**	165	74	1	1	0	0	0	1	0	0	0	1	1	1	0	0	0	1	1	0	0	0	0	0	0	0
**Guinea Bissau**	161	106	1	1	0	0	0	1	0	0	1	1	1	1	0	0	0	1	1	0	0	0	0	0	0	0
**Burkina Faso**	323	142	1	1	0	1	0	0	0	0	1	1	1	1	0	0	0	1	1	0	0	0	0	0	0	0
**Mozambique**	154	56	1	1	0	0	0	1	0	0	1	1	1	0	0	0	1	1	1	0	0	1	1	1	0	0
**Angola Nyaneka**	75	40	1	0	0	0	0	1	0	0	1	1	1	0	0	0	1	1	1	0	0	0	0	1	0	0
**Angola Ovimbudu**	96	43	1	1	0	0	0	1	0	0	0	1	1	0	0	0	1	0	1	0	0	0	0	0	0	0
**Angola various**	65	39	1	0	0	0	0	1	0	0	1	1	1	0	0	0	0	0	1	0	0	0	0	1	0	0
**Somalia Danish various**	201	59	0	0	0	0	0	0	0	0	0	0	0	0	0	0	0	0	1	0	0	0	0	0	0	0
**Tanzania Sandawa**	66	33	1	1	0	0	0	0	0	0	0	1	0	0	0	0	0	0	0	0	0	0	0	0	0	1
**Tanzania various**	132	67	1	1	0	0	0	1	0	0	1	1	1	1	0	0	0	0	1	0	0	0	0	0	0	0
**Uganda**	118	52	0	0	0	0	0	0	0	0	1	1	0	0	0	0	0	0	0	0	0	0	0	0	0	0
**Eastern Zambia, Eastern Bantus**	88	49	1	1	0	0	0	1	0	0	1	1	1	0	0	0	0	1	1	0	0	0	0	0	0	0
**Western Zambia, Eastern Bantus**	263	109	1	1	1	0	0	1	0	0	1	1	1	1	0	0	0	1	1	0	0	0	0	0	0	0
**Western Zambia, Western Bantus**	192	82	1	1	0	1	0	1	0	0	0	1	1	1	0	0	0	1	1	0	0	0	0	1	0	0

**Tunisia**	117	80	0	0	0	0	0	0	0	0	0	0	0	0	0	0	0	0	0	0	0	0	0	0	0	0
**Tunisia Berbers various**	66	25	0	0	0	0	0	0	0	0	0	0	0	0	0	0	0	0	0	0	0	0	0	0	0	0
**Egypt various**	92	64	0	0	0	0	0	0	0	0	0	0	0	0	0	0	0	0	0	0	0	0	0	0	0	0
**Algeria**	100	54	0	1	0	0	0	0	0	0	0	1	0	0	0	0	0	0	0	0	0	0	0	0	0	1
**Lybia**	63	52	0	0	0	0	0	0	0	0	0	0	0	0	0	0	0	0	0	0	0	0	0	0	0	0
**Morocco Moroccan**	515	212	1	1	0	1	0	1	0	0	1	1	0	0	0	0	0	0	0	0	0	0	0	0	0	0
**Morocco Arabs various**	104	61	0	0	0	0	0	0	0	0	0	1	0	0	0	0	0	0	0	0	0	0	0	0	0	0
**Mozabites**	68	22	0	0	0	0	0	0	0	0	0	0	0	0	0	0	0	0	0	0	0	0	0	0	0	0
**Tunisia Sfax various**	105	64	0	0	0	0	0	0	0	0	0	0	0	0	0	0	0	0	0	0	0	0	0	0	0	1
**Namibia**	54	32	1	0	0	0	0	1	0	0	0	1	1	0	0	0	0	1	1	0	0	0	0	0	0	0
**Iranian Iran**	128	93	0	0	0	0	0	0	0	0	0	0	0	0	0	0	0	0	0	0	0	0	0	0	0	0
**Druzes Israel**	234	89	0	0	0	0	0	0	0	0	0	0	0	0	0	0	0	0	1	0	0	0	0	0	0	0
**Lebanon**	650	298	0	0	0	0	0	0	0	0	0	1	0	0	0	0	0	0	0	1	0	0	0	0	0	1
**Palestine**	364	181	0	0	0	0	0	0	0	0	0	0	0	1	0	0	0	0	0	0	0	0	0	0	0	0
**Kuwait**	148	45	0	0	0	0	0	0	0	0	0	0	0	0	0	0	0	1	0	0	0	0	0	0	0	0
**Oman**	99	68	1	0	0	0	0	0	0	0	0	1	0	0	0	0	0	1	1	0	0	0	0	0	0	0
**Saudi Arabia**	106	60	0	0	0	0	0	0	0	0	0	0	0	0	0	0	0	0	0	0	0	1	0	0	0	0
**Yemen**	104	52	0	0	0	0	0	0	0	0	0	1	0	0	0	0	0	0	0	0	0	0	0	0	0	1
**Dubai**	217	146	1	1	0	0	0	1	0	0	0	1	0	0	0	0	0	0	1	0	0	0	1	0	0	0
**Iran**	104	91	0	1	0	0	0	0	0	0	0	0	0	1	0	0	0	0	1	0	0	0	0	0	0	0
**Cyprus**	163	97	0	0	0	0	0	0	0	0	0	0	0	0	0	0	0	0	0	0	0	0	0	0	0	1
**Syria Syrians**	161	124	0	0	0	0	0	0	0	0	0	0	0	0	0	0	0	0	0	0	0	0	0	0	0	1
**Turkey various**	140	78	0	0	0	0	0	0	0	0	0	1	0	0	0	0	0	0	0	0	0	0	0	0	0	0
**Turkey Anatolia**	520	330	0	0	0	0	0	0	0	0	0	1	0	0	0	0	0	0	0	0	0	0	0	0	0	1
**Bangladesh**	284	197	0	0	0	0	0	0	0	0	0	0	0	0	0	0	0	0	0	0	0	0	0	0	0	0
**Saraswat Brahmin**	122	92	0	0	0	0	0	0	0	0	0	0	0	0	0	0	0	0	0	0	0	0	0	0	0	0
**India Tamil Nadu**	152	108	0	0	0	0	0	0	0	0	0	0	0	0	0	0	0	0	0	0	0	0	0	0	0	0
**Jat Sikhs**	80	47	0	0	0	0	0	0	0	0	0	0	0	0	0	0	0	0	0	0	0	0	0	0	0	0
**Northern India**	78	56	0	0	0	0	0	0	0	0	0	0	0	0	0	0	0	0	0	0	0	0	0	0	0	0
**Eastern India**	127	77	0	0	0	0	0	0	0	0	0	0	0	0	0	0	0	0	0	0	0	0	0	0	0	0

**Southern India**	295	155	0	0	0	0	0	0	0	0	0	0	0	0	0	0	0	0	1	0	0	0	0	0	0	0
**Central India**	69	27	0	0	0	0	0	0	0	0	0	0	0	0	0	0	0	0	0	0	0	0	0	0	0	0
**Western India**	59	49	0	0	0	0	1	0	0	0	0	0	0	0	0	0	0	0	0	0	0	0	0	0	0	0
**Mahadeokoli**	50	39	0	0	0	0	0	0	0	0	0	0	1	0	0	0	0	0	0	0	0	0	0	0	0	0
**Thakar**	48	28	0	0	0	0	0	0	0	0	0	0	0	0	0	0	0	0	0	0	0	0	0	0	0	0
**Pakistan**	177	119	0	0	0	0	0	1	0	0	0	0	0	0	0	0	0	0	0	0	0	0	0	0	0	0
**Sri-Lanka**	207	162	0	0	0	0	0	0	0	0	0	0	0	0	0	0	0	0	0	0	0	0	0	0	0	0
**France**	57	35	0	0	0	0	0	0	0	0	0	0	0	0	0	0	0	0	0	0	0	0	0	0	0	0
**- Northern Portugal**	60	48	0	0	0	0	0	0	0	0	0	0	0	0	0	0	0	0	0	0	0	0	0	0	0	0
**- Southern Portugal**	78	66	0	0	0	0	0	0	0	0	0	0	0	0	0	0	0	0	0	0	0	0	0	0	0	0
**EasternAndalusia**	95	60	0	0	0	0	0	0	0	0	0	0	0	0	0	0	0	0	0	0	0	0	0	0	0	0
**Galicia**	89	65	0	0	0	0	0	0	0	0	0	0	0	0	0	0	0	0	0	0	0	0	0	0	0	0
**Spain - Northern Castilla**	130	85	0	0	0	0	0	0	0	0	0	0	0	0	0	0	0	0	0	0	0	0	0	0	0	0
**Portuguese Jew**	86	49	0	0	0	0	0	0	0	0	0	0	0	0	0	0	0	0	0	0	0	0	0	0	0	0
**Phillipines**	76	64	0	0	1	0	0	0	0	0	0	0	0	0	1	1	0	0	0	0	0	0	0	0	0	0
**Malay Singapore**	180	134	0	0	1	0	0	0	0	0	0	0	0	0	0	1	0	0	0	0	0	0	0	0	0	0
**Malay Malaysia**	334	245	0	0	1	0	0	0	0	1	0	0	0	0	1	1	0	0	0	0	0	0	0	0	0	0
**Iban**	105	57	0	0	0	0	0	0	0	0	0	0	0	0	0	1	0	0	0	0	0	0	0	0	0	0
**Bidayuh**	113	45	0	0	0	0	0	0	0	0	0	0	0	0	0	0	0	0	0	0	0	0	0	0	0	0
**Melanau**	104	39	0	0	1	0	0	0	0	0	0	0	0	0	0	1	0	0	0	0	0	0	0	0	0	0
**Timor**	113	81	0	0	1	0	0	0	0	0	0	0	1	0	1	0	0	0	0	0	0	0	0	0	0	0
**Flores**	385	126	0	0	0	0	0	0	0	0	0	0	0	0	0	0	0	0	0	1	0	0	0	0	0	1
**Lembata**	89	40	0	0	0	0	0	0	0	0	0	1	1	0	0	0	0	1	0	1	0	0	0	0	0	1
**Sumba**	349	73	0	0	1	0	0	0	0	0	0	0	0	0	0	1	0	0	0	1	0	0	0	0	0	0
**Vietnam**	113	84	0	0	0	0	0	0	0	0	0	0	0	0	0	0	0	0	0	0	0	0	0	0	0	0
**Bali**	632	171	0	0	1	0	0	0	0	0	0	0	0	0	1	1	0	0	0	0	0	0	0	0	0	0
**Borneo**	85	35	0	0	1	0	0	0	0	0	0	0	0	0	1	1	0	0	0	0	0	0	0	0	0	0
**Java**	61	37	0	0	1	0	0	0	0	0	0	0	0	0	0	0	0	0	0	0	0	0	0	0	0	0
**Mentawai**	73	18	0	0	0	0	0	0	0	1	0	0	0	0	0	1	0	0	0	0	0	0	0	0	0	0
**Nias**	60	15	0	0	1	0	0	0	0	1	0	0	0	0	0	1	0	0	0	0	1	0	0	0	0	0

**Oceania various**	66	40	0	0	0	0	0	0	0	0	0	0	0	0	0	0	0	0	0	0	0	0	0	0	0	0
**Papouasie New Guinea**	47	42	0	0	0	0	0	0	0	0	1	0	0	0	0	0	0	0	0	0	0	0	0	0	0	0
**Tahiti**	24	14	0	0	0	0	0	0	0	0	0	0	0	0	0	0	0	0	0	0	0	0	0	0	0	0
**Vanuatu**	44	32	0	0	0	0	0	0	0	0	0	0	0	0	0	0	0	0	0	0	0	0	0	0	0	0
**Thailand**	41	39	0	0	0	0	0	0	0	0	0	0	0	0	0	1	0	0	0	0	0	0	0	0	0	0

Table S13. Shared haplotypes between the Antalaotra and the database, using seven Y-STR markers

	**Number of indiviuals**	**Number of haplotypes**	**Hap.01**	**Hap.02**	**Hap.03**	**Hap.04**	**Hap.05**	**Hap.06**	**Hap.07**	**Hap.08**	**Hap.09**	**Hap.10**	**Hap.11**	**Hap.12**	**Hap.13**	**Hap.14**	**Hap.15**	**Hap.16**	**Hap.17**	**Hap.18**	**Hap.19**	**Hap.20**	**Hap.21**
			**E1b1a**	**O1a2**	**J2b**	**E1b1a**	**O1a2**	**O1a2**	**T1**	**J1**	**T1**	**O2a1**	**O2a1**	**E2b**	**O2a1**	**O2a1**	**J2b**	**E2b**	**J2b**	**O2a1**	**O2a1**	**R1a1**	**E1b1a**
**Ethiopia Omo valley**	67	36	0	0	1	0	0	0	0	0	0	0	0	0	0	0	0	0	0	0	0	0	0
**Kenya Massai**	55	40	1	0	1	0	0	0	0	0	0	0	0	0	0	0	0	0	0	0	0	0	0
**Equatorial Guinea**	101	63	1	0	0	1	0	0	0	0	0	0	0	0	0	0	0	0	0	0	0	0	1
**Gabon**	828	239	1	0	0	1	0	0	0	0	0	0	1	1	0	0	0	1	0	0	0	0	1
**Benin**	78	51	1	0	0	1	0	0	0	0	0	0	0	0	0	0	0	0	0	0	0	0	1
**Ivory coast**	90	50	1	0	0	0	0	0	0	0	0	0	0	0	0	0	0	0	0	0	0	0	1
**African central Republic**	165	74	1	0	0	1	0	0	0	0	0	0	0	0	0	0	0	0	0	0	0	0	1
**Guinea Bissau**	161	106	1	0	0	1	0	0	0	0	0	0	0	0	0	0	0	0	0	0	0	0	1
**Burkina Faso**	323	142	1	0	0	0	0	0	0	0	0	0	0	0	0	0	0	0	0	0	0	0	1
**Mozambique**	154	56	1	0	0	1	0	0	0	0	0	0	0	1	0	0	0	0	0	0	0	0	1
**Angola Nyaneka**	75	40	0	0	0	1	0	0	0	0	0	0	0	0	0	0	0	1	0	0	0	0	1
**Angola Ovimbudu**	96	43	1	0	0	1	0	0	0	0	0	0	0	0	0	0	0	0	0	0	0	0	1
**Angola various**	65	39	0	0	0	1	0	0	0	0	0	0	0	1	0	0	0	0	0	0	0	0	1
**Somalia Danish various**	201	59	0	0	0	0	0	0	0	0	0	0	0	0	0	0	0	0	0	0	0	0	0
**Tanzania Sandawa**	66	33	1	0	0	0	0	0	0	0	0	0	0	0	0	0	0	0	0	0	0	0	0

**Tanzania various**	132	67	1	0	0	1	0	0	0	0	0	0	0	0	0	0	1	0	0	0	0	0	1
**Uganda**	118	52	0	0	0	0	0	0	0	0	0	0	0	0	0	0	0	0	1	0	0	0	0
**Eastern Zambia, Eastern Bantus**	88	49	1	0	0	1	0	0	0	0	0	0	0	1	0	0	0	0	0	0	0	0	1
**Western Zambia, Eastern Bantus**	263	109	1	1	0	1	0	0	0	0	0	0	0	0	0	0	0	0	0	0	0	0	1
**Western Zambia, Western Bantus**	192	82	1	0	0	1	0	0	0	0	0	0	0	1	0	0	0	0	0	0	0	0	1
**Tunisia**	117	80	0	0	0	0	0	0	0	0	0	0	0	0	0	0	0	0	0	0	0	0	0
**Tunisia Berbers various**	66	25	0	0	0	0	0	0	0	0	0	0	0	0	0	0	0	0	0	0	0	0	0
**Egypt various**	92	64	0	0	0	0	0	0	0	0	0	0	0	0	0	0	0	0	0	0	0	0	0
**Algeria**	100	54	1	0	0	0	0	0	0	1	0	0	0	0	0	0	0	0	0	0	0	1	1
**Lybia**	63	52	0	0	0	0	0	0	0	0	0	0	0	0	0	0	0	0	0	0	0	0	0
**Morocco Moroccan**	515	212	1	0	0	1	0	0	0	1	0	0	0	0	0	0	0	0	0	0	0	0	1
**Morocco Arabs various**	104	61	0	0	0	0	0	0	0	0	0	0	0	0	0	0	0	0	0	0	0	0	0
**Mozabites**	68	22	0	0	0	0	0	0	0	0	0	0	0	0	0	0	0	0	0	0	0	0	0
**Tunisia Sfax various**	105	64	0	0	0	0	0	0	0	0	0	0	0	0	0	0	0	0	0	0	0	0	0
**Namibia**	54	32	0	0	0	1	0	0	0	0	0	0	0	1	0	0	0	0	0	0	0	0	1
**Iranian Iran**	128	93	0	0	0	0	0	0	0	0	0	0	0	0	0	0	0	0	0	0	0	0	0
**Druzes Israel**	234	89	0	0	0	0	0	0	1	0	0	0	0	0	0	0	0	0	0	0	0	0	0
**Lebanon**	650	298	0	0	0	0	0	0	1	1	0	0	0	0	0	0	0	0	0	0	0	0	0
**Palestine**	364	181	0	0	0	0	0	0	1	1	0	0	0	1	0	0	0	0	0	0	0	0	1
**Kuwait**	148	45	0	0	0	0	0	0	0	0	0	0	0	0	0	0	0	0	0	0	0	0	1
**Oman**	99	68	0	0	0	0	0	0	0	0	0	0	0	1	0	0	0	0	0	0	0	1	0
**Saudi Arabia**	106	60	0	0	0	0	0	0	0	0	0	0	0	0	0	0	1	0	0	0	0	0	0
**Yemen**	104	52	0	0	0	0	0	0	0	0	0	0	0	0	0	0	0	0	0	0	0	0	0
**Dubai**	217	146	1	0	0	1	0	0	0	1	0	0	0	1	0	0	0	0	0	0	0	0	1
**Iran**	104	91	1	0	0	0	0	0	0	0	0	0	0	0	0	0	0	0	0	0	0	0	1
**Cyprus**	163	97	0	0	0	0	0	0	0	1	0	0	0	0	0	0	0	0	0	0	0	0	0
**Syria Syrians**	161	124	0	0	0	0	0	0	0	1	0	0	0	0	0	0	1	0	0	0	0	1	0
**Turkey various**	140	78	0	0	0	0	0	0	0	0	0	0	0	0	0	0	0	0	0	0	0	1	0
**Turkey Anatolia**	520	330	0	0	0	0	0	0	0	1	0	0	0	0	0	0	1	0	0	0	0	1	0
**Bangladesh**	284	197	0	0	0	0	0	0	0	0	0	1	0	0	1	0	0	0	0	1	0	1	0
**Saraswat Brahmin**	122	92	0	0	0	0	0	0	0	0	0	0	0	0	0	0	0	0	0	0	0	1	0
**India Tamil Nadu**	152	108	0	0	0	0	0	0	0	0	0	0	0	0	0	0	1	0	0	0	0	1	0
**Jat Sikhs**	80	47	0	0	0	0	0	0	0	0	0	0	0	0	0	0	0	0	0	0	0	1	0
**Northern India**	78	56	0	0	0	0	0	0	0	1	0	0	0	0	0	0	0	0	0	0	0	0	0

**Eastern India**	127	77	0	0	0	0	0	0	0	1	0	0	0	0	0	0	0	0	0	0	0	0	0
**Southern India**	295	155	0	0	0	0	0	0	0	0	0	1	1	0	0	0	0	0	0	0	0	0	0
**Central India**	69	27	0	0	0	0	0	0	0	0	0	1	0	0	0	0	0	0	0	0	0	0	0
**Western India**	59	49	0	0	1	0	0	0	0	0	0	0	0	0	0	0	0	0	0	0	0	1	0
**Mahadeokoli**	50	39	0	0	0	0	0	0	0	0	0	0	0	0	0	0	0	0	0	0	0	0	0
**Thakar**	48	28	0	0	0	0	0	0	0	0	0	0	0	0	0	0	0	0	0	0	0	0	0
**Pakistan**	177	119	0	0	0	1	0	0	0	1	0	0	0	0	0	0	0	0	0	0	0	0	0
**Sri-Lanka**	207	162	0	0	0	0	0	0	0	0	0	0	0	0	0	0	0	0	0	0	0	1	0
**France**	57	35	0	0	0	0	0	0	0	0	0	0	1	0	0	0	0	0	0	0	0	0	0
**Northern Portugal**	60	48	0	0	0	0	0	0	0	0	0	0	0	0	0	0	0	0	0	0	0	0	0
**Southern Portugal**	78	66	0	0	0	0	0	0	0	0	0	0	0	0	0	0	0	0	0	0	0	0	0
**Eastern Andalusia**	95	60	0	0	0	0	0	0	0	1	0	0	0	0	0	0	0	0	0	0	0	0	0
**Galicia**	89	65	0	0	0	0	0	0	0	0	0	0	0	0	0	0	0	0	0	0	0	0	0
**Spain - Northern Castilla**	130	85	0	0	0	0	0	0	0	0	0	0	1	0	1	1	0	0	0	0	0	0	0
**Portuguese Jew**	86	49	0	0	0	0	0	0	0	1	0	0	0	0	0	0	0	0	0	0	0	0	0
**Phillipines**	76	64	0	1	0	0	1	1	0	0	0	0	0	0	0	0	0	0	0	0	0	0	0
**Malay Singapore**	180	134	0	1	0	0	0	1	0	0	0	1	0	0	1	0	0	0	0	0	0	0	0
**Malay Malaysia**	334	245	0	1	0	0	1	1	0	0	0	1	1	0	1	0	0	0	0	1	0	1	0
**Iban**	105	57	0	0	0	0	0	1	0	0	0	0	0	0	1	0	0	0	0	0	1	0	0
**Bidayuh**	113	45	0	0	0	0	0	0	0	0	0	0	0	0	1	0	0	0	0	0	0	0	0
**Melanau**	104	39	0	1	0	0	0	1	0	0	0	1	0	0	1	0	0	0	0	0	0	0	0
**Timor**	113	81	0	1	0	0	1	0	0	0	0	0	1	0	0	0	0	0	0	0	0	0	1
**Flores**	385	126	0	0	0	0	0	0	0	0	0	0	0	0	0	0	0	0	0	0	0	0	1
**Lembata**	89	40	0	0	0	0	0	0	0	0	0	0	0	0	0	0	0	0	0	0	0	0	0
**Sumba**	349	73	0	1	0	0	0	1	1	0	0	0	0	0	0	0	0	0	0	0	0	0	0
**Vietnam**	113	84	0	0	0	0	0	0	0	0	1	1	0	0	0	0	0	0	0	0	0	0	0
**Bali**	632	171	0	1	0	0	1	1	0	0	0	1	1	0	1	0	0	0	0	0	1	0	0
**Borneo**	85	35	0	1	0	0	1	1	0	0	0	1	0	0	1	0	0	0	0	0	0	0	0
**Java**	61	37	0	1	0	0	0	0	0	0	0	1	1	0	1	0	0	0	0	0	0	0	0
**Mentawai**	73	18	0	0	0	0	0	1	0	0	0	0	0	0	0	0	0	0	0	0	0	0	0
**Nias**	60	15	0	1	0	0	0	1	0	0	0	0	0	0	0	0	0	0	0	0	0	0	0
**Oceania various**	66	40	0	0	0	0	0	0	0	0	0	0	0	0	0	0	0	0	0	0	0	0	0
**Papouasie New Guinea**	47	42	0	0	0	0	0	0	0	0	0	0	0	0	0	0	0	0	0	0	0	0	1
**Tahiti**	24	14	0	0	0	0	0	0	0	0	0	0	0	0	0	0	0	0	0	0	0	0	0
**Vanuatu**	44	32	0	0	0	0	0	0	0	0	0	0	0	0	0	0	0	0	0	0	0	0	0
**Thailand**	41	39	0	0	0	0	0	1	0	0	0	0	0	0	1	0	0	0	0	1	0	0	0

Table S14. Shared haplotypes between the Anteony and the database using seven Y-STR markers

	Number of individuals	Number of haplotypes	Hap.01	Hap.02	Hap.03	Hap.04	Hap.05	Hap.06	Hap.07	Hap.08	Hap.09	Hap.10	Hap.11	Hap.12
			E1b1a	E1b1a	E1b1a	E1b1a	T1	J1	T1	T1	T1	E1b1b	O2a1	T1
**Ethiopia Omo valley**	**67**	36	0	0	0	0	0	0	0	0	0	1	0	0
**Kenya Massai**	**55**	40	0	1	0	0	0	0	0	0	0	0	0	0
**Equatorial Guinea**	**101**	63	1	1	1	0	0	0	0	0	0	0	0	0
**Gabon**	**828**	239	1	1	1	0	0	0	0	0	0	0	0	0
**Benin**	**78**	51	1	1	1	0	0	0	0	0	0	0	0	0
**Ivory coast**	**90**	50	1	1	0	0	0	0	0	0	0	0	0	0
**African central Republic**	**165**	74	1	1	1	0	0	0	0	0	0	0	0	0
**Guinea Bissau**	**161**	106	1	1	1	0	0	0	0	0	0	1	0	0
**Burkina Faso**	**323**	142	1	1	0	0	0	0	0	0	0	1	0	0
**Mozambique**	**154**	56	1	1	1	0	0	0	0	0	0	0	0	0
**Angola Nyaneka**	**75**	40	1	0	1	0	0	0	0	0	0	0	0	0
**Angola Ovimbudu**	**96**	43	1	1	1	0	0	0	0	0	0	0	0	0
**Angola various**	**65**	39	1	0	1	0	0	0	0	0	0	0	0	1
**Somalia Danish various**	**201**	59	0	0	0	0	0	0	0	0	0	0	0	0
**Tanzania Sandawa**	**66**	33	1	1	0	0	0	0	0	0	0	0	0	0
**Tanzania various**	**132**	67	1	1	1	0	0	0	0	0	0	0	0	0
**Uganda**	**118**	52	0	0	0	0	0	0	0	0	0	1	0	0
**Eastern Zambia, Eastern Bantus**	**88**	49	1	1	1	0	0	0	0	0	0	0	0	0
**Western Zambia, Eastern Bantus**	**263**	109	1	1	1	0	0	0	0	0	0	0	0	0
**Western Zambia, Western Bantusn**	**192**	82	1	1	1	0	0	0	0	0	0	0	0	0
**Tunisia**	**117**	80	0	0	0	0	0	0	0	0	0	0	0	0

**Tunisia Berbers various**	**66**	25	0	0	0	0	0	0	0	0	0	0	0	0
**Egypt various**	**92**	64	0	0	0	0	0	0	0	0	0	0	0	0
**Algeria**	**100**	54	0	1	0	0	0	1	0	0	0	0	0	0
**Lybia**	**63**	52	0	0	0	0	0	0	0	0	0	0	0	0
**Morocco Moroccan**	**515**	212	1	1	1	0	0	1	0	0	0	0	0	0
**Morocco Arabs various**	**104**	61	0	0	0	0	0	0	0	0	0	1	0	0
**Mozabites**	**68**	22	0	0	0	0	0	0	0	0	0	0	0	0
**Tunisia Sfax various**	**105**	64	0	0	0	0	0	0	0	0	0	0	0	0
**Namibia**	**54**	32	1	0	1	0	0	0	0	0	0	0	0	0
**Iranian Iran**	**128**	93	0	0	0	0	0	0	0	0	0	0	0	0
**Druzes Israel**	**234**	89	0	0	0	0	1	0	0	0	0	1	0	0
**Lebanon**	**650**	298	0	0	0	0	1	1	0	1	0	1	0	0
**Palestine**	**364**	181	0	0	0	0	1	1	0	0	0	1	0	0
**Kuwait**	**148**	45	0	0	0	0	0	0	0	0	0	0	0	0
**Oman**	**99**	68	1	0	0	0	0	0	0	0	0	0	0	0
**Saudi Arabia**	**106**	60	0	0	0	0	0	0	0	0	0	0	0	0
**Yemen**	**104**	52	0	0	0	0	0	0	0	0	0	1	0	0
**Dubai**	**217**	146	1	1	1	0	0	1	0	0	0	1	0	0
**Iran**	**104**	91	0	1	0	0	0	0	0	0	0	0	0	0
**Cyprus**	**163**	97	0	0	0	0	0	1	0	0	0	1	0	0
**Syria Syrians**	**161**	124	0	0	0	0	0	1	0	0	0	0	0	0
**Turkey various**	**140**	78	0	0	0	0	0	0	0	0	0	0	0	0
**Turkey Anatolia**	**520**	330	0	0	0	0	0	1	0	1	0	1	0	1
**Bangladesh**	**284**	197	0	0	0	0	0	0	0	0	0	0	1	0
**Saraswat Brahmin**	**122**	92	0	0	0	0	0	0	0	0	0	0	0	0
**India Tamil Nadu**	**152**	108	0	0	0	0	0	0	0	0	0	0	0	0
**Jat Sikhs**	**80**	47	0	0	0	0	0	0	0	0	0	0	0	0
**Northern India**	**78**	56	0	0	0	0	0	1	0	0	0	0	0	0
**Eastern Indian**	**127**	77	0	0	0	0	0	1	0	0	0	0	0	0

**Southern India**	**295**	155	0	0	0	0	0	0	0	0	0	0	1	0
**Central India**	**69**	27	0	0	0	0	0	0	0	0	0	0	1	0
**Western India**	**59**	49	0	0	0	0	0	0	0	0	0	0	0	0
**Mahadeokoli**	**50**	39	0	0	0	0	0	0	0	0	0	0	0	0
**Thakar**	**48**	28	0	0	0	0	0	0	0	0	0	0	0	0
**Pakistan**	**177**	119	0	0	1	0	0	1	0	0	0	0	0	0
**Sri-Lanka**	**207**	162	0	0	0	0	0	0	0	0	0	0	0	0
**France**	**57**	35	0	0	0	0	0	0	0	0	0	0	0	0
**Northern Portugal**	**60**	48	0	0	0	0	0	0	0	0	0	1	0	0
**Southern Portugal**	**78**	66	0	0	0	0	0	0	0	0	0	0	0	0
**Eastern Andalusia**	**95**	60	0	0	0	0	0	1	0	0	0	0	0	0
**Galicia**	**89**	65	0	0	0	0	0	0	0	0	0	1	0	0
**Spain - Northern Castilla**	**130**	85	0	0	0	0	0	0	0	0	0	1	0	0
**Portuguese Jew**	**86**	49	0	0	0	0	0	1	0	1	0	1	0	0
**Phillipines**	**76**	64	0	0	0	0	0	0	0	0	0	0	0	0
**Malay Singapore**	**180**	134	0	0	0	0	0	0	0	0	0	0	1	0
**Malay Malaysia**	**334**	245	0	0	0	0	0	0	0	0	0	0	1	0
**Iban**	**105**	57	0	0	0	0	0	0	0	0	0	0	0	0
**Bidayuh**	**113**	45	0	0	0	0	0	0	0	0	0	0	0	0
**Melanau**	**104**	39	0	0	0	0	0	0	0	0	0	0	1	0
**Timor**	**113**	81	0	0	0	0	0	0	0	1	0	0	0	0
**Flores**	**385**	126	0	0	0	0	0	0	0	0	0	0	0	1
**Lembata**	**89**	40	0	0	0	0	0	0	0	1	0	0	0	0
**Sumba**	**349**	73	0	0	0	0	1	0	0	1	0	0	0	0
**Vietnam**	**113**	84	0	0	0	0	0	0	1	0	0	0	1	0
**Bali**	**632**	171	0	0	0	0	0	0	0	0	0	0	1	0
**Borneo**	**85**	35	0	0	0	0	0	0	0	0	0	0	1	0
**Java**	**61**	37	0	0	0	0	0	0	0	0	0	0	1	0

**Mentawai**	**73**	18	0	0	0	0	0	0	0	0	0	0	0	0
**Nias**	**60**	15	0	0	0	0	0	0	0	0	0	0	0	0
**Oceania various**	**66**	40	0	0	0	0	0	0	0	0	0	0	0	0
**Papouasie NG**	**47**	42	0	0	0	0	0	0	0	1	0	0	0	0
**Tahiti**	**24**	14	0	0	0	0	0	0	0	0	0	0	0	0
**Vanuatu**	**44**	32	0	0	0	0	0	0	0	0	0	0	0	0
**Thailand**	**41**	39	0	0	0	0	0	0	0	0	0	0	0	0

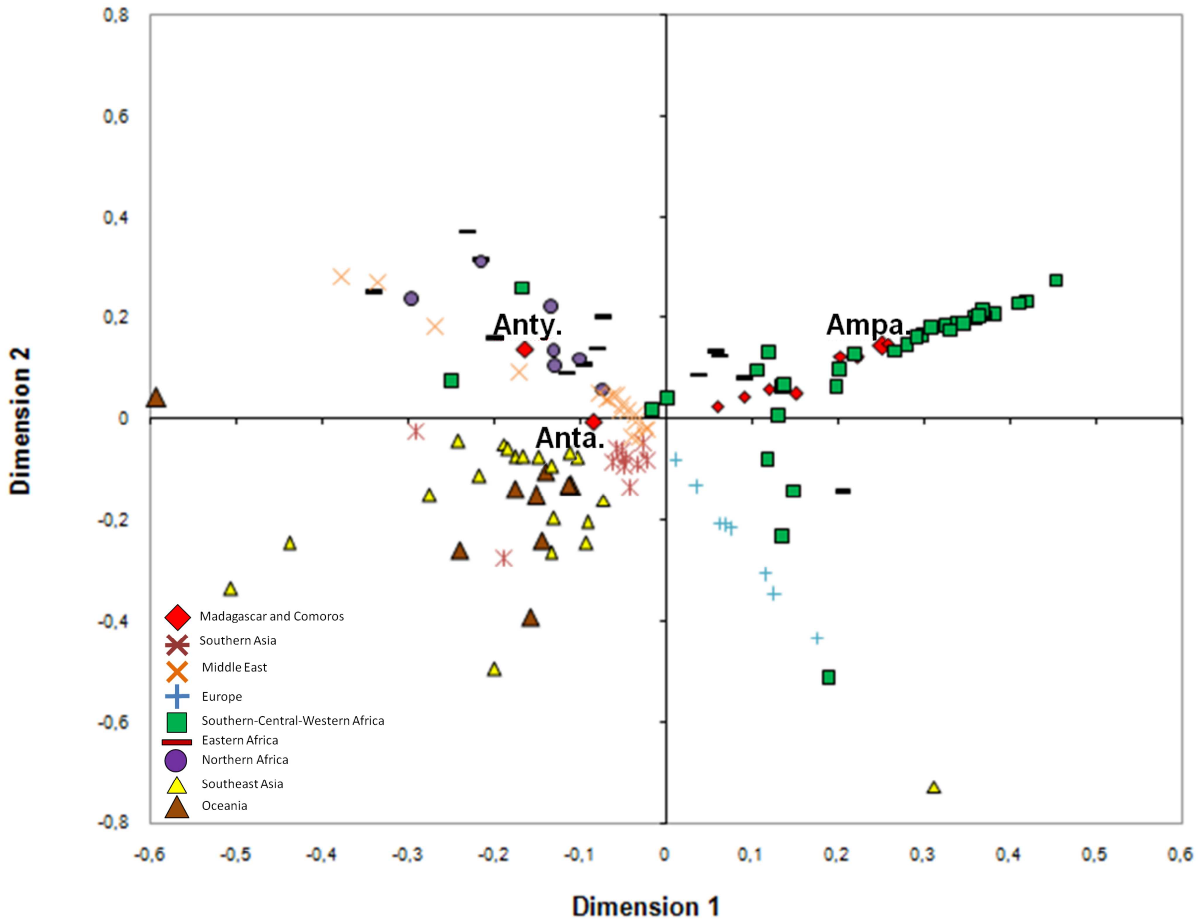

Figure S2. MDS plot of F_{ST} computed from Y haplogroup frequencies between the Antemoro and populations from various geographic regions (Kruskal stress =0.227). Anty: Anteony, Anta: Antalaotra, Ampa: Ampanabaka.

Table S15. Population pairwise FST values based on HVI in our three Antemoro groups

	**Ampanabaka**		**Anteony**		**Antalaotra**	
	$\mathbf{F}_{ST}$	**Significativity seuil 5%**	$\mathbf{F}_{ST}$	**Significativity seuil 5%**	$\mathbf{F}_{ST}$	**Significativity seuil 5%**
**Ampanabaka**	*	*				
**Anteony**	0.00308	-	*	*		
**Antalaotra**	-0.01249	-	-0.00327	-	*	*
**Highlands Madagascar**	0.02868	+	0.03267	+	0.02209	-
**Antanosy**	-0.01371	-	0.01076	-	-0.00457	-
**Antandroy**	0.00390	-	0.01923	+	0.00963	-
**Mikea**	0.02314	+	0.01603	+	0.02761	+
**Vezo - Northern Tulear**	0.02224	+	0.00432	-	0.01967	+
**Vezo - Southern Tulear**	-0.00476	-	0.03348	+	0.01146	-
**Angola Nyaneka**	0.11328	+	0.07044	+	0.11840	+
**Angola Ovimbudu**	0.09680	+	0.05456	+	0.10350	+
**Fon Benin**	0.08136	+	0.04245	+	0.08817	+
**Cabinda Angola**	0.09690	+	0.06021	+	0.09970	+
**Cabo verde**	0.08850	+	0.05309	+	0.09153	+
**Cameroon Ngumba**	0.16808	+	0.12283	+	0.17060	+
**Cameroon Pygmies Baka**	0.55022	+	0.50240	+	0.55342	+
**Cameroon Pygmies Bakola**	0.66479	+	0.62033	+	0.67106	+
**Guanche Canaries Islands**	0.15991	+	0.14576	+	0.14686	+
**Egypt Arabs**	0.07063	+	0.05093	+	0.07356	+
**Egypts Copts**	0.12162	+	0.11184	+	0.12323	+
**Gabon Fang**	0.14899	+	0.09860	+	0.15394	+
**Gabon Mitsogo**	0.09549	+	0.07485	+	0.09176	+
**Gabon Nzebi**	0.12772	+	0.08721	+	0.12803	+
**Guinea Bissau**	0.07790	+	0.04025	+	0.08038	+
**Ivory coast Ahizi**	0.06229	+	0.03867	+	0.06321	+
**Ivory coast Yacouba**	0.09363	+	0.04537	+	0.09984	+
**Kenya Swahili**	0.05018	+	0.02810	+	0.05026	+
**Mali Malinke**	0.09460	+	0.05130	+	0.09669	+
**Moors Mauritania**	0.09725	+	0.06210	+	0.09169	+
**Morocco Berbers**	0.09510	+	0.08424	+	0.08473	+
**Mozambique**	0.11165	+	0.06931	+	0.12438	+
**Rwanda Hutu**	0.09070	+	0.05299	+	0.09043	+
**Sao Tome**	0.10694	+	0.06191	+	0.11061	+
**Senegal Mandenka**	0.12783	+	0.08691	+	0.12581	+
**Senegal Wolof**	0.04823	+	0.02964	+	0.04262	+
**South Africa Kung**	0.24827	+	0.21230	+	0.25302	+
**Tanzania Hadza**	0.28846	+	0.21607	+	0.29385	+
**Tanzania Sandawe**	0.20552	+	0.15476	+	0.20437	+
**Zimbabwe**	0.09277	+	0.05355	+	0.09439	+
**Tunisia**	0.06120	+	0.04887	+	0.05491	+
**Algeria**	0.08023	+	0.06749	+	0.07702	+

**Sahawari**	0.06787	+	0.04662	+	0.05815	+
**Samaritans**	0.33822	+	0.32356	+	0.33873	+
**Ethiopia Amharic Various**	0.05723	+	0.03393	+	0.05769	+
**Sudan Nubia**	0.06086	+	0.02963	+	0.06248	+
**Tunisia Various**	0.10721	+	0.10270	+	0.10002	+
**Morocco Berbers Bourhiah**	0.09756	+	0.08690	+	0.08722	+
**Morocco Berbers Figuig**	0.06247	+	0.04542	+	0.05800	+
**Egypt Berbers Siwa**	0.10895	+	0.09627	+	0.10557	+
**Libye Tuaregs**	0.12846	+	0.11908	+	0.12261	+
**Ethiopia various**	0.09378	+	0.05562	+	0.09503	+
**Thailand**	0.08227	+	0.07177	+	0.08177	+
**Vietnam**	0.09358	+	0.08819	+	0.09253	+
**Adonara**	0.13116	+	0.12653	+	0.13945	+
**Flores**	0.08710	+	0.10163	+	0.09871	+
**Banjamarsin**	0.02975	+	0.03884	+	0.02490	+
**New Guinea Highlands**	0.25259	+	0.22107	+	0.23589	+
**Solomon islands**	0.10630	+	0.12811	+	0.10710	+
**Bali**	0.06684	+	0.06423	+	0.06663	+
**Sumatra Medan**	0.04542	+	0.04369	+	0.03906	+
**Sumatra Pekanbaru**	0.10916	+	0.10420	+	0.09859	+
**Manus Province**	0.09683	+	0.12830	+	0.10181	+
**Nicobar**	0.20554	+	0.18765	+	0.20302	+
**Onges Nicobar**	0.32064	+	0.28845	+	0.31826	+
**Phillipins**	0.05034	+	0.06103	+	0.04498	+
**Iban**	0.06033	+	0.07499	+	0.06550	+
**Malay Kuala Lumpur**	0.04972	+	0.05190	+	0.04927	+
**Malay Singapore**	0.04378	+	0.04690	+	0.04274	+
**New Guinea Gidra**	0.23700	+	0.19917	+	0.22786	+
**Bismark Archipelago**	0.09704	+	0.10592	+	0.09680	+
**Vanuatu**	0.20821	+	0.18571	+	0.19405	+
**Socotra**	0.17724	+	0.15978	+	0.16561	+
**Iran**	0.10943	+	0.10852	+	0.10039	+
**Syria**	0.12800	+	0.12024	+	0.11536	+
**Yemen**	0.05559	+	0.03587	+	0.04877	+
**Israel**	0.09226	+	0.08427	+	0.08405	+
**Kurds**	0.13986	+	0.13276	+	0.13012	+
**Cyprus**	0.12860	+	0.11876	+	0.11585	+
**Irak**	0.13200	+	0.12602	+	0.11922	+
**Iranian**	0.14142	+	0.14845	+	0.12968	+
**Dubaï**	0.05482	+	0.05209	+	0.05009	+
**Israel Druzes**	0.11304	+	0.11458	+	0.10255	+
**Turkey various**	0.09878	+	0.10274	+	0.08912	+
**Jordan**	0.10029	+	0.09711	+	0.09070	+
**Iran Kurds**	0.11875	+	0.11773	+	0.11330	+
**Iran persian**	0.09001	+	0.09220	+	0.08300	+

**Saudi Arabia**	0.09682	+	0.09747	+	0.08927	+
**Bangladesh**	0.07566	+	0.06392	+	0.06978	+
**Gujarat**	0.07272	+	0.07014	+	0.06688	+
**India Kamataka**	0.09924	+	0.09787	+	0.09870	+
**India Kerala**	0.09643	+	0.09415	+	0.08908	+
**Madyah Pradesh**	0.07874	+	0.07195	+	0.07057	+
**India Maharashtra**	0.07309	+	0.06866	+	0.06764	+
**India Orissa**	0.06453	+	0.06705	+	0.06019	+
**Punjab**	0.08482	+	0.08768	+	0.07957	+
**Tamil Nadu**	0.07087	+	0.07347	+	0.06917	+
**India Tripura**	0.06450	+	0.05639	+	0.06377	+
**Uttard Pradesh**	0.08493	+	0.08333	+	0.07997	+
**Western Bengal**	0.06903	+	0.06565	+	0.06733	+
**Sri Lanka**	0.06777	+	0.06561	+	0.06261	+
**Hindus India**	0.07336	+	0.06996	+	0.06934	+
**Sicily**	0.13612	+	0.13838	+	0.12421	+
**Galicia**	0.18710	+	0.18053	+	0.17246	+
**Catalan**	0.13107	+	0.12519	+	0.12026	+
**Andalusia**	0.14915	+	0.14781	+	0.13732	+
**Portuguese**	0.15341	+	0.14304	+	0.14158	+
**Corsica**	0.13907	+	0.13813	+	0.12944	+
**France**	0.16960	+	0.16472	+	0.15596	+
**England**	0.15254	+	0.14757	+	0.14054	+
**Greece**	0.15678	+	0.15814	+	0.14518	+

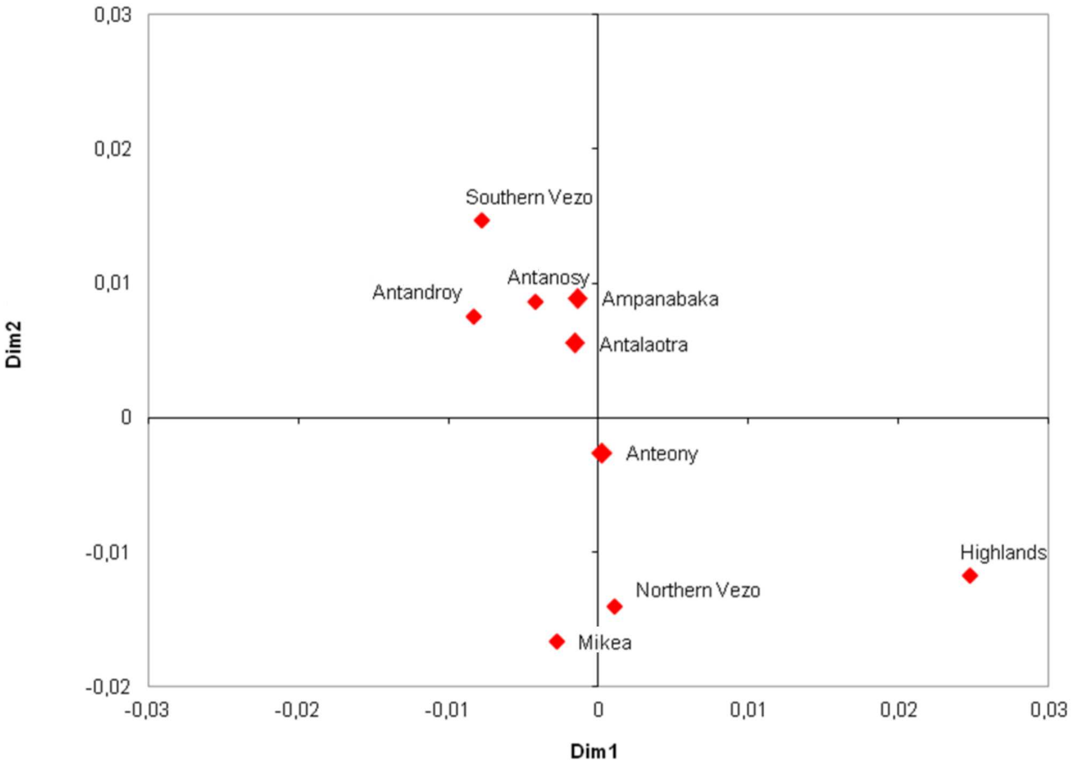

Figure S3. MDS plot computed from F_{ST} values between Malagasy populations, based on HVI data. Lower diagonal: F_{ST}; upper diagonal: p-values. (+) p-value significant at 1% ; (-) p-values non-significant at 1%. Kruskal stress = 0.329.

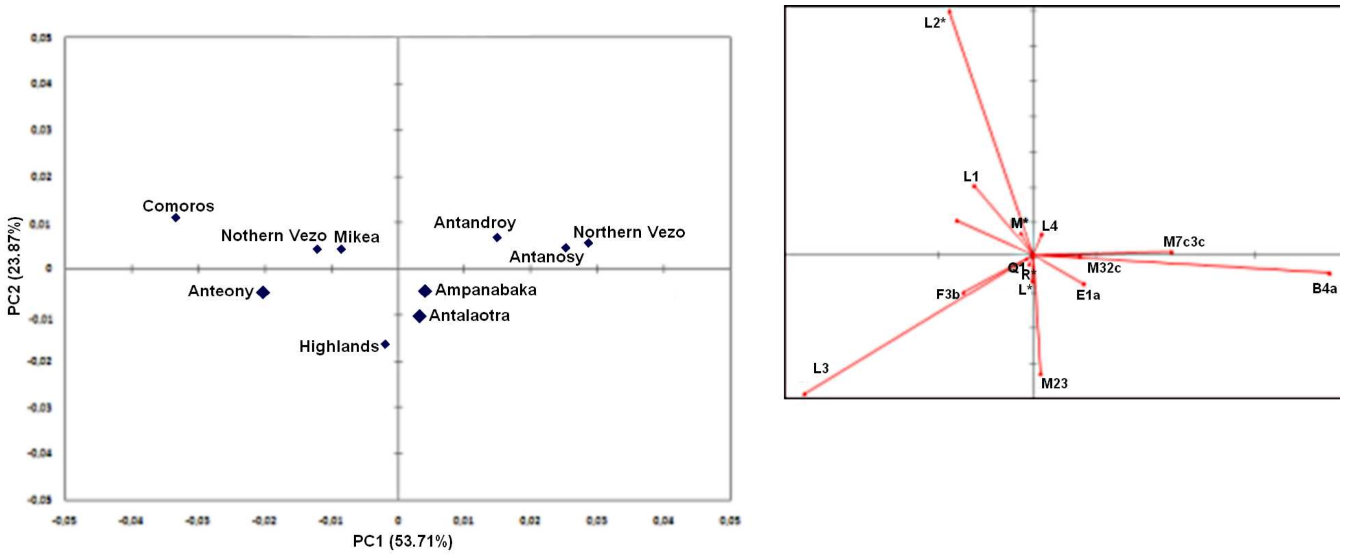

Figure S4. PCA computed from mitochondrial haplogroups frequency in Malagasy and Comoros populations. The insert showed the contribution of haplogroups to the two components.

Table S16. Shared HVI haplotypes between the Ampanabaka and the other Malagasy populations.

	Ampanabaka	Andriana	Antaisaka	Highlands	Antanosy	Antandroy	Mikea	Vezo - Northern	Vezo - Southern
**n ind.**	**48**	**32**	**11**	**52**	**54**	**59**	**127**	**52**	**49**
**n hap.**	**20**	**7**	**8**	**21**	**25**	**23**	**28**	**18**	**20**
Hap.01	1	0	0	0	0	1	1	0	0
Hap.02	1	0	0	0	1	2	0	0	0
Hap.03	4	0	1	2	8	4	6	2	6
Hap.04	4	9	0	3	3	2	18	6	0
Hap.05	1	0	1	0	0	0	0	0	0
Hap.06	4	0	0	1	0	0	0	0	1
Hap.07	2	0	1	0	1	1	0	0	0
Hap.08	1	0	0	0	0	0	0	0	0
Hap.09	3	0	3	0	2	0	0	0	0
Hap.10	11	16	2	4	11	16	17	4	15
Hap.11	1	0	0	0	0	0	0	0	0
Hap.12	2	0	0	8	2	4	10	2	1
Hap.13	2	0	0	1	1	2	14	6	7
Hap.14	1	0	0	0	0	0	1	0	0
Hap.15	1	0	0	0	0	0	0	1	0
Hap.16	2	3	1	0	3	1	12	1	4
Hap.17	2	0	0	2	0	0	1	4	0
Hap.18	3	0	0	0	2	2	5	5	1
Hap.19	1	0	0	0	0	1	0	0	0
Hap.20	1	0	0	0	0	0	0	0	0

Table S17. Shared HVI haplotypes between the Antalaotra and other Malagasy populations

	Antalaotra	Andriana	Antaisaka	Highlands	Antanosy	Antandroy	Mikea	Vezo - Northern	Vezo - Southern
**n ind.**	**40**	**32**	**11**	**52**	**54**	**59**	**127**	**52**	**49**
**n hap.**	**27**	**7**	**8**	**21**	**25**	**23**	**28**	**18**	**20**
Hap.01	1	0	1	2	8	4	6	2	6
Hap.02	5	9	0	3	3	2	18	6	0
Hap.03	2	0	0	1	0	0	0	0	1
Hap.04	2	0	3	0	2	0	0	0	0
Hap.05	8	16	2	4	11	16	17	4	15
Hap.06	5	0	0	8	2	4	10	2	1
Hap.07	2	0	0	1	1	2	14	6	7
Hap.08	1	3	1	0	3	1	12	1	4
Hap.09	3	0	0	0	2	2	5	5	1
Hap.10	1	0	0	0	0	0	0	0	0
Hap.11	2	1	0	1	2	7	3	3	1
Hap.12	2	0	0	0	0	0	0	0	0
Hap.13	1	0	0	0	0	0	0	0	0
Hap.14	1	0	0	0	0	0	0	0	0
Hap.15	1	0	0	0	4	0	0	2	1
Hap.16	2	0	0	0	0	0	0	0	0
Hap.17	1	0	0	0	0	0	0	0	0

Table S18. Shared HVI haplotypes between the Anteony and other Malagasy populations.

	Anteony	Andriana	Antaisaka	Highlands	Antanosy	Antandroy	Mikea	Vezo - Northern	Vezo - Southern
**n ind.**	**47**	**32**	**11**	**52**	**54**	**59**	**127**	**52**	**49**
**n hap.**	**17**	**7**	**8**	**21**	**25**	**23**	**28**	**18**	**20**
Hap.01	1	0	0	0	0	1	1	0	0
Hap.02	1	0	0	0	1	2	0	0	0
Hap.03	1	0	1	2	8	4	6	2	6
Hap.04	3	9	0	3	3	2	18	6	0
Hap.05	1	0	1	0	1	1	0	0	0
Hap.06	1	0	3	0	2	0	0	0	0
Hap.07	2	16	2	4	11	16	17	4	15
Hap.08	3	0	0	8	2	4	10	2	1
Hap.09	4	0	0	1	1	2	14	6	7
Hap.10	1	0	0	2	0	0	1	4	0
Hap.11	6	0	0	0	2	2	5	5	1
Hap.12	1	0	0	0	0	1	0	0	0
Hap.13	1	0	0	0	0	0	0	0	0
Hap.14	3	0	0	0	0	0	0	0	0
Hap.15	1	0	0	0	1	0	5	0	0
Hap.16	3	0	0	0	0	0	0	0	0
Hap.17	3	1	0	2	2	0	0	0	1
Hap.18	1	0	0	0	0	0	0	0	0
Hap.19	1	0	0	0	0	0	0	0	0
Hap.20	1	0	0	0	1	0	0	0	0
Hap.21	2	0	0	0	0	0	0	0	1
Hap.22	1	1	0	1	2	7	3	3	1
Hap.23	1	0	0	0	0	0	0	0	0
Hap.24	1	0	0	0	0	0	0	0	0
Hap.25	1	0	0	0	0	0	0	0	0
Hap.26	1	0	0	0	0	0	0	0	0
Hap.27	1	0	0	0	0	0	2	0	1

Table S19. Shared HVI unique haplotypes between the Ampanabaka and populations from the database

	Number of indiviuals	Number of haplotypes	hap.01	hap.02	hap.03	hap.04	hap.05	hap.06	hap.07	hap.08	hap.09	hap.10	hap.11	hap.12	hap.13	hap.14	hap.15	hap.16	hap.17	hap.18	hap.19	hap.20
			L3d	L2a1	M7c3c	L3b	L3b	L3e3	L3e2b3	M32c	L0a2	B4a1a1a	B4a1a1a	M23	L2a1	L3b	L2a1	E1a1	L3e1a	F3b	L2a1	M7c3c
**Angola Nyaneka**	**147**	**70**	1	0	0	0	0	1	0	0	1	0	0	0	0	0	0	0	0	0	0	0
**Angola Ovimbudu**	**98**	**60**	1	0	0	0	0	1	0	0	1	0	0	0	1	0	0	0	1	0	0	0
**Fon Benin**	**171**	**104**	1	0	0	0	0	0	0	0	0	0	0	0	0	0	1	0	0	0	0	0
**Cabinda Angola**	**110**	**69**	0	0	0	0	0	1	0	0	1	0	0	0	0	0	0	0	1	0	0	0
**Cabo verde**	**292**	**118**	0	0	0	0	0	0	0	0	0	1	0	0	0	0	1	0	0	0	0	0
**Cameroon Ngumba**	**88**	**43**	0	0	0	0	0	0	0	0	0	0	0	0	0	0	1	0	0	0	0	0
**Cameroon Pygmies Baka**	**87**	**13**	0	0	0	0	0	0	0	0	0	0	0	0	0	0	0	0	0	0	0	0
**Cameroon Pygmies Baloka**	**88**	**5**	0	0	0	0	0	0	0	0	0	0	0	0	0	0	0	0	0	0	0	0
**Guanche Canaries Islands**	**71**	**32**	0	0	0	0	0	0	0	0	0	0	0	0	0	0	0	0	0	0	0	0
**Egypt Arabs**	**102**	**44**	0	0	0	0	0	0	0	0	0	0	0	0	0	0	0	0	0	0	0	0
**Egypt Copt**	**100**	**30**	0	0	0	0	0	0	0	0	0	0	0	0	0	0	0	0	0	0	0	0
**Gabon Fong**	**66**	**35**	0	0	0	0	0	0	0	0	0	0	0	0	0	0	0	0	1	0	0	0
**Gabon Mitsogo**	**64**	**33**	0	0	0	0	0	0	0	0	0	0	0	0	0	0	0	0	0	0	0	0
**Gabon Nzebi**	**63**	**42**	1	0	0	0	0	0	0	0	0	0	0	0	0	0	0	0	1	0	0	0
**Guinea Bissau**	**372**	**176**	1	0	0	1	0	0	0	0	0	0	0	0	0	0	1	0	0	0	0	0
**Ivory coast Ahizi**	**129**	**54**	0	0	0	0	0	0	0	0	0	0	0	0	0	0	1	0	0	0	0	0
**Ivory coast Yacouba**	**61**	**41**	0	0	0	0	0	0	0	0	0	0	0	0	0	0	0	0	0	0	0	0
**Kenya swahili**	**200**	**117**	0	0	0	0	0	1	0	0	1	0	0	0	0	0	1	0	0	0	0	0
**Mali Malinke**	**60**	**48**	0	0	0	0	0	0	0	0	0	0	0	0	0	0	0	0	0	0	0	0
**Moors Mauritania**	**64**	**107**	0	0	0	0	0	0	0	0	0	0	0	0	0	0	1	0	0	0	0	0
**Morocco Berbers**	**181**	**107**	0	0	0	0	1	0	0	0	0	0	0	0	0	0	0	0	0	0	0	0
**Mozambique**	**416**	**135**	1	0	0	0	0	1	0	0	1	0	0	0	1	0	1	0	1	0	0	0
**Rwanda Hutu**	**106**	**58**	1	0	0	0	0	0	0	0	0	0	0	0	0	0	1	0	0	0	0	0
**Sao Tome**	**103**	**61**	0	0	0	0	0	0	0	0	0	0	0	0	0	0	0	0	0	0	0	0
**Senegal Mendenka**	**119**	**50**	0	0	0	0	0	0	0	0	0	0	0	0	0	0	0	0	0	0	0	0
**Senegal Wolof**	**91**	**76**	0	0	0	0	0	0	0	0	0	0	0	0	0	0	1	0	0	0	0	0

**South Africa Kung**	**59**	**14**	0	0	0	0	0	0	0	0	0	0	0	0	0	0	0	0	0	0	0	0
**Tanzania Hadza**	**79**	**26**	0	0	0	0	1	0	0	0	0	0	0	0	0	0	1	0	0	0	0	0
**Tanzania Sandawe**	**82**	**28**	0	0	0	0	0	0	0	0	0	0	0	0	0	0	0	0	0	0	0	0
**Zimbabwe**	**58**	**38**	1	0	0	0	0	0	0	0	1	0	0	0	1	0	1	0	0	0	0	0
**Tunisia**	**47**	**42**	0	0	0	0	0	0	0	0	0	0	0	0	0	0	0	0	0	0	0	0
**Algeria**	**47**	**26**	0	0	0	0	0	0	0	0	0	0	0	0	0	0	0	0	0	0	0	0
**Sahawari**	**56**	**41**	0	0	0	0	0	0	0	0	0	0	0	0	0	0	0	0	0	0	0	0
**Samar Tunisia**	**124**	**6**	0	0	0	0	0	0	0	0	0	0	0	0	0	0	0	0	0	0	0	0
**Ethiopia Amharic**	**270**	**139**	1	0	0	0	0	0	0	0	0	0	0	0	0	0	1	0	0	0	0	0
**Sudan-Nubia**	**161**	**106**	0	0	0	0	0	0	0	0	0	0	0	0	0	0	1	0	0	0	0	0
**Tunisia various**	**102**	**45**	0	0	0	0	0	0	0	0	0	0	0	0	0	0	0	0	0	0	0	0
**Morocco Berbers Bourhiah**	**70**	**35**	0	0	0	0	0	0	0	0	0	0	0	0	0	0	0	0	0	0	0	0
**Morocco Berbers Figuig**	**94**	**28**	0	0	0	0	0	0	0	0	0	0	0	0	0	0	0	0	0	0	0	0
**Egypt Berbers Siwa**	**78**	**22**	0	0	0	0	0	0	0	0	0	0	0	0	0	0	0	0	1	0	0	0
**Lybia various**	**129**	**16**	0	0	0	0	0	0	0	0	0	0	0	0	0	0	0	0	0	0	0	0
**Ethiopia various**	**116**	**71**	1	0	0	0	0	0	0	0	1	0	0	0	0	0	0	0	0	0	0	0
**Thailand various**	**71**	**61**	0	0	0	0	0	0	0	0	0	0	0	0	0	0	0	0	0	0	0	0
**Vietnam**	**65**	**50**	0	0	0	0	0	0	0	0	0	0	0	0	0	0	0	0	0	0	0	0
**Adonara**	**73**	**30**	0	0	0	0	0	0	0	0	0	0	0	0	0	0	0	0	0	0	0	0
**Flores**	**73**	**37**	0	0	0	0	0	0	0	0	0	1	0	0	0	0	0	0	0	0	0	0
**Banjamarsin**	**110**	**80**	0	0	1	0	0	0	0	0	0	1	0	0	0	0	0	0	0	0	0	0
**New Guinea Highlands**	**71**	**26**	0	0	0	0	0	0	0	0	0	0	0	0	0	0	0	0	0	0	0	0
**Solomon islands**	**64**	**14**	0	0	0	0	0	0	0	0	0	1	0	0	0	0	0	0	0	0	0	0
**Bali**	**64**	**52**	0	0	1	0	0	0	0	0	0	0	0	0	0	0	0	0	0	0	0	0
**Sumatra Medan**	**42**	**29**	0	0	1	0	0	0	0	0	0	0	0	0	0	0	0	0	0	0	0	0
**Sumatra Pekanbaru**	**54**	**34**	0	0	1	0	0	0	0	0	0	0	0	0	0	0	0	0	0	0	0	0
**Manus Province**	**144**	**35**	0	0	1	0	0	0	0	0	0	1	0	0	0	0	0	0	0	0	0	0
**Nicobar**	**46**	**22**	0	0	0	0	0	0	0	0	0	0	0	0	0	0	0	0	0	0	0	0
**Onges Nicobar**	**63**	**6**	0	0	0	0	0	0	0	0	0	0	0	0	0	0	0	0	0	0	0	0
**Philippins**	**144**	**70**	0	0	1	0	0	0	0	0	0	0	0	0	0	0	0	0	0	1	0	0
**Iban**	**83**	**30**	0	0	0	0	0	0	0	0	0	0	0	0	0	0	0	0	0	0	0	0
**Malay Kuala-Lumpur**	**124**	**90**	0	0	1	0	0	0	0	0	0	0	0	0	0	0	0	0	0	0	0	0
**Malay Singapore**	**205**	**132**	0	0	1	0	0	0	0	0	0	0	0	0	0	0	0	0	0	0	0	0

**New Guinea Gidra**	**59**	**20**	0	0	0	0	0	0	0	0	0	0	0	0	0	0	0	0	0	0	0	0
**Bismark Archipelago**	**47**	**21**	0	0	0	0	0	0	0	0	0	1	0	0	0	0	0	0	0	0	0	0
**Vanuatu**	**42**	**11**	0	0	0	0	0	0	0	0	0	1	0	0	0	0	0	0	0	0	0	0
**Socotra**	**65**	**17**	0	0	0	0	0	0	0	0	0	0	0	0	0	0	0	0	0	0	0	0
**Iran**	**146**	**113**	0	0	0	0	0	0	0	0	0	0	0	0	0	0	0	0	0	0	0	0
**Syria**	**49**	**46**	0	0	0	0	0	0	0	0	0	0	0	0	0	0	0	0	0	0	0	0
**Yemen**	**115**	**67**	1	0	0	0	0	1	0	0	1	0	0	0	0	0	0	0	0	0	0	0
**Israel**	**45**	**25**	0	0	0	0	0	0	0	0	0	0	0	0	0	0	0	0	0	0	0	0
**Kurds**	**78**	**57**	0	0	0	0	0	0	0	0	0	0	0	0	0	0	0	0	0	0	0	0
**Cyprus**	**91**	**59**	0	0	0	0	0	0	0	0	0	0	0	0	0	0	0	0	0	0	0	0
**Irak**	**52**	**52**	0	0	0	0	0	0	0	0	0	0	0	0	0	0	0	0	0	0	0	0
**Iranians**	**731**	**394**	0	0	0	0	0	0	0	0	0	0	0	0	0	0	0	0	0	0	0	0
**Dubai**	**249**	**154**	1	0	0	0	0	1	0	0	1	0	0	1	1	0	0	0	0	0	0	0
**Israel Druzes**	**311**	**79**	0	0	0	0	0	0	0	0	0	0	0	0	0	0	0	0	0	0	0	0
**Turkey various**	**234**	**184**	0	0	0	0	0	0	0	0	0	0	0	0	0	0	0	0	0	0	0	0
**Jordan**	**99**	**80**	0	0	0	0	0	0	0	0	0	0	0	0	0	0	1	0	0	0	0	0
**Iran Kurds**	**25**	**22**	0	0	0	0	0	0	0	0	0	0	0	0	0	0	0	0	0	0	0	0
**Persians**	**82**	**61**	0	0	0	0	0	0	0	0	0	0	0	0	0	0	0	0	0	0	0	0
**Saudi Arabia**	**553**	**260**	1	0	0	1	0	0	0	0	1	0	0	0	0	0	1	0	0	0	0	0
**Bangladesh**	**30**	**27**	0	0	0	0	0	0	0	0	0	0	0	0	0	0	0	0	0	0	0	0
**Gujarat**	**91**	**79**	0	0	0	0	0	0	0	0	0	0	0	0	0	0	0	0	0	0	0	0
**India Kamataka**	**201**	**60**	0	0	0	0	0	0	0	0	0	0	0	0	0	0	0	0	0	0	0	0
**India Kerala**	**230**	**72**	0	0	0	0	0	0	0	0	0	0	0	0	0	0	0	0	0	0	0	0
**Madyah Pradesh**	**82**	**54**	0	0	0	0	0	0	0	0	0	0	0	0	0	0	0	0	0	0	0	0
**India Maharashtra**	**221**	**146**	0	0	0	0	0	0	0	0	0	0	0	0	0	0	0	0	0	0	0	0
**India Orissa**	**153**	**106**	0	0	0	0	0	0	0	0	0	0	0	0	0	0	0	0	0	0	0	0
**Punjab**	**362**	**175**	0	0	0	0	0	0	0	0	0	0	0	0	0	0	0	0	0	0	0	0
**Tamils Nadu**	**427**	**207**	0	0	0	0	0	0	0	0	0	0	0	0	0	0	0	0	0	0	0	0
**India Tripura**	**134**	**134**	0	0	0	0	0	0	0	0	0	0	0	0	0	0	0	0	0	0	0	0
**Uttard Pradesh**	**232**	**165**	0	0	0	0	0	0	0	0	0	0	0	0	0	0	0	0	0	0	0	0
**Western Bengal**	**285**	**133**	0	0	0	0	0	0	0	0	0	0	0	0	0	0	0	0	0	0	0	0
**Sri Lanka**	**131**	**91**	0	0	0	0	0	0	0	0	0	0	0	0	0	0	0	0	0	0	0	0
**Hindus India**	**72**	**59**	0	0	0	0	0	0	0	0	0	0	0	0	0	0	0	0	0	0	0	0

**Sicily**	**226**	**133**	0	0	0	0	0	0	0	0	0	0	0	0	0	0	0	0	0	0	0	0
**Galicia**	**92**	**51**	0	0	0	0	0	0	0	0	0	0	0	0	0	0	0	0	0	0	0	0
**Catalan**	**46**	**28**	0	0	0	0	0	0	0	0	0	0	0	0	0	0	0	0	0	0	0	0
**Andalusia**	**115**	**70**	0	0	0	0	0	0	0	0	0	0	0	0	0	0	0	0	0	0	0	0
**Portuguese**	**54**	**37**	0	0	0	0	0	0	0	0	0	0	0	0	0	0	0	0	0	0	0	0
**Corsica**	**53**	**35**	0	0	0	0	0	0	0	0	0	0	0	0	0	0	0	0	0	0	0	0
**France**	**110**	**72**	0	0	0	0	0	0	0	0	0	0	0	0	0	0	0	0	0	0	0	0
**England**	**100**	**66**	0	0	0	0	0	0	0	0	0	0	0	0	0	0	0	0	0	0	0	0
**Greece**	**114**	**55**	0	0	0	0	0	0	0	0	0	0	0	0	0	0	0	0	0	0	0	0

Table S20. Shared HVI unique haplotypes between the Antalaotra and populations from the database

	Number of indiviuals	Number of haplotypes	hap.01 M7c3c	hap.02 L3b	hap.03 L3e3	hap.04 L0a2	hap.05 B4a1a1a	hap.06 M23	hap.07 L2a1	hap.08 E1a1	hap.09 F3b	hap.10 L3e3	hap.11 M32c	hap.12 L3e2b3	hap.13 M7c3c	hap.14 M32c	hap.15 B4a1a1a	hap.16 F3b	hap.17 L3b
**Angola Nyaneka**	147	70	0	0	1	1	0	0	0	0	0	0	0	0	0	0	0	0	0
**Angola Ovimbudu**	98	60	0	0	1	1	0	0	1	0	0	0	0	0	0	0	0	0	0
**Fon Benin**	171	104	0	0	0	0	0	0	0	0	0	0	0	0	0	0	0	0	0
**Cabinda Angola**	110	69	0	0	1	1	0	0	0	0	0	0	0	0	0	0	0	0	0
**Cabo verde**	292	118	0	0	0	0	1	0	0	0	0	0	0	0	0	0	0	0	0
**Cameroon Ngumba**	88	43	0	0	0	0	0	0	0	0	0	0	0	0	0	0	0	0	0
**Cameroon Pygmies Baka**	87	13	0	0	0	0	0	0	0	0	0	0	0	0	0	0	0	0	0
**Cameroon Pygmies Baloka**	88	5	0	0	0	0	0	0	0	0	0	0	0	0	0	0	0	0	0
**Guanche Canaries Islands**	71	32	0	0	0	0	0	0	0	0	0	0	0	0	0	0	0	0	0
**Egypt Arabs**	102	44	0	0	0	0	0	0	0	0	0	0	0	0	0	0	0	0	0
**Egypt Copt**	100	30	0	0	0	0	0	0	0	0	0	0	0	0	0	0	0	0	0
**Gabon Fong**	66	35	0	0	0	0	0	0	0	0	0	0	0	0	0	0	0	0	0
**Gabon Mitsogo**	64	33	0	0	0	0	0	0	0	0	0	0	0	0	0	0	0	0	0
**Gabon Nzebi**	63	42	0	0	0	0	0	0	0	0	0	0	0	0	0	0	0	0	0
**Guinea Bissau**	372	176	0	1	0	0	0	0	0	0	0	0	0	0	0	0	0	0	0
**Ivory coast Ahizi**	129	54	0	0	0	0	0	0	0	0	0	0	0	0	0	0	0	0	0
**Ivory coast Yacouba**	61	41	0	0	0	0	0	0	0	0	0	0	0	0	0	0	0	0	0
**Kenya swahili**	200	117	0	0	1	1	0	0	0	0	0	0	0	0	0	0	0	0	0
**Mali Malinke**	60	48	0	0	0	0	0	0	0	0	0	0	0	0	0	0	0	0	0
**Moors Mauritania**	64	107	0	0	0	0	0	0	0	0	0	0	0	0	0	0	0	0	0
**Morocco Berbers**	181	107	0	0	0	0	0	0	0	0	0	0	0	0	0	0	0	0	0
**Mozambique**	416	135	0	0	1	1	0	0	1	0	0	0	0	0	0	0	0	0	0
**Rwanda Hutu**	106	58	0	0	0	0	0	0	0	0	0	0	0	0	0	0	0	0	0
**Sao Tome**	103	61	0	0	0	0	0	0	0	0	0	0	0	0	0	0	0	0	0
**Senegal Mendenka**	119	50	0	0	0	0	0	0	0	0	0	0	0	0	0	0	0	0	0

**Senegal Wolof**	91	76	0	0	0	0	0	0	0	0	0	0	0	0	0	0	0	0	0
**South Africa Kung**	59	14	0	0	0	0	0	0	0	0	0	0	0	0	0	0	0	0	0
**Tanzania Hadza**	79	26	0	0	0	0	0	0	0	0	0	0	0	0	0	0	0	0	0
**Tanzania Sandawe**	82	28	0	0	0	0	0	0	0	0	0	0	0	0	0	0	0	0	0
**Zimbabwe**	58	38	0	0	0	1	0	0	1	0	0	0	0	0	0	0	0	0	0
**Tunisia**	47	42	0	0	0	0	0	0	0	0	0	0	0	0	0	0	0	0	0
**Algeria**	47	26	0	0	0	0	0	0	0	0	0	0	0	0	0	0	0	0	0
**Sahawari**	56	41	0	0	0	0	0	0	0	0	0	0	0	0	0	0	0	0	0
**Samar Tunisia**	124	6	0	0	0	0	0	0	0	0	0	0	0	0	0	0	0	0	0
**Ethiopia Amharic**	270	139	0	0	0	0	0	0	0	0	0	0	0	0	0	0	0	0	0
**Sudan-Nubia**	161	106	0	0	0	0	0	0	0	0	0	0	0	0	0	0	0	0	0
**Tunisia various**	102	45	0	0	0	0	0	0	0	0	0	0	0	0	0	0	0	0	0
**Morocco Berbers Bourhiah**	70	35	0	0	0	0	0	0	0	0	0	0	0	0	0	0	0	0	0
**Morocco Berbers Figuig**	94	28	0	0	0	0	0	0	0	0	0	0	0	0	0	0	0	0	0
**Egypt Berbers Siwa**	78	22	0	0	0	0	0	0	0	0	0	0	0	0	0	0	0	0	0
**Lybia various**	129	16	0	0	0	0	0	0	0	0	0	0	0	0	0	0	0	0	0
**Ethiopia various**	116	71	0	0	0	1	0	0	0	0	0	0	0	0	0	0	0	0	0
**Thailand various**	71	61	0	0	0	0	0	0	0	0	0	0	0	0	0	0	0	0	0
**Vietnam**	65	50	0	0	0	0	0	0	0	0	0	0	0	0	0	0	0	0	0
**Adonara**	73	30	0	0	0	0	0	0	0	0	0	0	0	0	0	0	1	0	0
**Flores**	73	37	0	0	0	0	1	0	0	0	0	0	0	0	0	0	1	0	0
**Banjamarsin**	110	80	1	0	0	0	1	0	0	0	0	0	1	0	0	0	1	0	0
**New Guinea Highlands**	71	26	0	0	0	0	0	0	0	0	0	0	0	0	0	0	0	0	0
**Solomon islands**	64	14	0	0	0	0	1	0	0	0	0	0	0	0	0	0	1	0	0
**Bali**	64	52	1	0	0	0	0	0	0	0	0	0	0	0	0	0	0	0	0
**Sumatra Medan**	42	29	1	0	0	0	0	0	0	0	0	0	0	0	0	0	0	0	0
**Sumatra Pekanbaru**	54	34	1	0	0	0	0	0	0	0	0	0	0	0	0	0	0	0	0
**Manus Province**	144	35	1	0	0	0	1	0	0	0	0	0	0	0	0	0	1	0	0
**Nicobar**	46	22	0	0	0	0	0	0	0	0	0	0	0	0	0	0	0	0	0
**Onges Nicobar**	63	6	0	0	0	0	0	0	0	0	0	0	0	0	0	0	0	0	0

**Philippins**	144	70	1	0	0	0	0	0	0	0	1	0	0	0	0	0	1	0	0
**Iban**	83	30	0	0	0	0	0	0	0	0	0	0	0	0	0	0	1	0	0
**Malay Kuala-Lumpur**	124	90	1	0	0	0	0	0	0	0	0	0	1	0	1	0	0	0	0
**Malay Singapore**	205	132	1	0	0	0	0	0	0	0	0	0	0	0	1	0	0	0	0
**New Guinea Gidra**	59	20	0	0	0	0	0	0	0	0	0	0	0	0	0	0	0	0	0
**Bismark Archipelago**	47	21	0	0	0	0	1	0	0	0	0	0	0	0	0	0	1	0	0
**Vanuatu**	42	11	0	0	0	0	1	0	0	0	0	0	0	0	0	0	0	0	0
**Socotra**	65	17	0	0	0	0	0	0	0	0	0	0	0	0	0	0	0	0	0
**Iran**	146	113	0	0	0	0	0	0	0	0	0	0	0	0	0	0	0	0	0
**Syria**	49	46	0	0	0	0	0	0	0	0	0	0	0	0	0	0	0	0	0
**Yemen**	115	67	0	0	1	1	0	0	0	0	0	0	0	0	0	0	0	0	0
**Israel**	45	25	0	0	0	0	0	0	0	0	0	0	0	0	0	0	0	0	0
**Kurds**	78	57	0	0	0	0	0	0	0	0	0	0	0	0	0	0	0	0	0
**Cyprus**	91	59	0	0	0	0	0	0	0	0	0	0	0	0	0	0	0	0	0
**Irak**	52	52	0	0	0	0	0	0	0	0	0	0	0	0	0	0	0	0	0
**Iranians**	731	394	0	0	0	0	0	0	0	0	0	0	0	0	0	0	0	0	0
**Dubai**	249	154	0	0	1	1	0	1	1	0	0	0	0	0	0	0	0	0	0
**Israel Druzes**	311	79	0	0	0	0	0	0	0	0	0	0	0	0	0	0	0	0	0
**Turkey various**	234	184	0	0	0	0	0	0	0	0	0	0	0	0	0	0	0	0	0
**Jordan**	99	80	0	0	0	0	0	0	0	0	0	0	0	0	0	0	0	0	0
**Iran Kurds**	25	22	0	0	0	0	0	0	0	0	0	0	0	0	0	0	0	0	0
**Persians**	82	61	0	0	0	0	0	0	0	0	0	0	0	0	0	0	0	0	0
**Saudi Arabia**	553	260	0	1	0	1	0	0	0	0	0	0	0	0	0	0	0	0	0
**Bangladesh**	30	27	0	0	0	0	0	0	0	0	0	0	0	0	0	0	0	0	0
**Gujarat**	91	79	0	0	0	0	0	0	0	0	0	0	0	0	0	0	0	0	0
**India Kamataka**	201	60	0	0	0	0	0	0	0	0	0	0	0	0	0	0	0	0	0
**India Kerala**	230	72	0	0	0	0	0	0	0	0	0	0	0	0	0	0	0	0	0
**Madyah Pradesh**	82	54	0	0	0	0	0	0	0	0	0	0	0	0	0	0	0	0	0
**India Maharashtra**	221	146	0	0	0	0	0	0	0	0	0	0	0	0	0	0	0	0	0
**India Orissa**	153	106	0	0	0	0	0	0	0	0	0	0	0	0	0	0	0	0	0
**Punjab**	362	175	0	0	0	0	0	0	0	0	0	0	0	0	0	0	0	0	0

**Tamils Nadu**	427	207	0	0	0	0	0	0	0	0	0	0	0	0	0	0	0	0	0
**India Tripura**	134	134	0	0	0	0	0	0	0	0	0	0	0	0	0	0	0	0	0
**Uttard Pradesh**	232	165	0	0	0	0	0	0	0	0	0	0	0	0	0	0	0	0	0
**Western Bengal**	285	133	0	0	0	0	0	0	0	0	0	0	0	0	0	0	0	0	0
**Sri Lanka**	131	91	0	0	0	0	0	0	0	0	0	0	0	0	0	0	0	0	0
**Hindus India**	72	59	0	0	0	0	0	0	0	0	0	0	0	0	0	0	0	0	0
**Sicily**	226	133	0	0	0	0	0	0	0	0	0	0	0	0	0	0	0	0	0
**Galicia**	92	51	0	0	0	0	0	0	0	0	0	0	0	0	0	0	0	0	0
**Catalan**	46	28	0	0	0	0	0	0	0	0	0	0	0	0	0	0	0	0	0
**Andalusia**	115	70	0	0	0	0	0	0	0	0	0	0	0	0	0	0	0	0	0
**Portuguese**	54	37	0	0	0	0	0	0	0	0	0	0	0	0	0	0	0	0	0
**Corsica**	53	35	0	0	0	0	0	0	0	0	0	0	0	0	0	0	0	0	0
**France**	110	72	0	0	0	0	0	0	0	0	0	0	0	0	0	0	0	0	0
**England**	100	66	0	0	0	0	0	0	0	0	0	0	0	0	0	0	0	0	0
**Greece**	114	55	0	0	0	0	0	0	0	0	0	0	0	0	0	0	0	0	0

Table S21. Shared HVI unique haplotypes between the Anteony and populations from the database

	Number of indiviuals	Number of haplotypes	hap.01	hap.02	hap.03	hap.04	hap.05	hap.06	hap.07	hap.08	hap.09	hap.10	hap.11	hap.12	hap.13	hap.14	hap.15	hap.16	hap.17	hap.18	hap.19	hap.20	hap.21	hap.22	hap.23	hap.24	hap.25	hap.26	hap.27
			**L3d**	**L2a1**	**M7c3c**	**L3b**	**L3e2b3**	**L0a2**	**B4a1a1a**	**M23**	**L2a1**	**L3e1a**	**F3b**	**L2a1**	**L3b**	**L0a1'4**	**L3a**	**B4a1a1a**	**L3b**	**L3b**	**L3e3**	**L3e2b**	**M23**	**M32c**	**L2a1**	**L3e3**	**L3e2b3**	**O1**	**L3k**
**Angola Nyaneka**	147	70	1	0	0	0	0	1	0	0	0	0	0	0	0	1	0	0	0	0	0	1	0	0	0	0	0	0	0
**Angola Ovimbudu**	98	60	1	0	0	0	0	1	0	0	1	1	0	0	0	1	0	0	0	0	0	1	0	0	0	0	0	0	0
**Fon Benin**	171	104	1	0	0	0	0	0	0	0	0	0	0	0	0	0	0	0	1	0	0	1	0	0	0	0	0	0	0
**Cabinda Angola**	110	69	0	0	0	0	0	1	0	0	0	1	0	0	0	1	0	0	0	0	0	1	0	0	0	0	0	0	0
**Cabo verde**	292	118	0	0	0	0	0	0	1	0	0	0	0	0	0	0	0	0	0	0	0	1	0	0	0	0	0	0	0
**Cameroon Ngumba**	88	43	0	0	0	0	0	0	0	0	0	0	0	0	0	0	0	0	0	0	0	1	0	0	0	0	0	0	0
**Cameroon Pygmies Baka**	87	13	0	0	0	0	0	0	0	0	0	0	0	0	0	0	0	0	0	0	0	0	0	0	0	0	0	0	0
**Cameroon Pygmies Baloka**	88	5	0	0	0	0	0	0	0	0	0	0	0	0	0	0	0	0	0	0	0	0	0	0	0	0	0	0	0
**Guanche Canaries Islands**	71	32	0	0	0	0	0	0	0	0	0	0	0	0	0	0	0	0	0	0	0	0	0	0	0	0	0	0	0
**Egypt Arabs**	102	44	0	0	0	0	0	0	0	0	0	0	0	0	0	0	0	0	0	0	0	0	0	0	0	0	0	0	0
**Egypt Copt**	100	30	0	0	0	0	0	0	0	0	0	0	0	0	0	0	0	0	0	0	0	0	0	0	0	0	0	0	0
**Gabon Fong**	66	35	0	0	0	0	0	0	0	0	0	1	0	0	0	0	0	0	0	0	0	1	0	0	0	0	0	0	0
**Gabon Mitsogo**	64	33	0	0	0	0	0	0	0	0	0	0	0	0	0	0	0	0	0	0	0	0	0	0	0	0	0	0	0
**Gabon Nzebi**	63	42	1	0	0	0	0	0	0	0	0	1	0	0	0	0	0	0	0	0	0	1	0	0	0	0	0	0	0
**Guinea Bissau**	372	176	1	0	0	1	0	0	0	0	0	0	0	0	0	0	0	0	1	0	0	1	0	0	0	0	0	0	0
**Ivory coast Ahizi**	129	54	0	0	0	0	0	0	0	0	0	0	0	0	0	0	0	0	0	0	0	1	0	0	0	0	0	0	0
**Ivory coast Yacouba**	61	41	0	0	0	0	0	0	0	0	0	0	0	0	0	0	0	0	0	0	0	0	0	0	0	0	0	0	0
**Kenya swahili**	200	117	0	0	0	0	0	1	0	0	0	0	0	0	0	1	0	0	0	0	0	1	0	0	0	0	0	0	0
**Mali Malinke**	60	48	0	0	0	0	0	0	0	0	0	0	0	0	0	0	0	0	1	0	0	0	0	0	0	0	0	0	0
**Moors Mauritania**	64	107	0	0	0	0	0	0	0	0	0	0	0	0	0	0	0	0	0	0	0	0	0	0	0	0	0	0	0
**Morocco Berbers**	181	107	0	0	0	0	0	0	0	0	0	0	0	0	0	1	0	0	0	0	0	0	0	0	0	0	0	0	0
**Mozambique**	416	135	1	0	0	0	0	1	0	0	1	1	0	0	0	1	0	0	0	0	0	1	0	0	0	0	0	0	0

**Rwanda Hutu**	106	58	1	0	0	0	0	0	0	0	0	0	0	0	0	1	0	0	0	0	0	1	0	0	0	0	0	0	0
**Sao Tome**	103	61	0	0	0	0	0	0	0	0	0	0	0	0	0	0	0	0	1	0	0	0	0	0	0	0	0	0	0
**Senegal Mendenka**	119	50	0	0	0	0	0	0	0	0	0	0	0	0	0	0	0	0	1	0	0	0	0	0	0	0	0	0	0
**Senegal Wolof**	91	76	0	0	0	0	0	0	0	0	0	0	0	0	0	0	0	0	0	0	0	1	0	0	0	0	0	0	0
**South Africa Kung**	59	14	0	0	0	0	0	0	0	0	0	0	0	0	0	0	0	0	0	0	0	1	0	0	0	0	0	0	0
**Tanzania Hadza**	79	26	0	0	0	0	0	0	0	0	0	0	0	0	0	0	0	0	0	0	0	0	0	0	0	0	0	0	0
**Tanzania Sandawe**	82	28	0	0	0	0	0	0	0	0	0	0	0	0	0	0	0	0	0	0	0	0	0	0	0	0	0	0	0
**Zimbabwe**	58	38	1	0	0	0	0	1	0	0	1	0	0	0	0	1	0	0	0	0	0	1	0	0	0	0	0	0	0
**Tunisia**	47	42	0	0	0	0	0	0	0	0	0	0	0	0	0	0	0	0	0	0	0	0	0	0	0	0	0	0	0
**Algeria**	47	26	0	0	0	0	0	0	0	0	0	0	0	0	0	0	0	0	0	0	0	0	0	0	0	0	0	0	0
**Sahawari**	56	41	0	0	0	0	0	0	0	0	0	0	0	0	0	0	0	0	0	0	0	0	0	0	0	0	0	0	0
**Samar Tunisia**	124	6	0	0	0	0	0	0	0	0	0	0	0	0	0	0	0	0	0	0	0	0	0	0	0	0	0	0	0
**Ethiopia Amharic**	270	139	1	0	0	0	0	0	0	0	0	0	0	0	0	0	0	0	0	0	0	0	0	0	0	0	0	0	0
**Sudan-Nubia**	161	106	0	0	0	0	0	0	0	0	0	0	0	0	0	0	0	0	0	0	0	0	0	0	0	0	0	0	0
**Tunisia various**	102	45	0	0	0	0	0	0	0	0	0	0	0	0	0	0	0	0	0	0	0	0	0	0	0	0	0	0	0
**Morocco Berbers Bourhiah**	70	35	0	0	0	0	0	0	0	0	0	0	0	0	0	0	0	0	0	0	0	0	0	0	0	0	0	0	0
**Morocco Berbers Figuig**	94	28	0	0	0	0	0	0	0	0	0	0	0	0	0	0	0	0	0	0	0	1	0	0	0	0	0	0	0
**Egypt Berbers Siwa**	78	22	0	0	0	0	0	0	0	0	0	1	0	0	0	0	0	0	0	0	0	0	0	0	0	0	0	0	0
**Lybia various**	129	16	0	0	0	0	0	0	0	0	0	0	0	0	0	0	0	0	0	0	0	1	0	0	0	0	0	0	0
**Ethiopia various**	116	71	1	0	0	0	0	1	0	0	0	0	0	0	0	1	0	0	0	0	0	0	0	0	0	0	0	0	0
**Thailand various**	65	50	0	0	0	0	0	0	0	0	0	0	0	0	0	0	0	0	0	0	0	0	0	0	0	0	0	0	0
**Vietnam**	73	30	0	0	0	0	0	0	0	0	0	0	0	0	0	0	0	0	0	0	0	0	0	0	0	0	0	0	0
**Adonara**	73	37	0	0	0	0	0	0	1	0	0	0	0	0	0	0	0	0	0	0	0	0	0	0	0	0	0	0	0
**Flores**	110	80	0	0	1	0	0	0	1	0	0	0	0	0	0	0	0	0	0	0	0	0	0	1	0	0	0	0	0
**Banjamarsin**	71	26	0	0	0	0	0	0	0	0	0	0	0	0	0	0	0	0	0	0	0	0	0	0	0	0	0	0	0
**New Guinea Highlands**	64	14	0	0	0	0	0	0	1	0	0	0	0	0	0	0	0	0	0	0	0	0	0	0	0	0	0	0	0
**Solomon islands**	64	52	0	0	1	0	0	0	0	0	0	0	0	0	0	0	0	0	0	0	0	0	0	0	0	0	0	0	0
**Bali**	42	29	0	0	1	0	0	0	0	0	0	0	0	0	0	0	0	0	0	0	0	0	0	0	0	0	0	0	0
**Sumatra Medan**	54	34	0	0	1	0	0	0	0	0	0	0	0	0	0	0	0	0	0	0	0	0	0	0	0	0	0	0	0

**Sumatra Pekanbaru**	144	35	0	0	1	0	0	0	1	0	0	0	0	0	0	0	0	0	0	0	0	0	0	0	0	0	0	0	0
**Manus Province**	46	22	0	0	0	0	0	0	0	0	0	0	0	0	0	0	0	0	0	0	0	0	0	0	0	0	0	0	0
**Nicobar**	63	6	0	0	0	0	0	0	0	0	0	0	0	0	0	0	0	0	0	0	0	0	0	0	0	0	0	0	0
**Onges Nicobar**	144	70	0	0	1	0	0	0	0	0	0	0	1	0	0	0	0	0	0	0	0	0	0	0	0	0	0	0	0
**Philippins**	98	23	0	0	0	0	0	0	0	0	0	0	0	0	0	0	0	0	0	0	0	0	0	0	0	0	0	0	0
**Iban**	83	30	0	0	0	0	0	0	0	0	0	0	0	0	0	0	0	0	0	0	0	0	0	0	0	0	0	0	0
**Malay Kuala-Lumpur**	124	90	0	0	1	0	0	0	0	0	0	0	0	0	0	0	0	0	0	0	0	0	0	1	0	0	0	0	0
**Malay Singapore**	205	132	0	0	1	0	0	0	0	0	0	0	0	0	0	0	0	0	0	0	0	0	0	0	0	0	0	0	0
**New Guinea Gidra**	59	20	0	0	0	0	0	0	0	0	0	0	0	0	0	0	0	0	0	0	0	0	0	0	0	0	0	0	0
**Bismark Archipelago**	47	21	0	0	0	0	0	0	1	0	0	0	0	0	0	0	0	0	0	0	0	0	0	0	0	0	0	0	0
**Vanuatu**	42	11	0	0	0	0	0	0	1	0	0	0	0	0	0	0	0	0	0	0	0	0	0	0	0	0	0	0	0
**Socotra**	65	17	0	0	0	0	0	0	0	0	0	0	0	0	0	0	0	0	0	0	0	0	0	0	0	0	0	0	0
**Iran**	146	113	0	0	0	0	0	0	0	0	0	0	0	0	0	0	0	0	0	0	0	0	0	0	0	0	0	0	0
**Syria**	49	46	0	0	0	0	0	0	0	0	0	0	0	0	0	0	0	0	0	0	0	0	0	0	0	0	0	0	0
**Yemen**	115	67	1	0	0	0	0	1	0	0	0	0	0	0	0	0	0	0	0	0	0	0	0	0	0	0	0	0	0
**Israel**	45	25	0	0	0	0	0	0	0	0	0	0	0	0	0	0	0	0	0	0	0	0	0	0	0	0	0	0	0
**Kurds**	78	57	0	0	0	0	0	0	0	0	0	0	0	0	0	0	0	0	0	0	0	0	0	0	0	0	0	0	0
**Cyprus**	91	59	0	0	0	0	0	0	0	0	0	0	0	0	0	0	0	0	0	0	0	0	0	0	0	0	0	0	0
**Irak**	52	52	0	0	0	0	0	0	0	0	0	0	0	0	0	0	0	0	0	0	0	0	0	0	0	0	0	0	0
**Iranians**	731	394	0	0	0	0	0	0	0	0	0	0	0	0	0	0	0	0	1	0	0	0	0	0	0	0	0	0	0
**Dubai**	249	154	1	0	0	0	0	1	0	1	1	0	0	0	0	1	0	0	0	0	0	0	0	0	0	0	0	0	0
**Israel Druzes**	311	79	0	0	0	0	0	0	0	0	0	0	0	0	0	0	0	0	0	0	0	0	0	0	0	0	0	0	0
**Turkey various**	234	184	0	0	0	0	0	0	0	0	0	0	0	0	0	0	0	0	1	0	0	0	0	0	0	0	0	0	0
**Jordan**	99	80	0	0	0	0	0	0	0	0	0	0	0	0	0	0	0	0	0	0	0	0	0	0	0	0	0	0	0
**Iran Kurds**	25	22	0	0	0	0	0	0	0	0	0	0	0	0	0	0	0	0	0	0	0	0	0	0	0	0	0	0	0
**Persians**	82	61	0	0	0	0	0	0	0	0	0	0	0	0	0	0	0	0	0	0	0	0	0	0	0	0	0	0	0
**Saudi Arabia**	553	260	1	0	0	1	0	1	0	0	0	0	0	0	0	0	0	0	0	0	0	0	0	0	0	0	0	1	0
**Bangladesh**	30	27	0	0	0	0	0	0	0	0	0	0	0	0	0	0	0	0	0	0	0	0	0	0	0	0	0	0	0
**Gujarat**	91	79	0	0	0	0	0	0	0	0	0	0	0	0	0	0	0	0	0	0	0	0	0	0	0	0	0	0	0

**India Kamataka**	201	60	0	0	0	0	0	0	0	0	0	0	0	0	0	0	0	0	0	0	0	0	0	0	0	0	0	0	0
**India Kerala**	230	72	0	0	0	0	0	0	0	0	0	0	0	0	0	0	0	0	0	0	0	0	0	0	0	0	0	0	0
**Madyah Pradesh**	82	54	0	0	0	0	0	0	0	0	0	0	0	0	0	0	0	0	0	0	0	0	0	0	0	0	0	0	0
**India Maharashtra**	221	146	0	0	0	0	0	0	0	0	0	0	0	0	0	0	0	0	0	0	0	0	0	0	0	0	0	0	0
**India Orissa**	153	106	0	0	0	0	0	0	0	0	0	0	0	0	0	0	0	0	0	0	0	0	0	0	0	0	0	0	0
**Punjab**	362	175	0	0	0	0	0	0	0	0	0	0	0	0	0	0	0	0	0	0	0	0	0	0	0	0	0	0	0
**Tamils Nadu**	427	207	0	0	0	0	0	0	0	0	0	0	0	0	0	0	0	0	0	0	0	0	0	0	0	0	0	0	0
**India Tripura**	134	134	0	0	0	0	0	0	0	0	0	0	0	0	0	0	0	0	0	0	0	0	0	0	0	0	0	0	0
**Uttard Pradesh**	232	165	0	0	0	0	0	0	0	0	0	0	0	0	0	0	0	0	0	0	0	0	0	0	0	0	0	0	0
**Western Bengal**	285	133	0	0	0	0	0	0	0	0	0	0	0	0	0	0	0	0	0	0	0	0	0	0	0	0	0	0	0
**Sri Lanka**	131	91	0	0	0	0	0	0	0	0	0	0	0	0	0	0	0	0	0	0	0	0	0	0	0	0	0	0	0
**Hindus India**	72	59	0	0	0	0	0	0	0	0	0	0	0	0	0	0	0	0	0	0	0	0	0	0	0	0	0	0	0
**Sicily**	226	133	0	0	0	0	0	0	0	0	0	0	0	0	0	0	0	0	0	0	0	0	0	0	0	0	0	0	0
**Galicia**	92	51	0	0	0	0	0	0	0	0	0	0	0	0	0	0	0	0	0	0	0	0	0	0	0	0	0	0	0
**Catalan**	46	28	0	0	0	0	0	0	0	0	0	0	0	0	0	0	0	0	0	0	0	0	0	0	0	0	0	0	0
**Andalusia**	115	70	0	0	0	0	0	0	0	0	0	0	0	0	0	0	0	0	0	0	0	0	0	0	0	0	0	0	0
**Portuguese**	54	37	0	0	0	0	0	0	0	0	0	0	0	0	0	0	0	0	0	0	0	0	0	0	0	0	0	0	0
**Corsica**	53	35	0	0	0	0	0	0	0	0	0	0	0	0	0	0	0	0	0	0	0	0	0	0	0	0	0	0	0
**France**	110	72	0	0	0	0	0	0	0	0	0	0	0	0	0	0	0	0	0	0	0	0	0	0	0	0	0	0	0
**England**	100	66	0	0	0	0	0	0	0	0	0	0	0	0	0	0	0	0	0	0	0	0	0	0	0	0	0	0	0
**Greece**	114	55	0	0	0	0	0	0	0	0	0	0	0	0	0	0	0	0	0	0	0	0	0	0	0	0	0	0	0

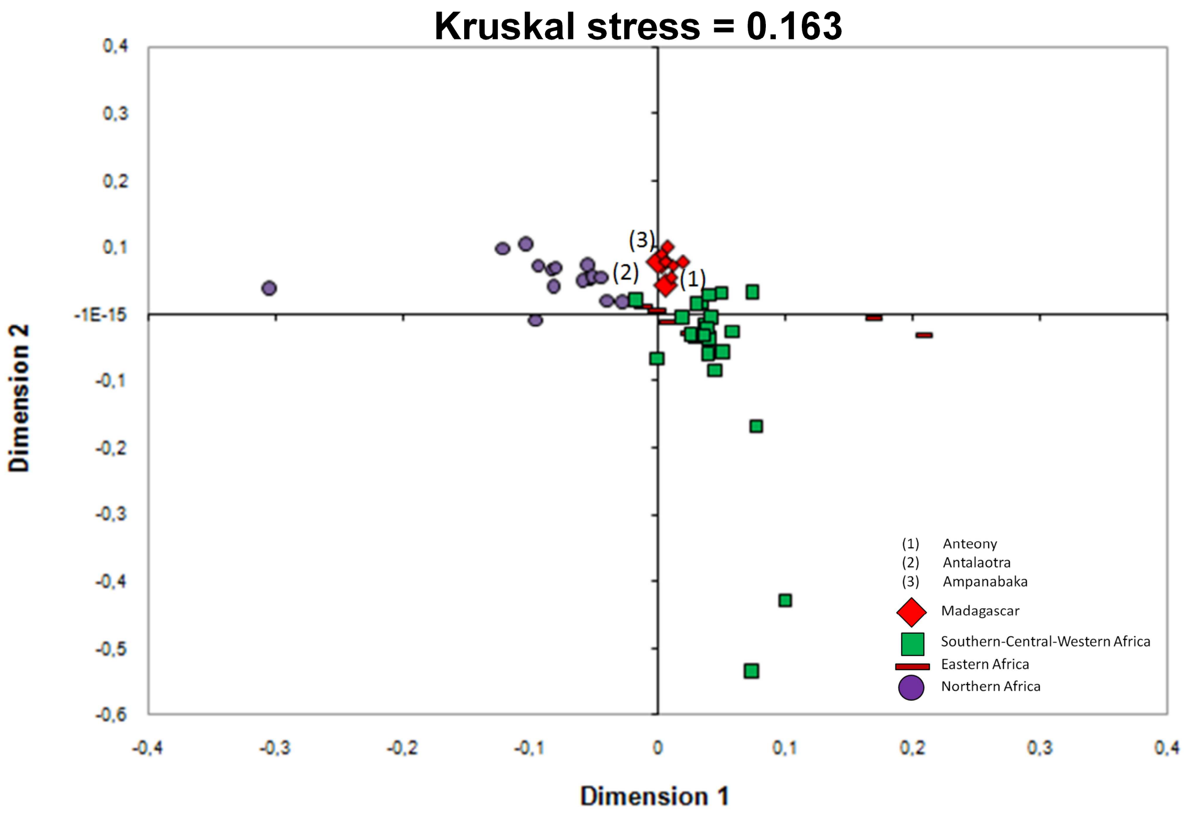
Kruskal stress = 0.163
Dimension 1
Dimension 2
(1)
(2)
(3)
(1) Anteony
(2) Antalaotra
(3) Ampanabaka
Madagascar
Southern-Central-Western Africa
Eastern Africa
Northern Africa

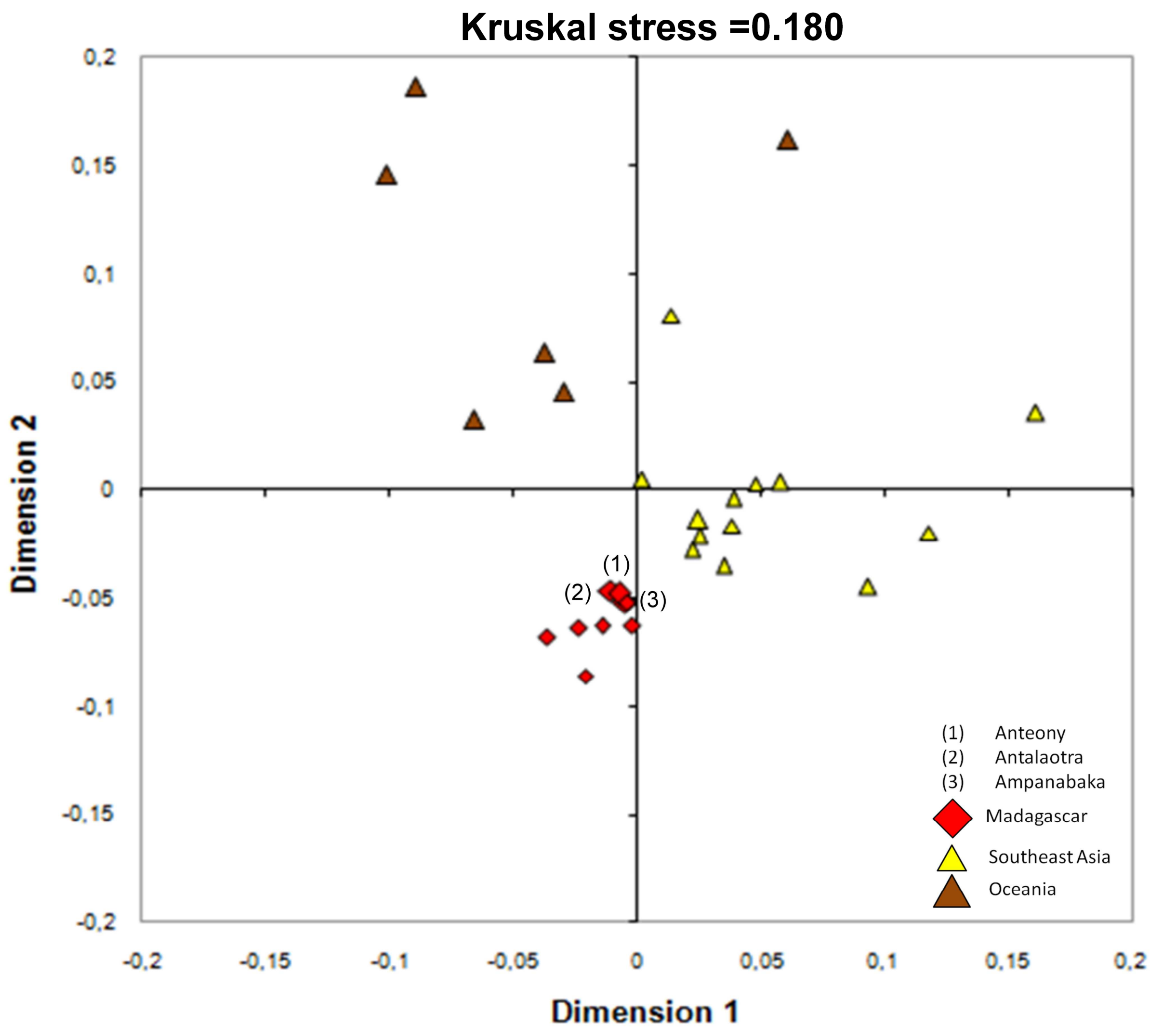
Kruskal stress =0.180
Dimension 1
Dimension 2
(1)
(2)
(3)
(1) Anteony
(2) Antalaotra
(3) Ampanabaka
Madagascar
Southeast Asia
Oceania

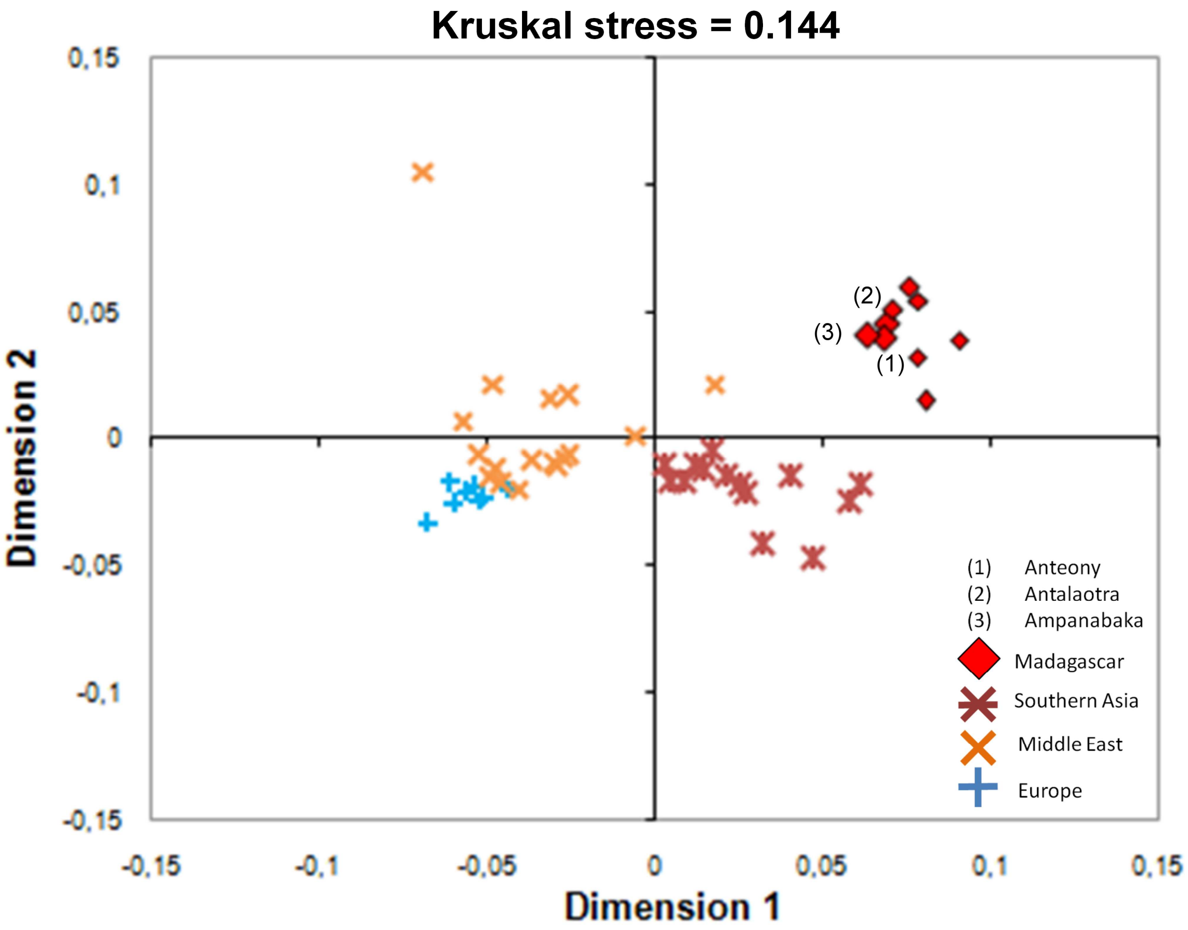

Figure S5. MDS plots of FST computed from HVI haplotypes between the Antemoro and populations from various geographic regions. (a) African populations, (b) Southeast Asian and Oceanian populations; (c) European and west Eurasian populations.

References

1. Beleza S, Gusmao L, Amorim A, Carracedo A, Salas A (2005) The genetic legacy of western Bantu migrations. Hum Genet 117: 366-375.
2. Coelho M, Sequeira F, Luiselli D, Beleza S, Rocha J (2009) On the edge of Bantu expansions: mtDNA, Y chromosome and lactase persistence genetic variation in southwestern Angola. BMC Evol Biol 9: 80.
3. Cruciani F, Santolamazza P, Shen P, Macaulay V, Moral P, et al. (2002) A back migration from Asia to sub-Saharan Africa is supported by high-resolution analysis of human Y-chromosome haplotypes. Am J Hum Genet 70: 1197-1214.
4. Luis JR, Rowold DJ, Regueiro M, Caeiro B, Cinnioglu C, et al. (2004) The Levant versus the Horn of Africa: evidence for bidirectional corridors of human migrations. Am J Hum Genet 74: 532-544.
5. de Filippo C, Barbieri C, Whitten M, Mpoloka SW, Gunnarsdottir ED, et al. (2011) Y-chromosomal variation in sub-Saharan Africa: insights into the history of Niger-Congo groups. Mol Biol Evol 28: 1255-1269.
6. Arroyo-Pardo E, Gusmao L, Lopez-Parra AM, Baeza C, Mesa MS, et al. (2005) Genetic variability of 16 Y-chromosome STRs in a sample from Equatorial Guinea (Central Africa). Forensic Sci Int 149: 109-113.
7. Brucato N, Cassar O, Tonasso L, Tortevoye P, Migot-Nabias F, et al. (2010) The imprint of the Slave Trade in an African American population: mitochondrial DNA, Y chromosome and HTLV-1 analysis in the Noir Marron of French Guiana. BMC Evol Biol 10: 314.
8. Goncalves R, Freitas A, Branco M, Rosa A, Fernandes AT, et al. (2005) Y-chromosome lineages from Portugal, Madeira and Acores record elements of Sephardim and Berber ancestry. Ann Hum Genet 69: 443-454.
9. Semino O, Santachiara-Benerecetti AS, Falaschi F, Cavalli-Sforza LL, Underhill PA (2002) Ethiopians and Khoisan share the deepest clades of the human Y-chromosome phylogeny. Am J Hum Genet 70: 265-268.
10. Knight A, Underhill PA, Mortensen HM, Zhivotovsky LA, Lin AA, et al. (2003) African Y chromosome and mtDNA divergence provides insight into the history of click languages. Curr Biol 13: 464-473.
11. Hallenberg C, Simonsen B, Sanchez J, Morling N (2005) Y-chromosome STR haplotypes in Somalis. Forensic Sci Int 151: 317-321.
12. Wood ET, Stover DA, Ehret C, Destro-Bisol G, Spedini G, et al. (2005) Contrasting patterns of Y chromosome and mtDNA variation in Africa: evidence for sex-biased demographic processes. Eur J Hum Genet 13: 867-876.
13. Gomes V, Alves C, Amorim A, Carracedo A, Sanchez-Diz P, et al. (2009) Nilotes from Karamoja, Uganda: haplotype data defined by 17 Y-chromosome STRs. Forensic Sci Int Genet 4: e83-86.
14. Abu-Amero KK, Hellani A, Gonzalez AM, Larruga JM, Cabrera VM, et al. (2009) Saudi Arabian Y-Chromosome diversity and its relationship with nearby regions. BMC Genet 10: 59.
15. Robino C, Crobu F, Di Gaetano C, Bekada A, Benhamamouch S, et al. (2008) Analysis of Y-chromosomal SNP haplogroups and STR haplotypes in an Algerian population sample. Int J Legal Med 122: 251-255.
16. Immel A-D, Erhuma M, Mustafa T, Kleiber M, Klintshar M (2006) Y-chromosomal STR haplotypes in an Arab population from Lybia. International Congress Series 1288: 156-158.
17. Zalloua PA, Platt DE, El Sibai M, Khalife J, Makhoul N, et al. (2008) Identifying genetic traces of historical expansions: Phoenician footprints in the Mediterranean. Am J Hum Genet 83: 633-642.
18. Arredi B, Poloni ES, Paracchini S, Zerjal T, Fathallah DM, et al. (2004) A predominantly neolithic origin for Y-chromosomal DNA variation in North Africa. Am J Hum Genet 75: 338-345.

19. Thanseem I, Thangaraj K, Chaubey G, Singh VK, Bhaskar LV, et al. (2006) Genetic affinities among the lower castes and tribal groups of India: inference from Y chromosome and mitochondrial DNA. BMC Genet 7: 42.
20. Thangaraj K, Naidu BP, Crivellaro F, Tamang R, Upadhyay S, et al. (2010) The influence of natural barriers in shaping the genetic structure of Maharashtra populations. PLoS One 5: e15283.
21. Eaaswarkhanth M, Haque I, Ravesh Z, Romero IG, Meganathan PR, et al. (2009) Traces of sub-Saharan and Middle Eastern lineages in Indian Muslim populations. Eur J Hum Genet 18: 354-363.
22. Sengupta S, Zhivotovsky LA, King R, Mehdi SQ, Edmonds CA, et al. (2006) Polarity and temporality of high-resolution y-chromosome distributions in India identify both indigenous and exogenous expansions and reveal minor genetic influence of Central Asian pastoralists. Am J Hum Genet 78: 202-221.
23. Adams SM, Bosch E, Balaresque PL, Ballereau SJ, Lee AC, et al. (2008) The genetic legacy of religious diversity and intolerance: paternal lineages of Christians, Jews, and Muslims in the Iberian Peninsula. Am J Hum Genet 83: 725-736.
24. Nogueiro I, Manco L, Gomes V, Amorim A, Gusmao L (2010) Phylogeographic analysis of paternal lineages in NE Portuguese Jewish communities. Am J Phys Anthropol 141: 373-381.
25. Tofanelli S, Bertoncini S, Castri L, Luiselli D, Calafell F, et al. (2009) On the origins and admixture of Malagasy: new evidence from high-resolution analyses of paternal and maternal lineages. Mol Biol Evol 26: 2109-2124.
26. Msaidie S, Ducourneau A, Boetsch G, Longepied G, Papa K, et al. (2011) Genetic diversity on the Comoros Islands shows early seafaring as major determinant of human biocultural evolution in the Western Indian Ocean. Eur J Hum Genet 19: 89-94.
27. Razafindrazaka H (2010) Le peuplement humain de Madagascar :
Anthropologie génétique de trois groupes traditionnels. Toulouse: Université Toulouse III - Paul Sabatier. 399 p.
28. Hurles ME, Sykes BC, Jobling MA, Forster P (2005) The dual origin of the Malagasy in Island Southeast Asia and East Africa: evidence from maternal and paternal lineages. Am J Hum Genet 76: 894-901.
29. Shlush LI, Behar DM, Yudkovsky G, Templeton A, Hadid Y, et al. (2008) The Druze: a population genetic refugium of the Near East. PLoS One 3: e2105.
30. Mohammad T, Xue Y, Evison M, Tyler-Smith C (2009) Genetic structure of nomadic Bedouin from Kuwait. Heredity 103: 425-433.
31. Cerny V, Pereira L, Kujanova M, Vasikova A, Hajek M, et al. (2009) Out of Arabia-the settlement of island Soqotra as revealed by mitochondrial and Y chromosome genetic diversity. Am J Phys Anthropol 138: 439-447.
32. Cadenas AM, Zhivotovsky LA, Cavalli-Sforza LL, Underhill PA, Herrera RJ (2008) Y-chromosome diversity characterizes the Gulf of Oman. Eur J Hum Genet 16: 374-386.
33. Zalloua PA, Xue Y, Khalife J, Makhoul N, Debiane L, et al. (2008) Y-chromosomal diversity in Lebanon is structured by recent historical events. Am J Hum Genet 82: 873-882.
34. Flores C, Maca-Meyer N, Larruga JM, Cabrera VM, Karadsheh N, et al. (2005) Isolates in a corridor of migrations: a high-resolution analysis of Y-chromosome variation in Jordan. J Hum Genet 50: 435-441.
35. Sanchez JJ, Hallenberg C, Borsting C, Hernandez A, Morling N (2005) High frequencies of Y chromosome lineages characterized by E3b1, DYS19-11, DYS392-12 in Somali males. Eur J Hum Genet 13: 856-866.
36. Al-Zahery N, Semino O, Benuzzi G, Magri C, Passarino G, et al. (2003) Y-chromosome and mtDNA polymorphisms in Iraq, a crossroad of the early human dispersal and of post-Neolithic migrations. Mol Phylogenet Evol 28: 458-472.

37. Regueiro M, Cadenas AM, Gayden T, Underhill PA, Herrera RJ (2006) Iran: tricontinental nexus for Y-chromosome driven migration. Hum Hered 61: 132-143.
38. El-Sibai M, Platt DE, Haber M, Xue Y, Youhanna SC, et al. (2009) Geographical structure of the Y-chromosomal genetic landscape of the Levant: a coastal-inland contrast. Ann Hum Genet 73: 568-581.
39. Haber M, Platt DE, Badro DA, Xue Y, El-Sibai M, et al. (2011) Influences of history, geography, and religion on genetic structure: the Maronites in Lebanon. Eur J Hum Genet 19: 334-340.
40. Cinnioglu C, King R, Kivisild T, Kalfoglu E, Atasoy S, et al. (2004) Excavating Y-chromosome haplotype strata in Anatolia. Hum Genet 114: 127-148.
41. Karafet TM, Hallmark B, Cox MP, Sudoyo H, Downey S, et al. (2010) Major east-west division underlies Y chromosome stratification across Indonesia. Mol Biol Evol 27: 1833-1844.
42. Kayser M, Brauer S, Cordaux R, Casto A, Lao O, et al. (2006) Melanesian and Asian origins of Polynesians: mtDNA and Y chromosome gradients across the Pacific. Mol Biol Evol 23: 2234-2244.
43. van Oven M, Hammerle JM, van Schoor M, Kushnick G, Pennekamp P, et al. (2010) Unexpected island effects at an extreme: reduced Y chromosome and mitochondrial DNA diversity in Nias. Mol Biol Evol 28: 1349-1361.
44. Mona S, Grunz KE, Brauer S, Pakendorf B, Castri L, et al. (2009) Genetic admixture history of Eastern Indonesia as revealed by Y-chromosome and mitochondrial DNA analysis. Mol Biol Evol 26: 1865-1877.
45. Delfin F, Salvador JM, Calacal GC, Perdigon HB, Tabbada KA, et al. (2010) The Y-chromosome landscape of the Philippines: extensive heterogeneity and varying genetic affinities of Negrito and non-Negrito groups. Eur J Hum Genet 19: 224-230.
46. Berniell-Lee G, Calafell F, Bosch E, Heyer E, Sica L, et al. (2009) Genetic and demographic implications of the Bantu expansion: insights from human paternal lineages. Mol Biol Evol 26: 1581-1589.
47. Lecerf M, Filali M, Gresenguet G, Ndjoyi-Mbiguino A, Le Goff J, et al. (2007) Allele frequencies and haplotypes of eight Y-short tandem repeats in Bantu population living in Central Africa. Forensic Sci Int 171: 212-215.
48. Rosa A, Ornelas C, Brehm A, Villems R (2006) Population data on 11 Y-chromosome STRs from Guine-Bissau. Forensic Sci Int 157: 210-217.
49. Alves C, Gusmao L, Barbosa J, Amorim A (2003) Evaluating the informative power of Y-STRs: a comparative study using European and new African haplotype data. Forensic Sci Int 134: 126-133.
50. Fujihara J, Yuasa I, Muro T, Iida R, Tsubota E, et al. (2009) Allele frequencies and haplotypes for 28 Y-STRs in Ovambo population. Leg Med (Tokyo) 11: 205-208.
51. Tishkoff SA, Gonder MK, Henn BM, Mortensen H, Knight A, et al. (2007) History of click-speaking populations of Africa inferred from mtDNA and Y chromosome genetic variation. Mol Biol Evol 24: 2180-2195.
52. Quintana-Murci L, Bigham A, Rouba H, Barakat A, McElreavey K, et al. (2004) Y-chromosomal STR haplotypes in Berber and Arabic-speaking populations from Morocco. Forensic Sci Int 140: 113-115.
53. Bosch E, Calafell F, Perez-Lezaun A, Comas D, Izaabel H, et al. (2000) Y chromosome STR haplotypes in four populations from northwest Africa. Int J Legal Med 114: 36-40.
54. Ayadi I, Ammar-Keskes L, Rebai A (2006) Haplotypes for 13 Y-chromosomal STR loci in South Tunisian population (Sfax region). Forensic Sci Int 164: 249-253.
55. Nasidze I, Schadlich H, Stoneking M (2003) Haplotypes from the Caucasus, Turkey and Iran for nine Y-STR loci. Forensic Sci Int 137: 85-93.
56. Alshamali F, Pereira L, Budowle B, Poloni ES, Currat M (2009) Local population structure in Arabian Peninsula revealed by Y-STR diversity. Hum Hered 68: 45-54.

57. Alakoc YD, Gokcumen O, Tug A, Gultekin T, Gulec E, et al. (2010) Y-chromosome and autosomal STR diversity in four proximate settlements in Central Anatolia. Forensic Sci Int Genet 4: e135-137.
58. Dobashi Y, Kido A, Fujitani N, Susukida R, Hara M, et al. (2005) Y-chromosome STR haplotypes in a Bangladeshi population. Leg Med (Tokyo) 7: 122-126.
59. Yadav B, Raina A, Dogra TD (2010) Genetic polymorphisms for 17 Y-chromosomal STR haplotypes in Jammu and Kashmir Saraswat Brahmin population. Leg Med (Tokyo) 12: 249-255.
60. Balamurugan K, Suhasini G, Vijaya M, Kanthimathi S, Mullins N, et al. (2010) Y chromosome STR allelic and haplotype diversity in five ethnic Tamil populations from Tamil Nadu, India. Leg Med (Tokyo) 12: 265-269.
61. Nagy M, Henke L, Henke J, Chatthopadhyay PK, Volgyi A, et al. (2007) Searching for the origin of Romanies: Slovakian Romani, Jats of Haryana and Jat Sikhs Y-STR data in comparison with different Romani populations. Forensic Sci Int 169: 19-26.
62. Illeperuma RJ, Markalanda D, Mountain JL, Ratnasooriya WD, Fernandopulle ND, et al. (2009) Haplotype data for 12 Y-chromosome STR loci of Sri Lankans. Forensic Sci Int Genet 4: e119-120.
63. Kwak KD, Jin HJ, Shin DJ, Kim JM, Roewer L, et al. (2005) Y-chromosomal STR haplotypes and their applications to forensic and population studies in east Asia. Int J Legal Med 119: 195-201.
64. Yong RY, Lee LK, Yap EP (2006) Y-chromosome STR haplotype diversity in three ethnic populations in Singapore. Forensic Sci Int 159: 244-257.
65. Chang YM, Perumal R, Keat PY, Kuehn DL (2007) Haplotype diversity of 16 Y-chromosomal STRs in three main ethnic populations (Malays, Chinese and Indians) in Malaysia. Forensic Sci Int 167: 70-76.
66. Chang YM, Swaran Y, Phoon YK, Sothirasan K, Sim HT, et al. (2009) Haplotype diversity of 17 Y-chromosomal STRs in three native Sarawak populations (Iban, Bidayuh and Melanau) in East Malaysia. Forensic Sci Int Genet 3: e77-80.
67. Souto L, Gusmao L, Ferreira E, Amorim A, Corte-Real F, et al. (2006) Y-chromosome STR haplotypes in East Timor: forensic evaluation and population data. Forensic Sci Int 156: 261-265.
68. Rowold DJ, Luis JR, Terreros MC, Herrera RJ (2007) Mitochondrial DNA geneflow indicates preferred usage of the Levant Corridor over the Horn of Africa passageway. J Hum Genet 52: 436-447.
69. Brehm A, Pereira L, Bandelt HJ, Prata MJ, Amorim A (2002) Mitochondrial portrait of the Cabo Verde archipelago: the Senegambian outpost of Atlantic slave trade. Ann Hum Genet 66: 49-60.
70. Quintana-Murci L, Quach H, Harmant C, Luca F, Massonnet B, et al. (2008) Maternal traces of deep common ancestry and asymmetric gene flow between Pygmy hunter-gatherers and Bantu-speaking farmers. Proc Natl Acad Sci U S A 105: 1596-1601.
71. Rosa A, Brehm A, Kivisild T, Metspalu E, Villems R (2004) MtDNA profile of West Africa Guineans: towards a better understanding of the Senegambia region. Ann Hum Genet 68: 340-352.
72. Ely B, Wilson JL, Jackson F, Jackson BA (2006) African-American mitochondrial DNAs often match mtDNAs found in multiple African ethnic groups. BMC Biol 4: 34.
73. Pereira L, Macaulay V, Torroni A, Scozzari R, Prata MJ, et al. (2001) Prehistoric and historic traces in the mtDNA of Mozambique: insights into the Bantu expansions and the slave trade. Ann Hum Genet 65: 439-458.
74. Salas A, Richards M, De la Fe T, Lareu MV, Sobrino B, et al. (2002) The making of the African mtDNA landscape. Am J Hum Genet 71: 1082-1111.
75. Castri L, Tofanelli S, Garagnani P, Bini C, Fosella X, et al. (2009) mtDNA variability in two Bantu-speaking populations (Shona and Hutu) from Eastern Africa: implications for peopling and migration patterns in sub-Saharan Africa. Am J Phys Anthropol 140: 302-311.

76. Trovoada MJ, Pereira L, Gusmao L, Abade A, Amorim A, et al. (2004) Pattern of mtDNA variation in three populations from Sao Tome e Principe. Ann Hum Genet 68: 40-54.
77. Graven L, Passarino G, Semino O, Boursot P, Santachiara-Benerecetti S, et al. (1995) Evolutionary correlation between control region sequence and restriction polymorphisms in the mitochondrial genome of a large Senegalese Mandenka sample. Mol Biol Evol 12: 334-345.
78. Rando JC, Pinto F, Gonzalez AM, Hernandez M, Larruga JM, et al. (1998) Mitochondrial DNA analysis of northwest African populations reveals genetic exchanges with European, near-eastern, and sub-Saharan populations. Ann Hum Genet 62: 531-550.
79. Chen YS, Olckers A, Schurr TG, Kogelnik AM, Huoponen K, et al. (2000) mtDNA variation in the South African Kung and Khwe-and their genetic relationships to other African populations. Am J Hum Genet 66: 1362-1383.
80. Maca-Meyer N, Arnay M, Rando JC, Flores C, Gonzalez AM, et al. (2004) Ancient mtDNA analysis and the origin of the Guanches. Eur J Hum Genet 12: 155-162.
81. Goncalves VF, Prosdocimi F, Santos LS, Ortega JM, Pena SDJ (2007) Sex-biased gene flow in African Americans but not in Americans Caucasians. Genetics and Molecular Research 6: 256-261.
82. Brakez Z, Bosch E, Izaabel H, Akhayat O, Comas D, et al. (2001) Human mitochondrial DNA sequence variation in the Moroccan population of the Souss area. Ann Hum Biol 28: 295-307.
83. Plaza S, Calafell F, Helal A, Bouzerna N, Lefranc G, et al. (2003) Joining the pillars of Hercules: mtDNA sequences show multidirectional gene flow in the western Mediterranean. Ann Hum Genet 67: 312-328.
84. Corte-Real HB, Macaulay VA, Richards MB, Hariti G, Issad MS, et al. (1996) Genetic diversity in the Iberian Peninsula determined from mitochondrial sequence analysis. Ann Hum Genet 60: 331-350.
85. Ottoni C, Martinez-Labarga C, Loogvali EL, Pennarun E, Achilli A, et al. (2009) First genetic insight into Libyan Tuaregs: a maternal perspective. Ann Hum Genet 73: 438-448.
86. Turchi C, Buscemi L, Giacchino E, Onofri V, Fendt L, et al. (2009) Polymorphisms of mtDNA control region in Tunisian and Moroccan populations: an enrichment of forensic mtDNA databases with Northern Africa data. Forensic Sci Int Genet 3: 166-172.
87. Coudray C, Olivieri A, Achilli A, Pala M, Melhaoui M, et al. (2009) The complex and diversified mitochondrial gene pool of Berber populations. Ann Hum Genet 73: 196-214.
88. Brandstatter A, Peterson CT, Irwin JA, Mpoke S, Koech DK, et al. (2004) Mitochondrial DNA control region sequences from Nairobi (Kenya): inferring phylogenetic parameters for the establishment of a forensic database. Int J Legal Med 118: 294-306.
89. Kivisild T, Reidla M, Metspalu E, Rosa A, Brehm A, et al. (2004) Ethiopian mitochondrial DNA heritage: tracking gene flow across and around the gate of tears. Am J Hum Genet 75: 752-770.
90. Krings M, Salem AE, Bauer K, Geisert H, Malek AK, et al. (1999) mtDNA analysis of Nile River Valley populations: A genetic corridor or a barrier to migration? Am J Hum Genet 64: 1166-1176.
91. Poloni ES, Naciri Y, Bucho R, Niba R, Kervaire B, et al. (2009) Genetic evidence for complexity in ethnic differentiation and history in East Africa. Ann Hum Genet 73: 582-600.
92. Nasidze I, Quinque D, Ozturk M, Bendukidze N, Stoneking M (2005) MtDNA and Y-chromosome variation in Kurdish groups. Ann Hum Genet 69: 401-412.
93. Comas D, Plaza S, Wells RS, Yuldaseva N, Lao O, et al. (2004) Admixture, migrations, and dispersals in Central Asia: evidence from maternal DNA lineages. Eur J Hum Genet 12: 495-504.
94. Vernesi C, Di Benedetto G, Caramelli D, Secchieri E, Simoni L, et al. (2001) Genetic characterization of the body attributed to the evangelist Luke. Proc Natl Acad Sci U S A 98: 13460-13463.

95. Macaulay V, Richards M, Hickey E, Vega E, Cruciani F, et al. (1999) The emerging tree of West Eurasian mtDNAs: a synthesis of control-region sequences and RFLPs. Am J Hum Genet 64: 232-249.
96. Comas D, Calafell F, Bendukidze N, Fananas L, Bertranpetit J (2000) Georgian and kurd mtDNA sequence analysis shows a lack of correlation between languages and female genetic lineages. Am J Phys Anthropol 112: 5-16.
97. Irwin J, Saunier J, Strouss K, Paintner C, Diegoli T, et al. (2008) Mitochondrial control region sequences from northern Greece and Greek Cypriots. Int J Legal Med 122: 87-89.
98. Metspalu M, Kivisild T, Metspalu E, Parik J, Hudjashov G, et al. (2004) Most of the extant mtDNA boundaries in south and southwest Asia were likely shaped during the initial settlement of Eurasia by anatomically modern humans. BMC Genet 5: 26.
99. Alshamali F, Brandstatter A, Zimmermann B, Parson W (2008) Mitochondrial DNA control region variation in Dubai, United Arab Emirates. Forensic Sci Int Genet 2: e9-10.
100. Quintana-Murci L, Chaix R, Wells RS, Behar DM, Sayar H, et al. (2004) Where west meets east: the complex mtDNA landscape of the southwest and Central Asian corridor. Am J Hum Genet 74: 827-845.
101. Calafell F, Underhill P, Tolun A, Angelicheva D, Kalaydjieva L (1996) From Asia to Europe: mitochondrial DNA sequence variability in Bulgarians and Turks. Ann Hum Genet 60: 35-49.
102. Gonzalez AM, Karadsheh N, Maca-Meyer N, Flores C, Cabrera VM, et al. (2008) Mitochondrial DNA variation in Jordanians and their genetic relationship to other Middle East populations. Ann Hum Biol 35: 212-231.
103. Derenko M, Malyarchuk B, Grzybowski T, Denisova G, Dambueva I, et al. (2007) Phylogeographic analysis of mitochondrial DNA in northern Asian populations. Am J Hum Genet 81: 1025-1041.
104. Abu-Amero KK, Gonzalez AM, Larruga JM, Bosley TM, Cabrera VM (2007) Eurasian and African mitochondrial DNA influences in the Saudi Arabian population. BMC Evol Biol 7: 32.
105. Bamshad MJ, Watkins WS, Dixon ME, Jorde LB, Rao BB, et al. (1998) Female gene flow stratifies Hindu castes. Nature 395: 651-652.
106. Mountain JL, Hebert JM, Bhattacharyya S, Underhill PA, Ottolenghi C, et al. (1995) Demographic history of India and mtDNA-sequence diversity. Am J Hum Genet 56: 979-992.
107. Cordaux R, Saha N, Bentley GR, Aunger R, Sirajuddin SM, et al. (2003) Mitochondrial DNA analysis reveals diverse histories of tribal populations from India. Eur J Hum Genet 11: 253-264.
108. Basu A, Mukherjee N, Roy S, Sengupta S, Banerjee S, et al. (2003) Ethnic India: a genomic view, with special reference to peopling and structure. Genome Res 13: 2277-2290.
109. Roychoudhury S, Roy S, Basu A, Banerjee R, Vishwanathan H, et al. (2001) Genomic structures and population histories of linguistically distinct tribal groups of India. Hum Genet 109: 339-350.
110. Baig MM, Khan AA, Kulkarni KM (2004) Mitochondrial DNA diversity in tribal and caste groups of Maharashtra (India) and its implication on their genetic origins. Ann Hum Genet 68: 453-460.
111. Sahoo S, Kashyap VK (2006) Phylogeography of mitochondrial DNA and Y-chromosome haplogroups reveal asymmetric gene flow in populations of Eastern India. Am J Phys Anthropol 131: 84-97.
112. Kivisild T, Bamshad MJ, Kaldma K, Metspalu M, Metspalu E, et al. (1999) Deep common ancestry of indian and western-Eurasian mitochondrial DNA lineages. Curr Biol 9: 1331-1334.
113. Kaur I, Roy S, Chakrabarti S, Sarhadi VK, Majumder PP, et al. (2002) Genomic diversities and affinities among four endogamous groups of Punjab (India) based on autosomal and mitochondrial DNA polymorphisms. Hum Biol 74: 819-836.

114. Sharma S, Saha A, Rai E, Bhat A, Bamezai R (2005) Human mtDNA hypervariable regions, HVR I and II, hint at deep common maternal founder and subsequent maternal gene flow in Indian population groups. J Hum Genet 50: 497-506.
115. Gan RJ, Pan SL, Mustavich LF, Qin ZD, Cai XY, et al. (2008) Pinghua population as an exception of Han Chinese's coherent genetic structure. J Hum Genet 53: 303-313.
116. Fornarino S, Pala M, Battaglia V, Maranta R, Achilli A, et al. (2009) Mitochondrial and Y-chromosome diversity of the Tharus (Nepal): a reservoir of genetic variation. BMC Evol Biol 9: 154.
117. Forster P, Cali F, Rohl A, Metspalu E, D'Anna R, et al. (2002) Continental and subcontinental distributions of mtDNA control region types. Int J Legal Med 116: 99-108.
118. Salas A, Comas D, Lareu MV, Bertranpetit J, Carracedo A (1998) mtDNA analysis of the Galician population: a genetic edge of European variation. Eur J Hum Genet 6: 365-375.
119. Falchi A, Giovannoni L, Calo CM, Piras IS, Moral P, et al. (2006) Genetic history of some western Mediterranean human isolates through mtDNA HVR1 polymorphisms. J Hum Genet 51: 9-14.
120. Sampietro ML, Caramelli D, Lao O, Calafell F, Comas D, et al. (2005) The genetics of the pre-Roman Iberian Peninsula: a mtDNA study of ancient Iberians. Ann Hum Genet 69: 535-548.
121. Varesi L, Memmi M, Cristofari MC, Mameli GE, Calo CM, et al. (2000) Mitochondrial control-region sequence variation in the Corsican population, France. Am J Hum Biol 12: 339-351.
122. Dubut V, Chollet L, Murail P, Cartault F, Beraud-Colomb E, et al. (2004) mtDNA polymorphisms in five French groups: importance of regional sampling. Eur J Hum Genet 12: 293-300.
123. Piercy R, Sullivan KM, Benson N, Gill P (1993) The application of mitochondrial DNA typing to the study of white Caucasian genetic identification. Int J Legal Med 106: 85-90.
124. Jin HJ, Tyler-Smith C, Kim W (2009) The peopling of Korea revealed by analyses of mitochondrial DNA and Y-chromosomal markers. PLoS One 4: e4210.
125. Li H, Cai X, Winograd-Cort ER, Wen B, Cheng X, et al. (2007) Mitochondrial DNA diversity and population differentiation in southern East Asia. Am J Phys Anthropol 134: 481-488.
126. Hill C, Soares P, Mormina M, Macaulay V, Clarke D, et al. (2007) A mitochondrial stratigraphy for island southeast Asia. Am J Hum Genet 80: 29-43.
127. Macaulay V, Hill C, Achilli A, Rengo C, Clarke D, et al. (2005) Single, rapid coastal settlement of Asia revealed by analysis of complete mitochondrial genomes. Science 308: 1034-1036.
128. Thangaraj K, Singh L, Reddy AG, Rao VR, Sehgal SC, et al. (2003) Genetic affinities of the Andaman Islanders, a vanishing human population. Curr Biol 13: 86-93.
129. Sykes B, Leiboff A, Low-Beer J, Tetzner S, Richards M (1995) The origins of the Polynesians: an interpretation from mitochondrial lineage analysis. Am J Hum Genet 57: 1463-1475.
130. Tajima A, Hayami M, Tokunaga K, Juji T, Matsuo M, et al. (2004) Genetic origins of the Ainu inferred from combined DNA analyses of maternal and paternal lineages. J Hum Genet 49: 187-193.
131. Simonson TS, Xing J, Barrett R, Jerah E, Loa P, et al. (2011) Ancestry of the Iban is predominantly Southeast Asian: genetic evidence from autosomal, mitochondrial, and Y chromosomes. PLoS One 6: e16338.
132. Maruyama S, Nohira-Koike C, Minaguchi K, Nambiar P (2010) MtDNA control region sequence polymorphisms and phylogenetic analysis of Malay population living in or around Kuala Lumpur in Malaysia. Int J Legal Med 124: 165-170.
133. Wong HY, Tang JS, Budowle B, Allard MW, Syn CK, et al. (2007) Sequence polymorphism of the mitochondrial DNA hypervariable regions I and II in 205 Singapore Malays. Leg Med (Tokyo) 9: 33-37.
134. Tommaseo-Ponzetta M, Attimonelli M, De Robertis M, Tanzariello F, Saccone C (2002) Mitochondrial DNA variability of West New Guinea populations. Am J Phys Anthropol 117: 49-67.

135. Friedlaender JS, Gentz F, Green K, Merriwether DA (2002) A cautionary tale on ancient migration detection: mitochondrial DNA variation in Santa Cruz Islands, Solomon Islands. Hum Biol 74: 453-471.
136. Kayser M, Choi Y, van Oven M, Mona S, Brauer S, et al. (2008) The impact of the Austronesian expansion: evidence from mtDNA and Y chromosome diversity in the Admiralty Islands of Melanesia. Mol Biol Evol 25: 1362-1374.
137. Ohashi J, Naka I, Tokunaga K, Inaoka T, Ataka Y, et al. (2006) Brief communication: mitochondrial DNA variation suggests extensive gene flow from Polynesian ancestors to indigenous Melanesians in the northwestern Bismarck Archipelago. Am J Phys Anthropol 130: 551-556.
138. Ricaut FX, Thomas T, Arganini C, Staughton J, Leavesley M, et al. (2008) Mitochondrial DNA variation in Karkar Islanders. Ann Hum Genet 72: 349-367.
139. Hagelberg E, Goldman N, Lio P, Whelan S, Schiefenhovel W, et al. (1999) Evidence for mitochondrial DNA recombination in a human population of island Melanesia. Proc Biol Sci 266: 485-492.
140. Chiaroni J, King RJ, Myres NM, Henn BM, Ducourneau A, et al. (2009) The emergence of Y-chromosome haplogroup J1e among Arabic-speaking populations. Eur J Hum Genet 18: 348-353.
141. Tofanelli S, Ferri G, Bulayeva K, Caciagli L, Onofri V, et al. (2009) J1-M267 Y lineage marks climate-driven pre-historical human displacements. Eur J Hum Genet 17: 1520-1524.
142. Mirabal S, Varljen T, Gayden T, Regueiro M, Vujovic S, et al. (2010) Human Y-chromosome short tandem repeats: a tale of acculturation and migrations as mechanisms for the diffusion of agriculture in the Balkan Peninsula. Am J Phys Anthropol 142: 380-390.
143. Coelho M, Sequeira F, Luiselli D, Beleza S, Rocha J (2009) On the edge of Bantu expansions: mtDNA, Y chromosome and lactase persistence genetic variation in southwestern Angola. BMC Evol Biol 9: 80.
144. Blanco-Verea A, Jaime JC, Brion M, Carracedo A (2009) Y-chromosome lineages in native South American population. Forensic Sci Int Genet 4: 187-193.
145. King TE, Bowden GR, Balaresque PL, Adams SM, Shanks ME, et al. (2007) Thomas Jefferson's Y chromosome belongs to a rare European lineage. Am J Phys Anthropol 132: 584-589.
146. Zhong H, Shi H, Qi XB, Duan ZY, Tan PP, et al. (2011) Extended Y chromosome investigation suggests postglacial migrations of modern humans into East Asia via the northern route. Mol Biol Evol 28: 717-727.
